# Heterometallic 3‑D Zn/Ca Metal–Organic Frameworks Based on V‑Shaped Angular Tetracarboxylic Ligands as Selective Fluorescence Sensors for Nitroaromatic Explosive Vapors

**DOI:** 10.1021/acs.inorgchem.5c03921

**Published:** 2025-12-16

**Authors:** Rafail P. Machattos, Nikos Panagiotou, Francisco G. Moscoso, Juan Jesús Romero Guerrero, Konstantinos G. Froudas, Pantelis N. Trikalitis, José M. Pedrosa, Anastasios J. Tasiopoulos

**Affiliations:** † Department of Chemistry, 54557 University of Cyprus, 1678 Nicosia, Cyprus; ‡ Center for Nanoscience and Sustainable Technologies (CNATS), Departamento de Sistemas Físicos, Químicos y Naturales, 16772 Universidad Pablo de Olavide, Ctra. Utrera km. 1, 41013 Seville, Spain; § Department of Chemistry, University of Crete, 71003 Heraklion, Greece

## Abstract

A family of heterometallic Zn/Ca MOFs based on angular tetracarboxylic ligands with formulas [ZnCa­(L)­(S)­(S′)]_*n*_ (S, S′ = H_2_O or S = H_2_O, S′ = DMF) **UCY-18**(L) (H_4_L = 4,4′-hexafluoroisopropilidene diphthalic acid (H_4_HFPD), 3,3′,4,4′-benzophenone tetracarboxylic acid (H_4_BPTC), 4,4′-oxydiphthalic acid (H_4_ODPA), 4,4′-azanediyl diphthalic acid (H_4_ADPA)) is reported. The crystal structures of **UCY-18**(L) comprise 3-D networks based on helical one-dimensional chain SBUs [ZnCa­(L^4–^)] containing tetragonal channels along the crystallographic *a*-axis. Gas sorption studies on activated **UCY-18**(L) indicated appreciable BET surface areas ranging from 1338 to 2134 m^2^ g^–1^. Selected vapor sorption studies indicated the affinity of these materials toward representative C_6_-aromatic and nonaromatic organic molecules. Thin films of **UCY-18**(HFPD), **UCY-18**(ODPA), and **UCY-18**(ADPA) embedded in poly­(vinylidene fluoride) were fabricated and evaluated for their sensing capability for vapors of selected nitroaromatic compounds. The thin films exhibit high selectivity in detecting 2,4,6-trinitrophenol and 2,4,6-trinitrotoluene vapors among other nitroaromatic compounds and interferents at equal concentration. The crystal structures of **UCY-18**(HFPD) loaded with nitrobenzene and 2-nitrotoluene provided useful information about the interactions of the MOFs with these nitroaromatic molecules. Overall, this work highlights the potential of heterometallic Zn/Ca MOF chemistry to afford microporous MOFs containing highly accessible metal centers and displaying the capability to recognize vapors of selected analytes.

## Graphical abstract

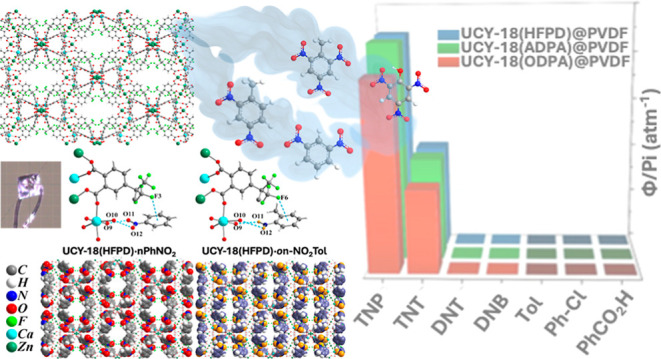

## Introduction

Metal–organic frameworks (MOFs) have gained rapidly increasing research interest since their discovery in the late 20th century. A plethora of materials can be designed and synthesized as a result of the vast library of organic linkers and metal ions, displaying targeted properties. A series of structural characteristics can be manipulated either through de novo synthesis or post synthetic modification reactions including network topology, secondary building units (SBUs), organic linkers, or functional groups,^1–5^ making MOFs a very fruitful platform for several applications. As a result, this class of metal complexes has provided excellent candidate materials for important potential applications in areas of global interest including gas storage/separation,^6–10^ catalysis,^11–13^ sensing,^14–17^ removal of pollutants from the environment,^18–23^ and water harvesting.^24–26^

The construction of functional MOFs involves the careful choice of the metal ions and the organic ligands. In particular, the selection of the suitable carboxylate organic linker for MOF construction depends on several factors, such as the organic linker’s overall size, its rigidity or flexibility, and the number and arrangement of the carboxylate groups. A commonly employed group of organic linkers in MOF synthesis consists of semirigid, V-shaped dicarboxylic acids, which typically incorporate two benzoate moieties connected through a central functional group or atom. In fact, there is a series of analogous dicarboxylic ligands containing different functional groups. These can either display a structure directing capability allowing the formation of different MOF structures under the same reaction conditions^27^ or lead to analogous porous MOFs possessing different functional groups and as a result different or modified properties. Their use in MOF chemistry has led to numerous compounds, based on a wide variety of metal ions, displaying unique structural characteristics and interesting properties. Our group has also employed such ligands for the synthesis of Zr^4+^, 3d, and 4f ion MOFs,^27–31^ which displayed microporous structures and various potential applications. These included the capture of toxic metal ions (Cd^2+^, UO_2_^2+^)^30,31^ and also the sensing of temperature,^29^ metal ions,^30^ and vapors of nitroaromatic and volatile organic compounds (VOCs).^28^ A similar situation also appears with tetracarboxylic ligands, although they have not been used as widely in MOF chemistry. For example, there are a series of analogous angular tetracarboxylic ligands containing two phthalic acid moieties linked through a central atom or functional group.^32–37^ Such ligands, have multiple coordination sites (the four carboxylic groups) and capability to stabilize not only homometallic compounds but also heterometallic ones.^34,37,38^ The latter are also of significant interest since they consist of more than one metal ion displaying different structural characteristics including coordination number/geometry and bridging/coordinative capability and can afford a variety of structures with different SBUs and topologies and unique characteristics. Among them, the ones based on diamagnetic metal ions including alkali/alkaline earth and d^10^ ions have attracted attention due to their ability of producing captivating structures.^38^ Although several heterometallic compounds containing various metal ions have been reported, an attractive combination would include Zn^2+^ due to its flexible coordination sphere/geometry arising from its capability to stabilize both octahedral and tetrahedral coordination geometries with an alkaline earth one as Ca^2+^ that displays high affinity for O-donor atoms. An additional advantage, when emissive compounds are targeted, from the presence of closed-shell configuration metal ions such as Ca^2+^ and Zn^2+^, comes from the lack of potential quenching stemming from d–d transitions, which enables efficient luminescent emission. Such emissive porous MOFs are ideal candidates for use in gas sensing applications.^39,40^

Our group has previously employed a family of lanthanide MOFs based on the angular dicarboxylic ligand 4,4′-sulfonyl dibenzoic acid (H_2_SDBA) for the detection of vapors of nitroaromatic and volatile organic compounds (VOCs).^28^ The development of methods and materials for the detection of vapors of nitroaromatic compounds is of increasing interest. In fact, sensing of nitroaromatic vapors from solid samples is a realistic approach and requires highly sensitive materials due to the extremely low vapor pressure of these compounds.^41,42^ Moreover, it is surprising that TNT is not commonly employed as target analyte,^43–46^ despite being a paradigmatic example of nitroaromatic explosive.

Further efforts targeted the synthesis and use in gas sensing studies of a family of analogous porous MOFs containing different functional groups based on angular tetracarboxylic ligands and also on mixed metal MOFs based on diamagnetic metal ions. Interestingly, the use of such heterometallic MOFs in gas sensing studies is very uncommon. In fact, the presence of two or more different metal centers introduces synergetic effects, such as multiple active sites, tunable pore environments, and enhanced framework stability, which can lead to improved sensitivity and selectivity toward specific gas analytes.^17,47,48^ For all these reasons, it was targeted the synthesis of mixed metal Zn/Ca MOFs based on angular tetracarboxylic ligands containing diphthalic acid moieties linked though a central functional group or an atom.

We herein report the synthesis and characterization of a new family of 3D heterometallic Zn/Ca MOFs based on the angular tetracarboxylic diphthalic ligands 4,4′-hexafluoroisopropilidene diphthalic acid (H_4_HFPD), 3,3′,4,4′-benzophenone tetracarboxylic acid (H_4_BPTC), 4,4′-oxydiphthalic acid (H_4_ODPA), and 4,4′-azanediyl diphthalic acid (H_4_ADPA) with general formulas [ZnCa­(L)­(S)­(S′)]_*n*_ (S, S′ = H_2_O and H_4_L = H_4_HFPD **UCY-18**(HFPD); S = H_2_O, S′ = DMF and H_4_L = H_4_BPTC **UCY-18**(BPTC), H_4_L = H_4_ODPA **UCY-18**(ODPA), H_4_L = H_4_ADPA **UCY-18**(ADPA)). Compounds **UCY-18**(L) (H_4_L = H_4_HFPD, H_4_BPTC, H_4_ODPA, H_4_ADPA) are unique examples of Zn/Ca microporous 3D MOFs based on angular diphthalic tetracarboxylic ligands. Gas sorption studies of compounds **UCY-18**(L) (H_4_L = H_4_HFPD, H_4_BPTC, H_4_ODPA, H_4_ADPA) indicated appreciable BET surface areas of 1523, 2070, 2134, and 1338 m^2^ g^–1^, respectively, and CO_2_ sorption capabilities, up to 5 mmol g^–1^ at 273 K, 3.8 mmol g^–1^ at 283 K, and 2.3 mmol g^–1^ at 298 K for **UCY-18**(ADPA). Moreover, gas sensing PL studies on PVDF-based films of **UCY-18**(HFPD), **UCY-18**(ODPA), and **UCY-18**(ADPA) {**UCY-18**(HFPD)@PVDF, **UCY-18**(ODPA)@PVDF and **UCY-18**(ADPA)@PVDF} indicated different responses in the presence of various nitroaromatic compounds and high selectivity in detecting 2,4,6-trinitrophenol (TNP) and 2,4,6-trinitrotoluene (TNT) vapors among other nitroaromatic compounds and interferents. Finally, single-crystal-to-single-crystal (SCSC) exchange reactions of activated **UCY-18**(HFPD) upon exposure to vapors of nitrobenzene (PhNO_2_) and 2-nitrotoluene (*o*-NO_2_Tol) provided useful insights on the interactions of the guest nitroaromatic molecules with the frameworks of the MOFs, which may be responsible for their facile insertion in the pores of the materials and the observed PL spectral changes.

## Experimental Details

### Materials

Reagent grade chemicals were obtained from commercial sources (Aldrich, Merck, Alfa Aesar, TCI, BLD pharm, etc.) and used without further purification. All synthetic procedures were carried out in air.

2,4-Dinitrotoluene (DNT), 1,3-dinitrobenzene (DNB), and 2,4,6-trinitrophenol (TNP) were purchased from Sigma-Aldrich. 2,4,6-Trinitrotoluene (TNT) was synthesized from DNT following the procedure proposed by Guillén et al.^49^ Caution! Nitroaromatic compounds (DNB, DNT, TNT, and TNP) are potentially hazardous and must be handled with extreme care. All manipulations involving these substances should be conducted in a well-ventilated fume hood, using appropriate personal protective equipment (PPE), indicating gloves and safety goggles. Due to their toxic, potentially explosive, and environmentally harmful nature, strict adherence to institutional safety protocols is essential. Poly­(vinylidene fluoride) (PVDF) with an average molecular weight of 1,000,000 Da was purchased from BLD pharm. Other chemical reagents and solvents were of HPLC grade and used without further purification.

### Synthesis

Synthesis of **UCY-18**(L) (H_4_L = H_4_HFPD, H_4_BPTC, H_4_ODPA, H_4_ADPA): In a 20 mL glass vial containing DMF (5 mL) was added the corresponding anhydride (4,4′-HFPD, 3,3′,4,4′-BPTD or 4,4′-ODPA) of diphthalic tetracarboxylic acids H_4_HFPD, H_4_BPTC, or H_4_ODPA (0.23 mmol) or the tetracarboxylic acid H_4_ADPA (0.079 g, 0.23 mmol), and the resulting solution was placed in an ultrasonic bath for 5 min. Then deionized water (2 mL), HNO_3_ 65% (25 μL, 0.023 g, 0.36 mmol), HCOOH 90% (25 μL, 0.027 g, 0.59 mmol), and solids Zn­(NO_3_)_2_·6H_2_O (0.060 g, 0.20 mmol) and Ca­(NO_3_)_2_·4H_2_O (0.048 g, 0.20 mmol) were subsequently added. The reaction mixture was sonicated again for 5 min, sealed with a plastic cap, and left undisturbed in an oven at 100 °C for 24 h. The large colorless octahedral crystals of **UCY-18**(L) (H_4_L = H_4_HFPD, H_4_BPTC, H_4_ODPA, H_4_ADPA) formed were isolated by filtration, washed several times with DMF, and dried under vacuum. The reaction yields were in the range of 75–85% based on Zn­(NO_3_)_2_·6H_2_O. Anal. Calcd for **UCY-18**(HFPD)·5nDMF (ZnCaF_6_O_15_N_5_C_34_H_45_): C, 41.54; H, 4.61; N, 7.12. Found: C, 41.87; H, 4.80; N, 7.42. **UCY-18**(BPTC)·5nDMF (ZnCaO_16_N_6_C_35_H_50_): C, 45.88; H, 5.50; N, 9.17. Found: C, 46.12; H, 5.73; N, 9.35. **UCY-18**(ODPA)·6nDMF (ZnCaO_17_N_7_C_37_H_57_): C, 45.47; H, 5.88; N, 10.03. Found: C, 45.77; H, 6.05; N, 9.82. **UCY-18**(ADPA)·7nDMF (ZnCaO_17_N_9_C_40_H_65_): C, 45.78; H, 6.24; N, 12.01. Found: C, 45.49; H, 6.03; N, 12.22.

Synthesis of **UCY-18**(HFPD)·nitroaromatic (nitroaromatic = PhNO_2_, *o*-NO_2_Tol): Single crystals of activated **UCY-18**(HFPD) were placed in a 20 mL glass vial. Then, a 4 mL glass vial containing a selected nitroaromatic compound was placed inside the 20 mL glass vial, and the two vials were left undisturbed at 30 °C for 1 week to form the exchanged analogue of **UCY-18**(HFPD) loaded with a nitroaromatic compound. Anal. Calcd for **UCY-18**(HFPD)·2.5nPhNO_2_ (ZnCaF_6_O_15_N_2.5_C_34_H_22.5_): C, 44.12; H, 2.45; N, 3.78. Found: C, 44.35; H, 2.71; N, 4.01. **UCY-18**(HFPD)·2n*o*-NO_2_Tol (ZnCaF_6_O_14_N_2_C_33_H_24_): C, 44.44; H, 2.71; N, 3.14. Found: C, 44.66; H, 2.96; N, 3.00.

### **UCY-18**(L)@PVDF (H_4_L = H_4_HFPD, H_4_BPTC, H_4_ODPA, H_4_ADPA) Film Preparation

50 mg of activated MOF was suspended with 1 mL of acetone (HPLC grade). The suspension was mixed with a solution of PVDF in DMF with a concentration of 7.5% wt. The mixture was sonicated and left under magnetic stirring until the MOF was uniformly dispersed. The acetone was then removed in a rotary evaporator, and the resulting mixture was spin-coated in a Petri dish at 500 rpm for 10 s. The films were cured at 65 °C for an hour. Finally, the membrane was delaminated by immersing it in warm deionized water and cutting it, as required.

### Physical Measurements

Elemental analyses (C, H, and N) were performed by the in-house facilities of the University of Cyprus, Chemistry Department. IR spectra were recorded on ATR in the 4000–700 cm^–1^ range using a Shimadzu Prestige – 21 spectrometer. Powder X-ray diffraction patterns were recorded on a Rigaku Miniflex 6G X-ray diffractometer (Cu Kα radiation, λ = 1.5418 Å). Variable temperature pXRD (VT-pXRD) measurements were recorded on a Rigaku Miniflex 6G X-ray diffractometer using a BTS 500 high-temperature attachment under Ar flow with an increase rate of 5 °C/min in the range of 25–500 °C. Thermal stability studies were performed with a Shimadzu TGA 50 thermogravimetric analyzer. ^1^H NMR spectra were recorded on a Bruker Avance III 300 MHz spectrometer at 25 °C. Solid-state PL measurements for **UCY-18**(L) (H_4_L = H_4_HFPD, H_4_BPTC, H_4_ODPA, H_4_ADPA) were carried out on a JASCO FP-8300 spectrofluorometer.

The MOF-based membrane topologies were analyzed by using a ZEISS GeminiSEM 300 scanning electron microscope. To enhance sample conductivity, the membranes were coated with 10 nm of a gold layer by applying a metal sputter (DSCT, Vac Coat). PL emission and excitation spectra of membranes were recorded with an FLS1000 Photoluminescence Spectrophotometer (Edinburgh Instruments). X-ray microdiffraction (μ-XRD) patterns of the membranes were collected using a Discover D8 (Bruker) diffractometer with Cu Kα radiation (1.5406 Å, 50 kV, 1 mA) in the 4–35° 2θ range with a step of 0.02° per 0.2 s.

### Gas/Vapor Adsorption

Low pressure gas sorption measurements were carried out at different temperatures using an Autosorb-iQ3 by Quantachrome system equipped with a cryocooler capable of thermostatting up to 2 samples in the temperature range of 20 to 320 K. Prior to analysis, the as-synthesized samples were washed with *N*,*N*-dimethylformamide four times for 1 day and then soaked in acetone. Then, acetone was exchanged 3 times per day for 2 weeks. Finally, the wet samples were transferred to 6 mm sample cells and activated under the outgas dynamic vacuum at room temperature for 20 h until the outgas rate was less than 2 mTorr min^–1^. After evacuation, the samples were reweighed to obtain the precise mass of the evacuated samples, and the cells were transferred to the analysis port of the gas sorption instrument. Vapor sorption isotherms for *n*-hexane, cyclohexane, and benzene were recorded at 298 K up to 1 bar, using a state-of-the-art, high-precision BELSORP-maxII from Microtrac MRB, equipped with (4) analysis stations and a detachable thermostatic bath for accurate measurements. Prior to measurements, each vapor was degassed to remove any dissolved gases following a standard protocol. For comparison purposes, all isotherms are presented as the amount adsorbed as a function of the relative pressure, *p*/*p*_0_, where *p*_0_ is the saturation pressure of the vapor at the measurement temperature.

### Single-Crystal X-ray Crystallography

Single-crystal X-ray diffraction data were collected on a Rigaku Supernova A diffractometer, equipped with a CCD area detector utilizing Cu Kα (λ = 1.5406 Å) and Mo Kα (λ = 0.7107 Å) radiation, and on a Rigaku XtaLAB Synergy S diffractometer, equipped with a HyPix-6000HE detector utilizing Cu Kα (λ = 1.5406 Å) radiation. A suitable crystal was mounted on a Hampton cryoloop with paratone-N oil and transferred to a goniostat, where it was cooled for data collection. The structures were solved by direct methods using SHELXT and refined on *F* ^2^ using full-matrix least-squares using SHELXL14.1.^50^ Software packages used: CrysAlis CCD for data collection, CrysAlis RED for cell refinement and data reduction,^51^ WINGX and Olex2 for geometric calculations,^52,53^ and DIAMOND for molecular graphics.^54^ In order to limit the disorder of the terminal or the lattice solvent molecules, various restraints (SIMU, RIGU, DELU, DFIX, DANG, FLAT, ISOR) have been applied in the refinement of the crystal structures. The non-H atoms were treated anisotropically, whereas the aromatic hydrogen atoms were placed in calculated, ideal positions and refined as riding on their respective carbon atoms. Electron density contributions from disordered guest molecules were handled using the SQUEEZE procedure from the PLATON software suit^55^ due to the disordered nature of these molecules. Selected crystal data and bond lengths for **UCY-18**(L) (H_4_L = H_4_HFPD, H_4_BPTC, H_4_ODPA, H_4_ADPA), **UCY-18**(HFPD)·nPhNO_2_, and **UCY-18**(HFPD)·n*o*-NO_2_Tol are summarized in Tables S1–S6 in the Supporting Information, SI. CCDC 2475909–2475914 contain the supplementary crystallographic data for this paper. Full details can be found in the CIF files provided as SI.

### Sensing Assays

For sensing measurements, the MOF-based membranes were placed in a glass vial that was previously saturated with vapors from 10 to 15 mg of the different nitroaromatic compounds, including 1,3-dinitrobenzene (DNB), 2,4-dinitrotoluene (DNT), 2,4,6-trinitrotoluene (TNT), and 2,4,6-trinitrophenol (TNP). The vial was then hermetically sealed. Due to the extremely low vapor pressure of these compounds, the exposure was continued for 24 h to ensure complete saturation. All experiments were carried out at room temperature. The calculated concentrations (v/v) at room temperature (25 °C) for the four nitroaromatics were 1.16 ppm (DNB), 346 ppb (DNT), 7.32 ppb (TNT), and 0.98 ppb (TNP).

## Results and Discussion

### Synthesis and Crystal Structure

**UCY-18**(L) (H_4_L = H_4_HFPD, H_4_BPTC, H_4_ODPA, or H_4_ADPA) were synthesized from the reaction of Zn­(NO_3_)_2_ and Ca­(NO_3_)_2_ with the corresponding anhydride (4,4′-HFPD, 3,3′,4,4′-BPTD, or 4,4′-ODPA) of the diphthalic tetracarboxylic acids H_4_HFPD, H_4_BPTC, and H_4_ODPA, or the tetracarboxylic acid H_4_ADPA in a 1:1:1.15 molar ratio in DMF/H_2_O in the presence of ∼2.75 equivalents of concentrated HNO_3_ and ∼3 equivalents of concentrated HCOOH. The structures of the anhydrides and the corresponding ligands are shown in Scheme 1, while the synthetic route and characterization of H_4_ADPA are provided in Schemes S1 and S2. The reaction mixtures were placed in an oven at 100 °C for 1 day and afforded large octahedral crystals (colorless for compounds **UCY-18**(HFPD), **UCY-18**(BPTC), and **UCY-18**(ODPA) and orange for compound **UCY-18**(ADPA)).

**Scheme 1:**
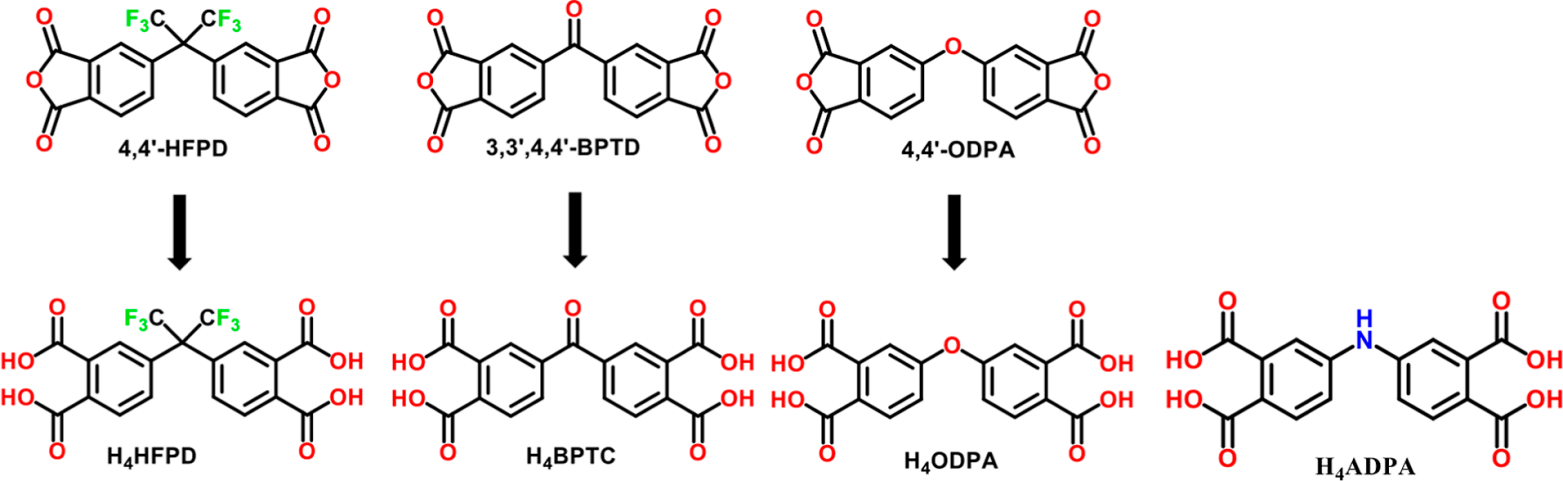
Angular Dianhydrides and the Tetracarboxylic Ligand (H_4_ADPA) That Were Employed in the Reaction Mixtures Afforded Compounds **UCY-18**(L) (H_4_L = H_4_HFPD, H_4_BPTC, H_4_ODPA, H_4_ADPA)^a^

a The tetracarboxylic ligands H_4_HFPD, H_4_BPTC, and H_4_ODPA were formed from the in situ hydrolysis of the dianhydrides.

Compounds **UCY-18**(L) (H_4_L = H_4_HFPD, H_4_BPTC, H_4_ODPA, H_4_ADPA) feature neutral three-dimensional porous frameworks that are isoreticular and crystallize in the tetragonal space group *I*4̅2*d*. The four compounds display a significant similarity, with their main difference being the central functional group that connects the two phthalic acid moieties. For this reason, only the structure of compound **UCY-18**(HFPD) will be discussed in detail; structural differentiations of the structure of **UCY-18**(HFPD) with those of the other 3 compounds shall be indicated. Compound **UCY-18**(HFPD) contains two crystallographically independent Zn^2+^ ions (with occupancy 0.5 each), one Ca^2+^ ion and a deprotonated HFPD^4–^ ligand. Representations of the structure of **UCY-18**(HFPD) are shown in Figure 1 and those of **UCY-18**(L) (H_4_L = H_4_BPTC, H_4_ODPA, H_4_ADPA) are shown in Figures S1–S3. The asymmetric unit of **UCY-18**(HFPD) consists of a 1-D helical chain with the molecular formula [ZnCa­(COO^–^)_4_] (Figure 1a). The coordination sphere of each Zn^2+^ ion consists of four oxygen atoms from the carboxylates of four different HFPD^4–^ ligands, which adopt a tetrahedral geometry. The coordination sphere of each Ca^2+^ ion consists of four oxygen atoms from the carboxylates of four different HFPD^4–^ ligands and two oxygen atoms from two terminally ligated water molecules, adopting a distorted octahedral coordination geometry (whereas in the case of compounds **UCY-18**(BPTC), **UCY-18**(ODPA), and **UCY-18**(ADPA), the terminal ligation is provided by water and DMF molecules). The crystallographically independent HFPD^4–^ ligand connects eight metal ions in a η^1^:η^1^:η^1^:η^1^:η^1^:η^1^:η^1^:η^1^:μ_8_ fashion (Figure 1b). A close examination of the connection of the 1-D chains through HFPD^4–^ ligands revealed the formation of tetragonal-shaped channels along the crystallographic *a*-axis in which the trifluoromethyl groups (−CF_3_) are oriented toward the outer surface of the channels (Figure 1c). The diameter of the channels was found equal to 11 Å (14 Å for compounds **UCY-18**(BPTC), **UCY-18**(ODPA), and **UCY-18**(ADPA)). The solvent accessible volume of compound **UCY-18**(HFPD) was calculated by PLATON (after omitting the terminally ligated solvent molecules) to 62.5% of the unit cell volume (73.6%, 74.6%, and 75.6% for **UCY-18**(BPTC), **UCY-18**(ODPA), and **UCY-18**(ADPA), respectively).^55^

**Figure 1:**
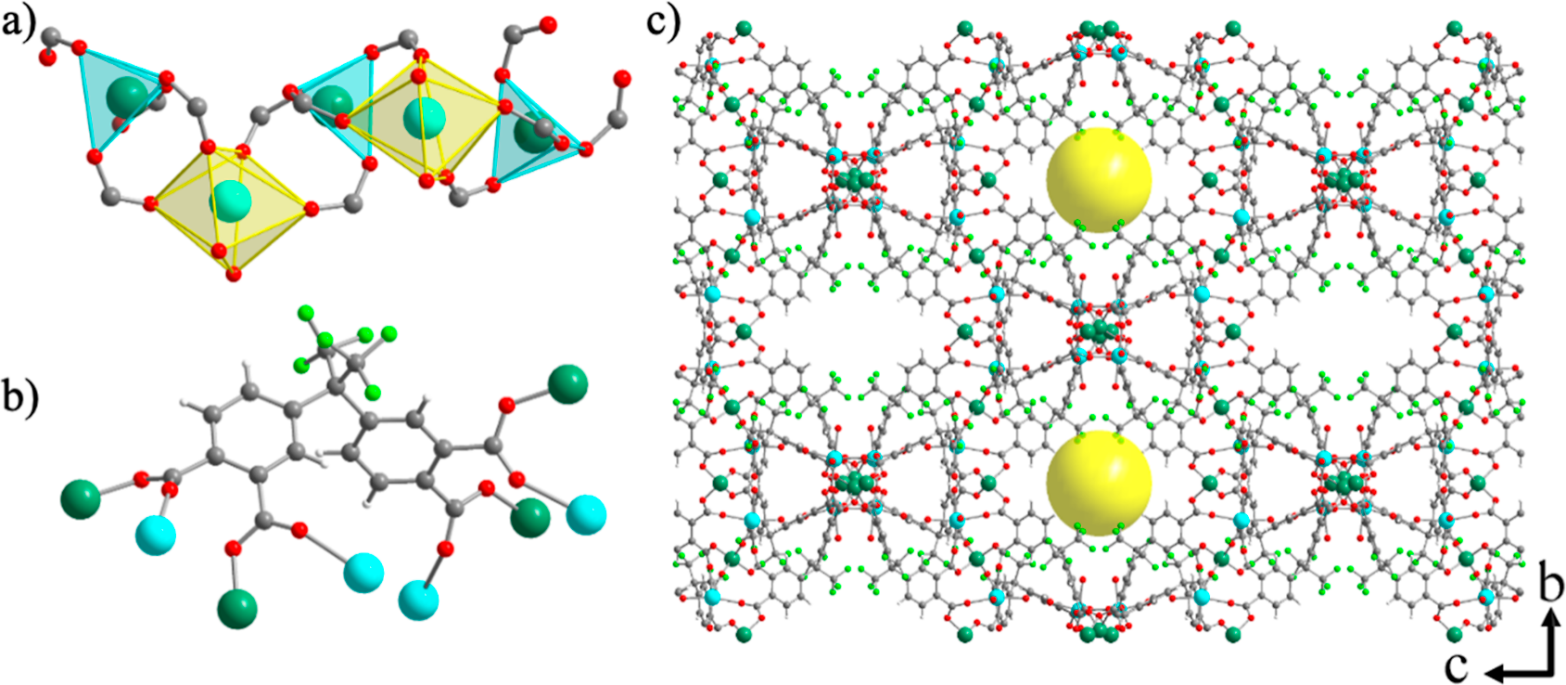
Representations of the (a) 1-D helical chain [ZnCa­(COO)_4_] SBU, (b) the η^1^:η^1^:η^1^:η^1^:η^1^:η^1^:η^1^:η^1^:μ_8_ coordination mode of the crystallographically independent HFPD^4–^ ligand, and (c) packing of the 3-D structure along the *a*-axis emphasizing on the intralayer tetragonal channels of compound **UCY-18**(HFPD). The yellow spheres (shown in (c)) denote the pores formed along *a*-axis. Color code: Zn, dark green; Ca, turquoise; F, light green; O, red; C, gray; H, white.

A CCDC search for Zn/Ca heterometallic MOFs based on polycarboxylic ligands returned 16 examples,^39,40,48,56–64^ one of which is based on a hexacarboxylic ligand, four on tetracarboxylic ligands, two on tricarboxylic ligands, and the remaining are based on dicarboxylic ligands. Notably, the reported MOFs contain the highest solvent accessible volumes and BET areas among the known MOFs of this family. Thus, these results highlight the potential of heterometallic Zn/Ca MOF chemistry to afford compounds with significant BET areas that could be excellent candidates for various applications (see Table S7 in SI).

The stability of compounds **UCY-18**(L) (H_4_L = H_4_HFPD, H_4_BPTC, H_4_ODPA, H_4_ADPA) in common organic solvents was examined by pXRD. These studies revealed that the crystallinity and structural integrity of the compounds is retained upon exposure to air as well as after treatment with selected, mainly low-polarity, organic solvents (Figures S4–S7). Also, their IR spectra are shown in Figures S8–S11.

The thermal stability of compounds **UCY-18**(L) (H_4_L = H_4_HFPD, H_4_BPTC, H_4_ODPA, H_4_ADPA) was investigated by means of thermogravimetric analysis (Figures S12–S15) and variable temperature pXRD of samples treated with acetone (Figures S16–S19). Their thermal decomposition includes continuous mass losses. These are attributed to the removal of terminally ligated and guest solvent molecules (DMF/H_2_O) that is completed at temperatures range of up to ∼350–380 °C and the combustion of the tetracarboxylic ligand that is completed above ∼550 °C. The residual mass at 900 °C corresponds to an equimolar mixture of ZnO and CaO (Figures S12–S15). A more detailed discussion of the TGA studies for each MOF is included in SI. Variable temperature pXRD studies revealed that **UCY-18**(L) (H_4_L = H_4_HFPD, H_4_BPTC, H_4_ODPA, H_4_ADPA) retain their crystallinity and structural integrity up to ∼100–150 °C, depending on the compound (Figures S16–S19).

### Gas Sorption Properties

The activation of the materials was performed through exchange of the lattice and coordinated solvent molecules with acetone (see the experimental part). The exchange process led to the complete removal of DMF (as confirmed by the ^1^H NMR spectra of the treated with acetone **UCY-18**(L) (H_4_L = H_4_HFPD, H_4_BPTC, H_4_ODPA, H_4_ADPA) digested in DCl (35% wt in D_2_O) shown in Figures S20–S23) and the exchanged with acetone **UCY-18**(L) MOFs retain their crystallinity and structural integrity, as confirmed by pXRD studies (Figures S24–S27). Argon sorption measurements of the activated compounds **UCY-18**(L) at 87K revealed type-I isotherms (Figure 2a), typical for microporous solids, from which the apparent BET surface areas were found to be 1523 m^2^ g^–1^ (Langmuir 1674 m^2^ g^–1^) for compound **UCY-18**(HFPD), 2070 m^2^ g^–1^ (Langmuir 2494 m^2^ g^–1^) for compound **UCY-18**(BPTC), 2134 m^2^ g^–1^ (Langmuir 2513 m^2^ g^–1^) for compound **UCY-18**(ODPA), and 1338 m^2^ g^–1^ (Langmuir 1674 m^2^ g^–1^) for compound **UCY-18**(ADPA) (Figures S28–S35). The total pore volume values at relative pressure, *p*/*p*_0_ = 0.995, are 0.60 cm^3^ g^–1^ for compound **UCY-18**(HFPD), 0.87 cm^3^ g^–1^ for compound **UCY-18**(BPTC), 0.89 cm^3^ g^–1^ for compound **UCY-18**(ODPA), and 0.63 cm^3^ g^–1^ for compound **UCY-18**(ADPA). The pore volume values for **UCY-18**(HFPD), **UCY-18**(BPTC), and **UCY-18**(ODPA) are in good agreement with the ones calculated with software PoreBlazer^65^ indicating a successful activation of the three MOFs. The pore volume value for **UCY-18**(ADPA) is smaller than the calculated one (∼1.12 cm^3^ g^–1^) due to the difficulty in completely evacuating its pores from DMF solvent molecules without damaging the framework structure. The pore size distribution was calculated using non-local density functional theory (NLDFT) after a successful fitting of the Ar adsorption isotherm data using a suitable NLDFT kernel (Figures S36–S39). All four compounds show three major peaks centered at 6, 14, and 17 Å for compound **UCY-18**(HFPD), 8, 14, and 18 Å for compound **UCY-18**(BPTC) and 9, 14, and 18 Å for both compounds **UCY-18**(ODPA) and **UCY-18**(ADPA) (Figure 2b).

**Figure 2:**
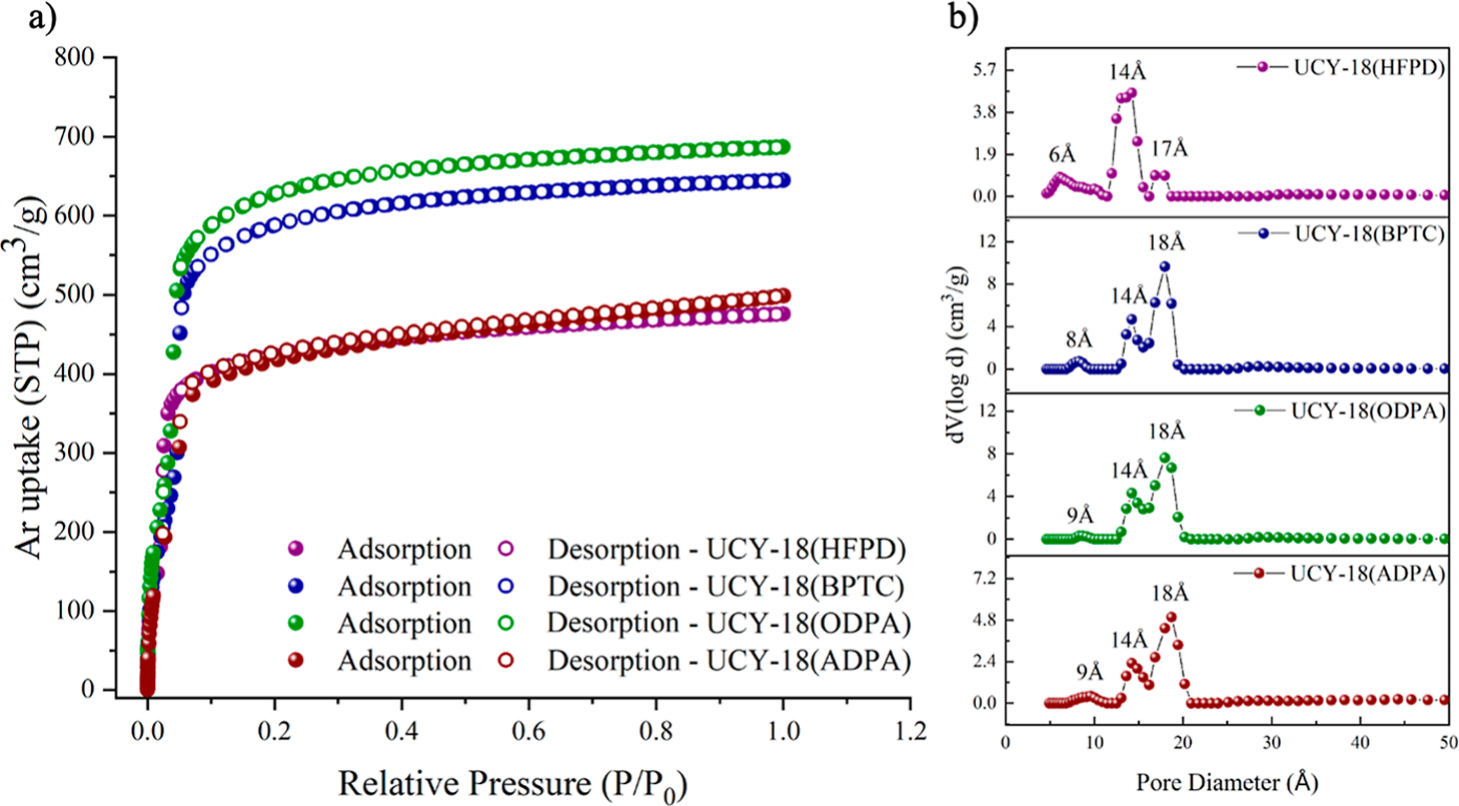
(a) Ar sorption isotherms recorded at 87 K and (b) pore size distribution curves calculated by NLDFT of compounds **UCY-18**(L) (H_4_L = H_4_HFPD, H_4_BPTC, H_4_ODPA, H_4_ADPA).

The microporous structures of the MOFs and the presence of functional groups in their channels prompted us to investigate their gas sorption capability (CO_2_, CH_4_, and H_2_). The gas sorption studies on the activated MOFs were performed at different temperatures and pressures up to 1 bar (Table S8). As shown in Figure S40a–d, the MOFs begin to sorb CO_2_ in the low-pressure region, and the maximum uptake at 273 K reaches 2.1, 2.3, 2.4, and 5.0 mmol g^–1^ for compounds **UCY-18**(L) (H_4_L = H_4_HFPD, H_4_BPTC, H_4_ODPA, H_4_ADPA), respectively. It shall be noted that the obtained CO_2_ sorption capacity is comparable to these of other reported heterometallic Zn/Ca frameworks.^57,60,66^ Among the MOFs of this study, **UCY-18**(ADPA) showed much higher CO_2_ sorption capability compared to the other analogues, probably because of the existence of the –NH– central functional groups of ADPA^4–^ ligand in its pores.^67–70^ Using the isotherms recorded at 273, 283, and 298 K and applying a virial type fitting (Figure S41a–d), the isosteric heat of adsorption, Qst was calculated for **UCY-18**(L) (H_4_L = H_4_HFPD, H_4_BPTC, H_4_ODPA, H_4_ADPA) to 25.0, 25.6, 23.8, and 28.1 kJ mol^–1^ at zero coverage (*Q*_st_^0^), respectively. In addition, *Q*_st_ as a function of surface coverage remains almost constant, suggesting a uniform potential behavior of the compounds (Figures S42a–d). As far as CH_4_ adsorption capability (Figure S43a–d) is concerned, in the low-pressure region, the maximum uptake at 273 K reaches 0.6, 0.6, 0.6, and 1.5 mmol g^–1^ for compounds **UCY-18**(L) (H_4_L = H_4_HFPD, H_4_BPTC, H_4_ODPA, H_4_ADPA), respectively. Using the isotherms recorded at 273, 283, and 298 K and applying a virial type fitting (Figure S44a–d), the isosteric heat of adsorption, *Q*_st_, was calculated to be 24.4, 23.1, 27.3, and 34.5 kJ mol^–1^ at zero coverage (*Q*_st_^0^) for **UCY-18**(L) (H_4_L = H_4_HFPD, H_4_BPTC, H_4_ODPA, H_4_ADPA), respectively, and as a function of surface coverage remains almost constant, suggesting a uniform potential behavior of the compounds (Figures S45a–d). Furthermore, low-pressure H_2_ sorption isotherms, shown in Figure S46a–d, recorded up to 1 bar, revealed an uptake of 93.5, 114.6, 103.6, and 59.2 cm^3^ g^–1^ at 77 K and 59.6, 70.5, 62.5, and 35.0 cm^3^ g^–1^ at 87 K for **UCY-18**(L) (H_4_L = H_4_HFPD, H_4_BPTC, H_4_ODPA, H_4_ADPA), respectively. H_2_ sorption isotherms were fitted using a virial-type eq (Figure S47a–d) and *Q*_st_ at zero coverage was calculated to be 5.70, 5.24, 5.20, and 6.30 kJ mol^–1^ for **UCY-18**(L) (H_4_L = H_4_HFPD, H_4_BPTC, H_4_ODPA, H_4_ADPA), respectively (Figure S48a–d).

### Vapor Sorption Studies

The presence of different functional groups in the pore space of **UCY-18**(L) materials prompted us to investigate the sorption properties of selected vapors including hexane, cyclohexane (Cy), and benzene (Bz). Capture and removal of these hydrocarbons especially at trace levels from indoor environments is very important, and MOFs are highly promising for these applications.^71,72^ The corresponding adsorption isotherms for **UCY-18**(L) materials were recorded at 298 K up to the corresponding saturation pressure of each vapor, and the results are shown in Figure 3. Table 1 summarizes the total uptake at 0.99 *p*/*p*_0_ and the calculated total pore volume, assuming that the density of the adsorbed phase near saturation is that of the corresponding liquid. The isotherms are shown on a logarithmic scale to highlight the low-pressure region, from which important findings are revealed. In particular, for **UCY-18**(BPTC) and **UCY-18**(ODPA), an S-type isotherm with a relatively sharp step is observed for the three vapors consistent with a pore filling mechanism. The relative pressure at which this step occurs for each vapor is associated with the affinity of the framework, where lower values indicate higher affinity. As shown in Figure 3, the pore filling step for hexane is observed at lower relative pressures (∼8 × 10^–3^ *p*/*p*_0_) compared to that of Bz and Cy (both ∼1.5 × 10^–2^ *p*/*p*_0_), indicating a preferable adsorption, which is attributed to the rotational freedom of the sp^3^-type C–C bonds in the hexane molecule, that allows to adjust its relative orientation, providing a high degree of van der Waals contacts with the framework, resulting in stronger adsorption.^73,74^ In contrast, **UCY-18**(HFPD) shows a very different behavior for Bz, where a two-step adsorption isotherm is obtained. Notably, the first step is observed at a significantly lower partial pressure (3 × 10^–3^ *p*/*p*_0_) compared to the single step observed for Cy (1.5 × 10^–2^ *p*/*p*_0_), while the second step is almost identical to that of Cy. This distinct sorption isotherm implies that **UCY-18**(HFPD) has a higher affinity for Bz over Cy that could be associated with favorable −C–F···π interactions. These results strongly suggest that **UCY-18**(HFPD) could be an important material for the highly challenging Bz/Cy separation.^75,76^

**Figure 3:**
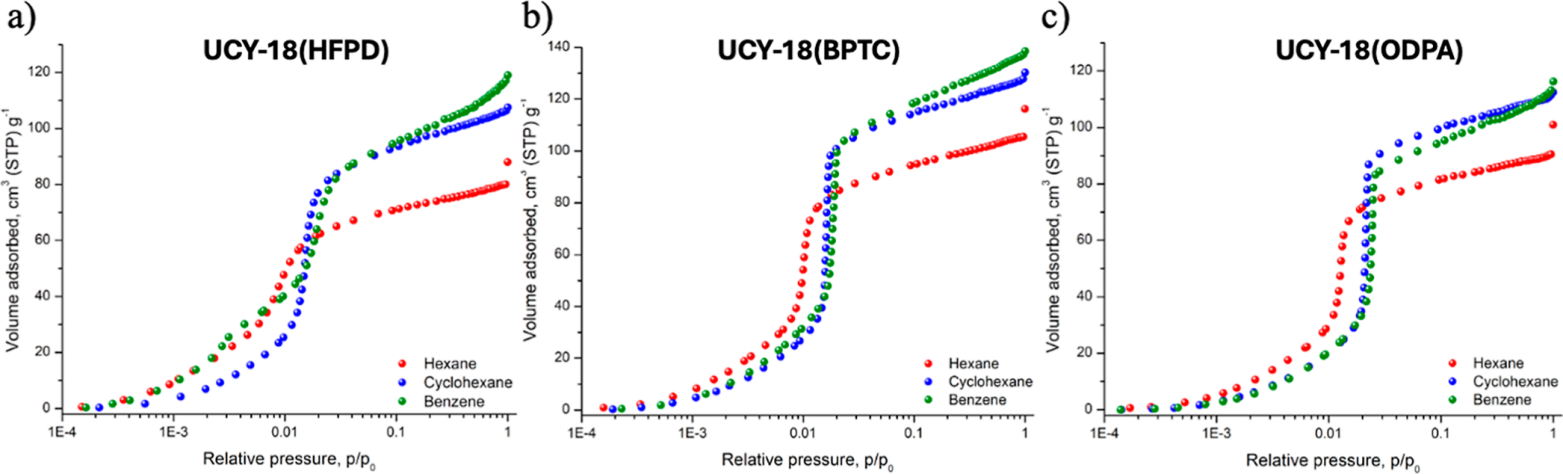
Hexane, benzene, and cyclohexane adsorption isotherms of (a) **UCY-18**(HFPD), (b) **UCY-18**(BPTC), and (c) **UCY-18**(ODPA) recorded at 298 K up to saturation pressure.

**Table 1:** Summary of the Total Uptake and Total Pore Volume of the Different Vapors Obtained from the Corresponding Adsorption Isotherm

	total uptake (cm^3^ g^–1^)/total pore volume (cm^3^ g^–1^)		
sample	hexane	Cy	Bz
**UCY-18**(HFPD)	80.1:0.47	107.5:0.52	119.0:0.47
**UCY-18**(BPTC)	105.5:0.61	130.2:0.63	138.6:0.55
**UCY-18**(ODPA)	90.5:0.53	112.4:0.54	116.2:0.46

### Thin-Film Characterization and Sensing Studies

Room-temperature photoluminescence properties of the pristine compounds **UCY-18**(L) (H_4_L = H_4_HFPD, H_4_BPTC, H_4_ODPA, H_4_ADPA) and the ligands were investigated in the solid state as crystalline powders. Upon excitation at ∼345, 370, 324, and 376 nm, a broad emission band at 450, 450, 350, and 500 nm is observed in the photoluminescence (PL) spectra of 4,4′-HFPD, 3,3′,4,4′-BPTD, 4,4′-ODPA, and H_4_ADPA, respectively, which are attributed to the π* → π transition of the ligands (Figures S49–S52). **UCY-18**(L)@PVDF membranes were fabricated to investigate their gas sensing capability. The PVDF membrane has a high porosity (Figure S53a,b), with an average pore diameter of 2 μm (Figure S53d). The pores are uniformly distributed over the film surface, which makes it an ideal polymeric hosting matrix for the gas sensing application. Furthermore, the cross-sectional image (Figure S53c) reveals that the inside of the membrane has a sponge-like structure, with microvoids that confer a high internal area to the membrane. After embedding the MOF crystals into the PVDF membranes, the porosity of the membranes remains unaltered, as shown in the SEM images of Figure 4, allowing the gas entry and diffusion through the membrane. In addition, the **UCY-18**(L) (H_4_L = H_4_HFPD, H_4_BPTC, H_4_ODPA, H_4_ADPA) membranes displayed a uniform texture and color as shown in the insets of Figure 4. The crystallinity of the MOFs in the PVDF membranes was confirmed through μ-XRD analysis. As observed in the corresponding diffractograms of **UCY-18**(HFPD)@PVDF, **UCY-18**(ODPA)@PVDF, and **UCY-18**(ADPA)@PVDF in Figure S54, the presence of the characteristic peaks of the pristine materials indicates that the MOF particles remain crystalline after being incorporated into the PVDF membranes. Additionally, the quasi-amorphous phase around 2θ = 20° was attributed to the β-phase of the polymeric matrix.^77^ These results demonstrate that the MOFs retain their crystallinity after being incorporated into the membrane.

**Figure 4:**
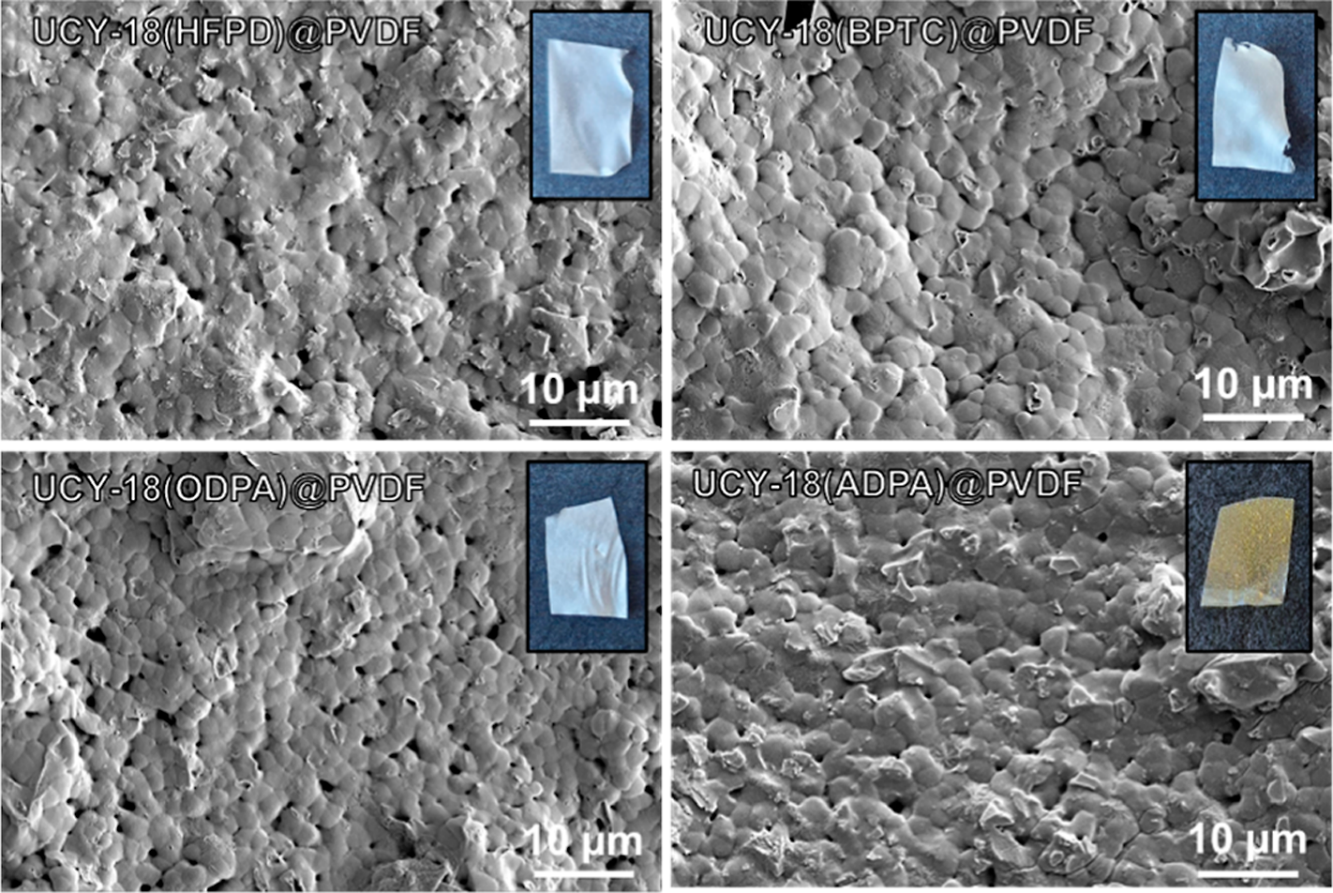
Top-view of **UCY-18**(HFPD)@PVDF, **UCY-18**(BPTC)@PVDF, **UCY-18**(ODPA)@PVDF, and **UCY-18**(ADPA)@PVDF membranes. Inset: photographs of a piece of each membrane with a dimension of 1 × 1.5 cm indicating their uniform texture and color.

The PL emission spectra of **UCY-18**(HFPD)@PVDF, **UCY-18**(ODPA)@PVDF, and **UCY-18**(ADPA)@PVDF upon excitation at 345 nm, 324, and 330 nm, respectively, are shown in Figure 5 (solid black lines). As can be seen, the membranes exhibit the same emission profile as the microcrystalline powder of the corresponding MOFs (Figures S49–S52). Note that **UCY-18**(BPTC)@PVDF thin films were also fabricated, but it was not possible to measure the PL response due to photodegradation of the membrane under UV light. This was visualized by the presence of a shaded region in the thin films following exposure to the incident light beam (upon excitation) (Figure S55). The exposure of the **UCY-18**(HFPD)@PVDF, **UCY-18**(ODPA)@PVDF, and **UCY-18**(ADPA)@PVDF films to saturated vapors of several nitroaromatic compounds (DNB, DNT, TNT, and TNP) was carried out for 24 h to ensure the complete saturation of the sensing response. As can be seen in Figure 5 (solid colored lines), a significant quenching in the emission bands of **UCY-18**(HFPD)@PVDF, **UCY-18**(ODPA)@PVDF, and **UCY-18**(ADPA)@PVDF was obtained in all cases. The relative PL change was quantified as $\Phi =1-\frac{I}{I_{0}}$ , where *I*_0_ is the maximum PL emission intensity of the films, and *I* is the PL emission intensity at the same wavelength after exposure to the analytes. The obtained values for Φ × 100 (%) were in the range of 74–99% and are summarized in Table 2. This table also includes the most relevant literature reports on luminescent MOF-based sensing of the four nitroaromatic vapors investigated in this work. Note that this comparison is restricted to the detection of these nitroaromatics in the gas phase, which is not comparable to the more abundant literature results in liquid media due to the completely different sensing conditions. Interestingly, apart from our previous results, no significant data have been reported for TNT vapor detection, despite it being the most representative explosive. The comparative analysis highlights the superior sensitivity of our MOF membranes toward all four gaseous analytes, including those with lower vapor pressures (TNT and TNP).

**Figure 5:**
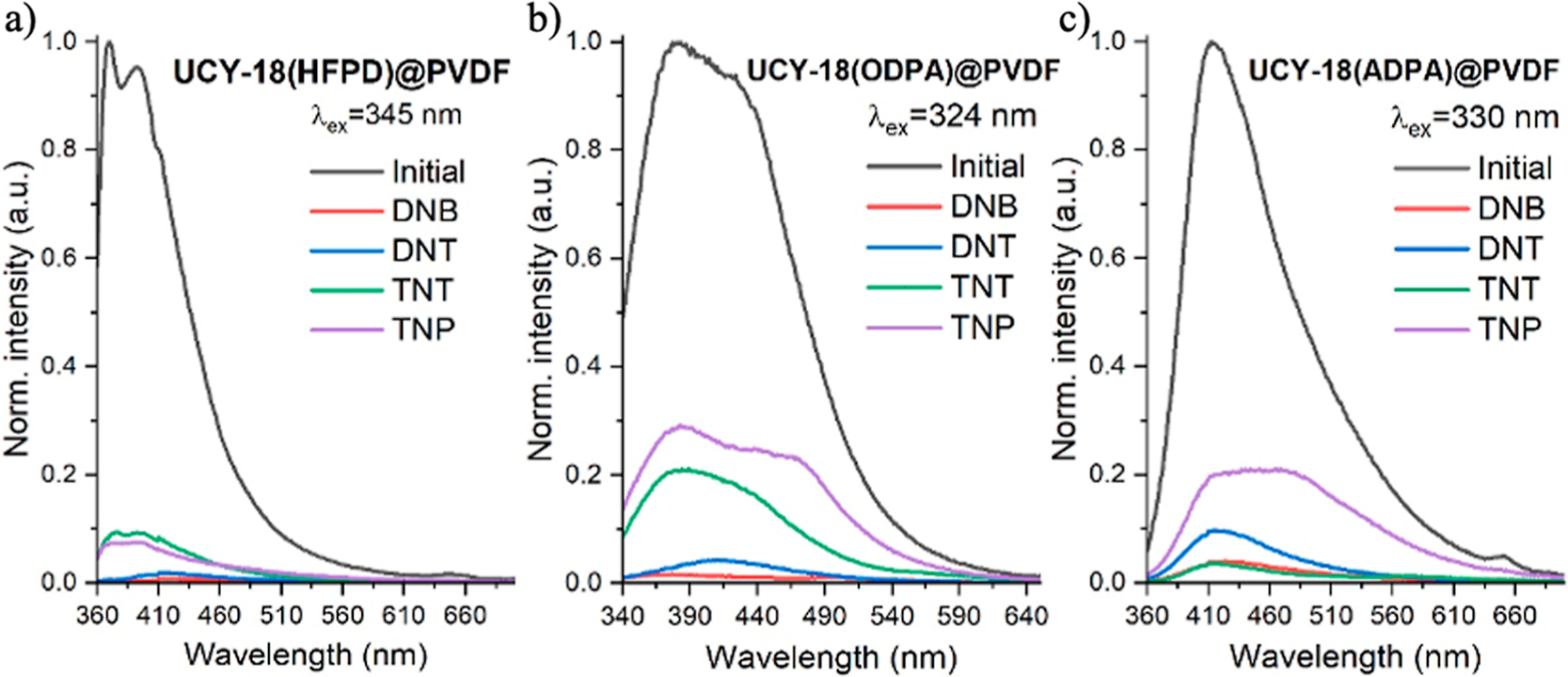
Emission spectra of (a) **UCY-18**(HFPD)@PVDF, (b) **UCY-18**(ODPA)@PVDF, and (c) **UCY-18**(ADPA)@PVDF before and after nitroaromatic gas exposure. The excitation wavelengths (λ_ex_) are indicated in the respective plots.

**Table 2:** Relative PL Change, Φ × 100 (%), of **UCY-18**(HFPD)@PVDF, **UCY-18**(ODPA)@PVDF, and **UCY-18**(ADPA)@PVDF Sensor Films and of Selected MOFs and MOF-Based Sensor Films from the Literature upon Exposure to Vapors of the Different Nitroaromatic Compounds^a^

	Φ × 100 (%)				
sample	DNB	DNT	TNT	TNP	reference
**UCY-18**(HFPD)@PVDF	98 ± 1	98 ± 1	92 ± 2	87 ± 9	this work
**UCY-18**(ODPA)@PVDF	99 ± 1	94 ± 2	74 ± 4	75 ± 7	this work
**UCY-18**(ADPA)@PVDF	97 ± 2	87 ± 10	91 ± 6	86 ± 9	this work
[Zn_2_(TCPPE)]^b^		95			^78^
**UPO-1**@PVDF/**UPO-2**@PVDF^c^			94		^79^
[Zn_2_(BPDC)_2_(BPEE)]^d^		85			^80^
[Al(OH)(BDC)_1–*n*_(L-1)]·xsolv^e^	75	60			^81^
Tb(BTC)@PMMA^f^	74	59	29	69	^41^
[Zn_1.5_(L)(H_2_O)]^g^		53			^82^
[Zn(NDC)(TED)]^h^		46			^83^
[Zn_3_(TPPE)_0.5_(TNB)_2_]^i^	21	38		40	^84^
[Zn_2_(BPDC)_2_(BPEE)]^d^		35			^85^
[Tb(L)(OH)]·xsolv^j^	20	0	0	10	^86^
[Zn(DCBPY)(DMF)]^k^		8			^87^

a The error in Φ × 100 was calculated using the standard deviation (σ) of three independent measurements.

b H_4_TCPPE = tetrakis­[4-(4-carboxyphenyl)­phenyl]­ethene.

c UPO-1 = [Zr_6_(μ_3_-O)_4_(μ_3_-OH)_4_(PEPEP-NO_2_)_6_]_*n*_; UPO-2 = [Zr_6_(μ_3_-O)_4_(μ_3_-OH)_4_(PEPyEP)_6_]_*n*_; H_2_PEPEP–NO_2_ = 4,4′-((2-nitro-1,4-phenylene)­bis­(ethyne-2,1-diyl)) dibenzoic acid; H_2_PEPyEP = 4,4′-(pyrazine-2,5-diylbis­(ethyne-2.1-diyl)) dibenzoic acid.

d H_2_BPDC = 4,4′-biphenyl dicarboxylate; BPEE = 1,2-bipyridylethene.

e H_2_BDC = terephthalic acid; H_2_L-1 = 2-((benzyl)­amino)-terephthalic acid.

f H_3_BTC = benzene-1,3,5-tricarboxylic acid; PMMA = polymethyl-methacrylate.

g H_3_L = 4,4′,4″-(benzene-1,3,5-triyltris­(oxy))­tribenzoic acid.

h H_2_NDC = 2,6-naphthalene dicarboxylic acid; TED = triethylenediamine.

i H_4_TPPE = 1,1,2,2-tetrakis­(4-(pyridine-4-yl)­phenyl)­ethane; H_3_TNB = 4,4′,4″-nitrilotrisbenzoic acid.

j H_2_L = 5-(4-carboxyphenyl)­pyridine-2-carboxylate.

k H_2_DCBPY = 2,2′-bipyridine-4,4′-dicarboxylate.

In addition, the membranes **UCY-18**(HFPD)@PVDF and **UCY-18**(ODPA)@PVDF suffered a noticeable color change in the presence of the analytes, especially when they were exposed to DNB, TNT, and TNP, as shown in Figure 6, which indicates the high sorption capacity of the membranes for the corresponding nitroaromatic molecules. On the other hand, **UCY-18**(ADPA)@PVDF did not show an appreciable color change, probably because the membrane was already intensely colored before exposure.

**Figure 6:**
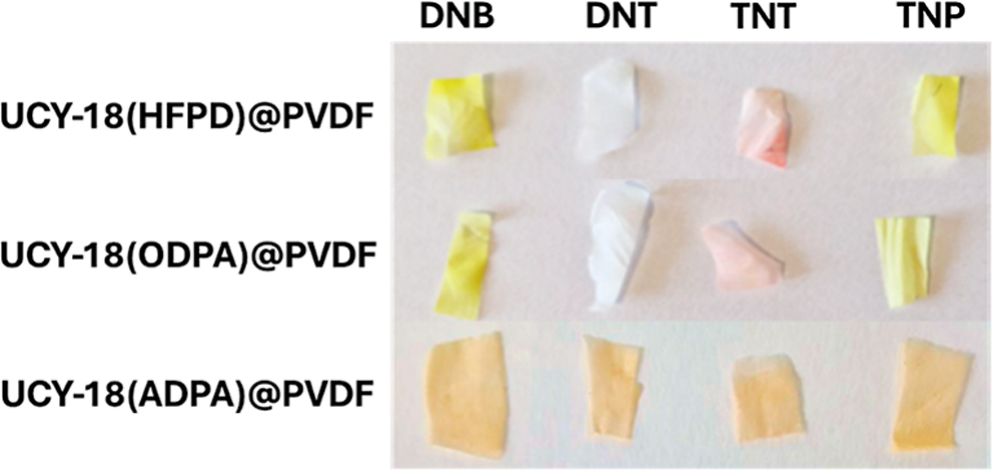
Photographs of **UCY-18**(HFPD)@PVDF, **UCY-18**(ODPA)@PVDF, and **UCY-18**(ADPA)@PVDF membranes after 48 h of exposure to DNB, DNT, TNT, and TNP.

Regarding the mechanism of the PL quenching observed for the different analytes, it can be attributed to photoinduced charge transfer from the luminophores in the MOF to the electron-withdrawing analyte molecules. These act as π-electron acceptors, producing quenching of the PL signal through the creation of an alternative nonradiative energy transfer pathway from the excited state of the ligands. This sensing mechanism has been previously demonstrated for similar MOF-based sensors^41,88,89^ and can be applied here after ruling out other possible explanations for the observed quenching, such as degradation of the emitting material, inner filter effect (IFE), or fluorescence resonance energy transfer (FRET). The crystallinity and structural integrity of the MOFs after exposure to the analytes are preserved, as shown below, and therefore, structural collapse can be excluded. Moreover, the low concentration of the analytes (see Experimental Details section) and the wavelength range of their absorption bands, which lie below 350 nm, do not allow for the IFE or FRET processes. Therefore, the quenching mechanism in this case is best explained by a photoinduced electron transfer process.

The kinetic responses of **UCY-18**(HFPD)@PVDF, **UCY-18**(ODPA)@ PVDF, and **UCY-18**(ADPA)@PVDF were also evaluated by monitoring the temporal evolution of the PL emission intensity at 425, 395, and 411 nm, respectively, upon exposure to a saturated atmosphere of the nitroaromatic compounds after 0, 1, 5, 24, and 48 h. As seen in Figure 7, the kinetic curves of the sensor responses exhibit a rapid initial change, followed by a gradual stabilization over longer times. This behavior indicates that the materials exhibit a high sensitivity toward nitroaromatic compounds. The kinetic curves also reveal that the response rate to different analytes is directly proportional to their saturation concentration. This is evidenced from the fact that analytes with higher vapor pressure, such as DNB and DNT, cause a more rapid and significant change in PL intensity. All spectral changes of each sample and analyte are depicted in Figure S56.

**Figure 7:**
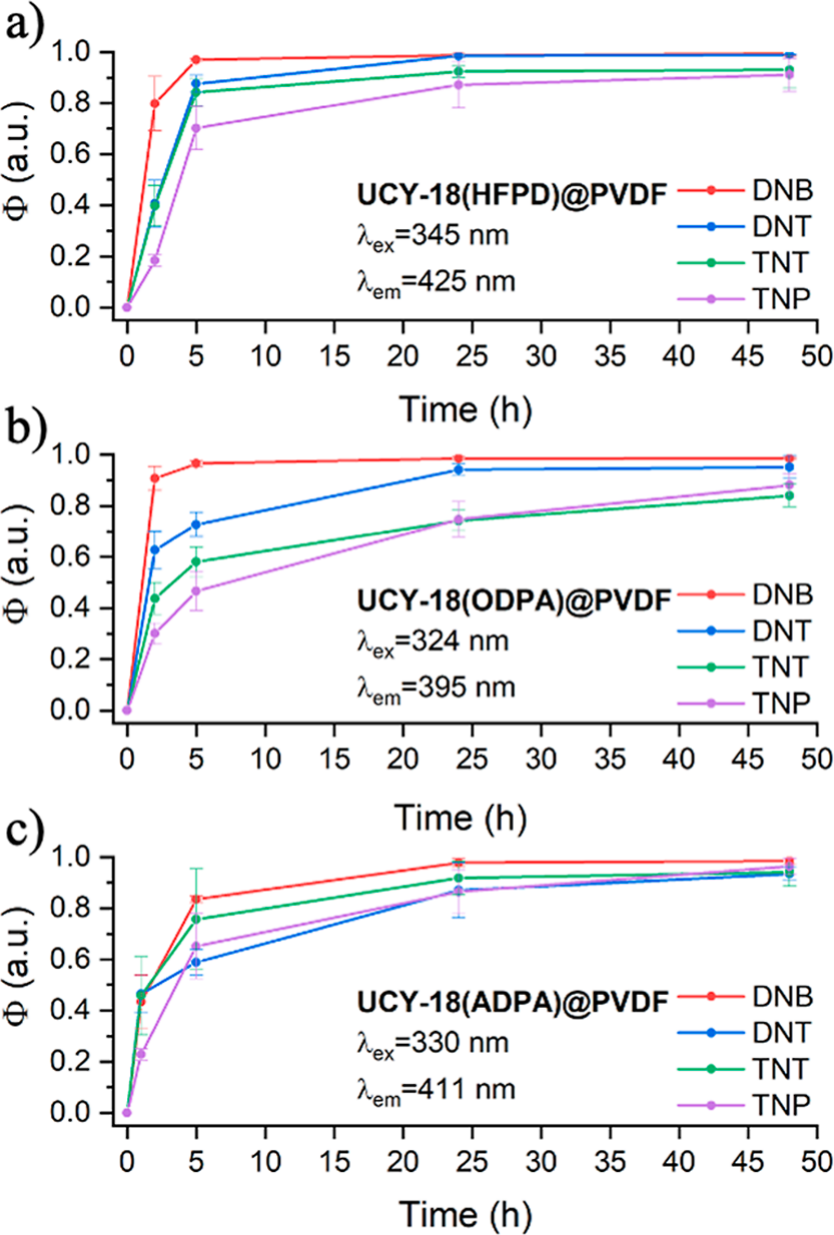
Relative PL changes (Φ) of (a) **UCY-18**(HFPD)@PVDF, (b) **UCY-18**(ODPA)@PVDF, and (c) **UCY-18**(ADPA)@PVDF after exposure to the different analytes as a function of time. The corresponding λ_ex_ and λ_em_ are indicated in the different plots.

The stability of the MOFs embedded in the membranes was investigated by μ-XRD after a 48 h exposure period. The diffractograms showed that all the materials retained their crystallinity and structural integrity after the continuous exposure to nitroaromatic vapors (Figure S57).

To evaluate the selectivity of the sensing materials, the values of PL changes (Φ) presented in Table 2 were divided by the corresponding vapor pressure (*P*_i_) of the sample. This was required because the sensing response of the materials is concentration dependent. The results of the PL changes per concentration unit (atm^–1^) are shown in Figure 8 for the different nitroaromatic explosives, also considering other possible interfering agents such as toluene, Cl–benzene, and benzoic acid. In the case of the explosive analytes, the same trend in the Φ/*P*_i_ values was obtained for the three MOFs; that is TNP > TNT > DNT ≈ DNB. This trend can be explained by the redox potential values of each molecule, which is proportional to their electron-withdrawing nature. In particular, TNP has a redox potential of −0.4 V,^90^ TNT −0.7 V,^91^ DNB −0.9 V,^91^ and DNT −1.0 V.^91^ Therefore, it is expected that TNP, with a higher redox potential, exhibits a stronger electron-withdrawing character compared to the others, and for this reason, it causes a more pronounced PL quenching response, in agreement with the photoinduced electron transfer mechanism proposed above. In the case of the interferents, we chose various benzene derivatives with different substitution of the phenyl ring, i.e., toluene, Cl–benzene, and benzoic acid, since nitroaromatic molecules also belong to this general category of compounds. After the films were exposed to saturated vapors of these compounds, lower PL changes were obtained (Figure S58) despite their much higher vapor pressures at standard conditions compared to those of the nitroaromatic compounds. This finding highlights the selectivity of the MOF@PVDF films toward the detection of nitroaromatic explosives with high oxidizing character, especially for TNP and TNT. Moreover, the PL change after exposure to the interferents involves an increase in the emission, in contrast to the situation with nitroaromatic analytes, which is attributed in this case to the π-electron donating nature of these species.

**Figure 8:**
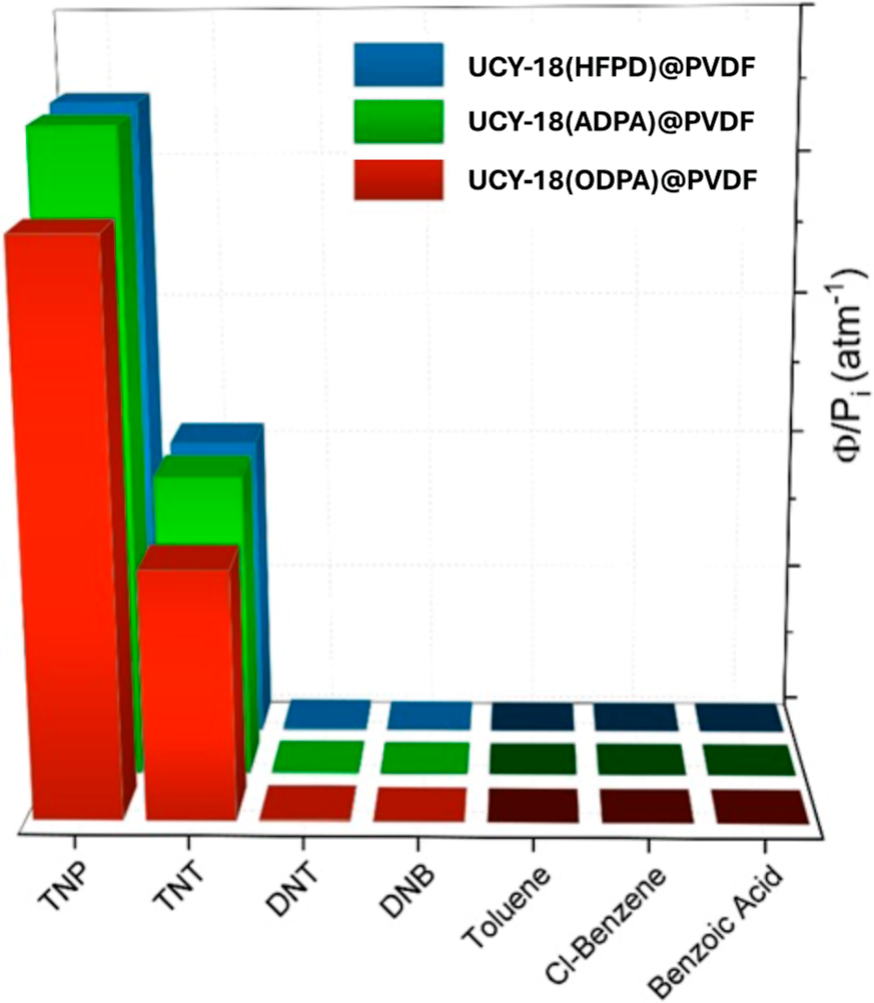
Relative PL change (Φ) per concentration unit (atm^–1^) of **UCY-18**(HFPD)@PVDF (blue bars), **UCY-18**(ADPA)@PVDF (green bars), and **UCY-18**(ODPA)@PVDF (red bars) membranes after exposure to different analytes.

### SCSC Reactions with Nitroaromatic Compounds

The determination of the crystal structures of the MOFs loaded with selected nitroaromatic compounds was targeted with priority to obtain information about the structural interactions of the nitroaromatic molecules with the framework of **UCY-18**(L) that may be responsible for luminescence quenching. These studies involved heterogeneous reactions of single crystals of **UCY-18**(HFPD) with vapors of selected nitroaromatic compounds that led to the determination of the crystal structures of **UCY-18**(HFPD)·nPhNO_2_ and **UCY-18**(HFPD)·n*o*-NO_2_Tol. The exchanged analogues retained their crystallinity and structural integrity as shown from pXRD studies (Figure S59), whereas their IR spectra provided an initial indication of the existence of the nitroaromatic molecules in their structure due to the presence of the vibrational bands at ∼1350 and 1520 cm^–1^ corresponding to the symmetrical and asymmetrical stretching of the N–O bonds that are not present in the spectrum of the pristine compound (Figure S60). This was confirmed from the determination of their crystal structures, which showed that the exchanged analogues crystallize in the tetragonal space group *I*4̅2*d* as the pristine MOF and contain one nitroaromatic molecule per SBU. In both compounds, the nitroaromatic molecules are arranged around the channels of compound **UCY-18**(HFPD) (Figure 9) and in close proximity to the framework of the MOF interacting strongly with it through hydrogen bonding interactions. These involve the oxygen atoms of the –NO_2_ group (atoms O11 and O12) and the terminally bound water molecules (atoms O9 and O10) of UCY-18­(HFPD) (hydrogen bond distances: O9···O11 2.784 Å, O10···O12 3.140 Å for **UCY-18**(HFPD)·*n*PhNO_2_ and O9···O11 2.801 Å, O10···O12 3.242 Å for **UCY-18**(HFPD)·n*o*-NO_2_Tol). The strong interactions between the nitroaromatic molecules and the framework of **UCY-18**(HFPD) may account for the luminescence quenching observed after exposure of the materials to the nitroaromatic compounds. In addition, the trifluoromethyl groups (−CF_3_) of **UCY-18**(HFPD) interact with the benzene ring of the nitroaromatic molecules in both cases through −C–F···π interactions (distances: F3···aromatic ring = 3.874 Å for **UCY-18**(HFPD)·*n*PhNO_2_ and F6···aromatic ring = 3.840 Å for **UCY-18**(HFPD)·n*o*-NO_2_Tol). The determination of the exact amount of the nitroaromatic molecule in the compounds (the crystallographic studies could not exclude the possibility of the existence of additional severely disordered molecules) was performed through ^1^H NMR studies in digested **UCY-18**(HFPD)·*n*PhNO_2_ and **UCY-18**(HFPD)·n*o*-NO_2_Tol MOFs in deuterated DMSO/DCl and thermogravimetric analysis (Figures S61–S64). These studies also confirmed the presence of nitroaromatic molecules in the structures, suggesting the presence of 2.5 equiv of nPhNO_2_ and 2 of *o*-NO_2_Tol in the exchanged analogues **UCY-18**(HFPD)·nPhNO_2_ and **UCY-18**(HFPD)·n*o*-NO_2_Tol, respectively.

**Figure 9:**
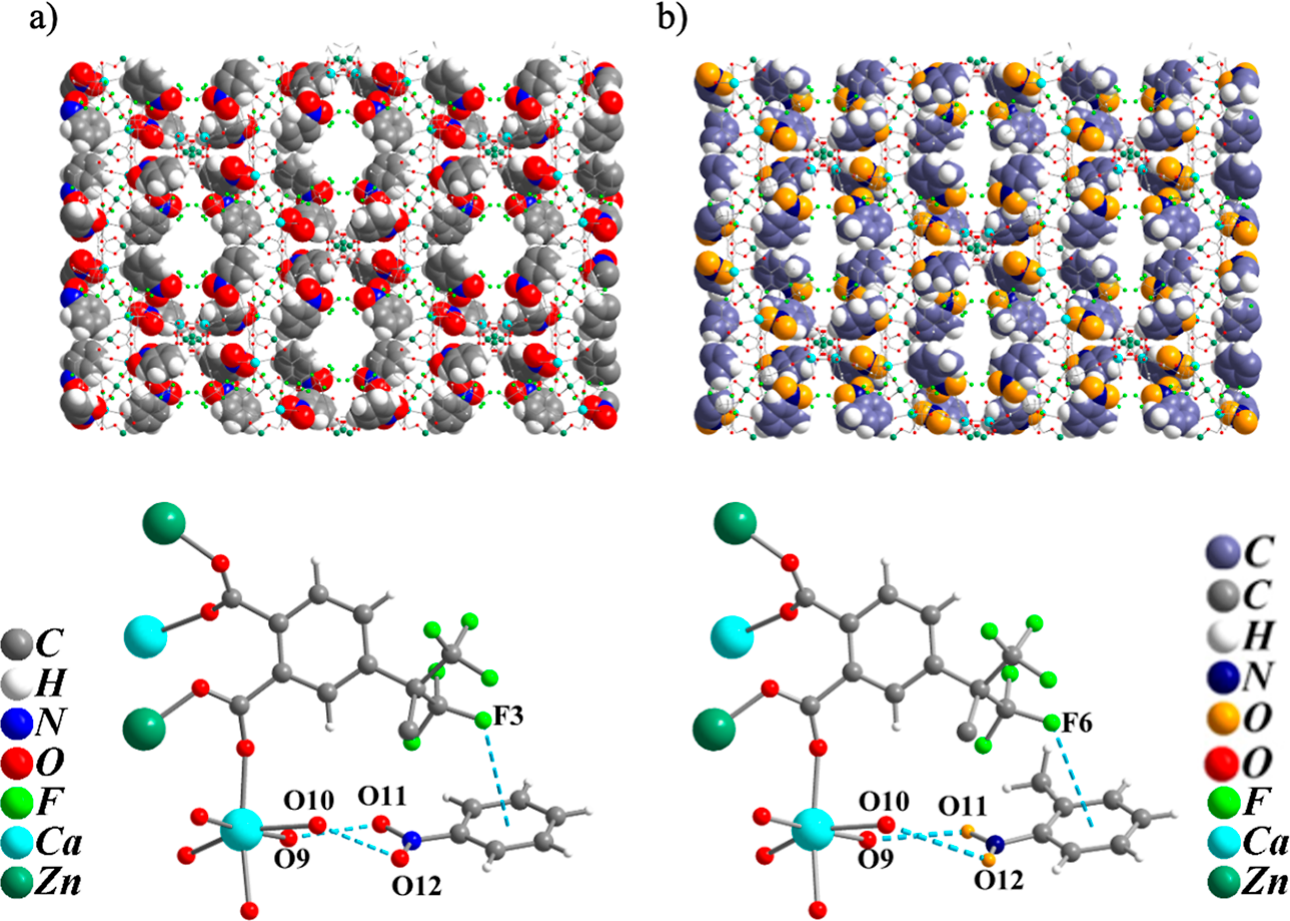
Representations of the 3-D structure (top) and part of the SBU (bottom) of (a) compound **UCY-18**(HFPD)·*n*PhNO_2_ and (b) compound **UCY-18**(HFPD)·n*o*-NO_2_Tol emphasizing on the arrangement and structural interactions of the nitroaromatic molecules with the framework of the MOFs. Color code: Zn, dark green; Ca, turquoise; F, light green; O, red-yellow; N, blue-dark blue; C, gray-gray blue; H, white.

## Conclusions

A new family of 3-dimensional heterometallic Zn/Ca-MOFs based on V-shaped angular tetracarboxylic ligands is reported with the general formulas [ZnCa­(L)­(S)­(S′)]_*n*_ (S, S′ = H_2_O and H_4_L = H_4_HFPD **UCY-18**(HFPD); S = H_2_O, S′ = DMF and H_4_L = H_4_BPTC **UCY-18**(BPTC), H_4_L = H_4_ODPA **UCY-18**(ODPA), H_4_L = H_4_ADPA **UCY-18**(ADPA)). These compounds are unique examples of mixed metal Zn/Ca-MOFs with diphthalic ligands, exhibit analogous structures with the main alteration being the different functional groups that connect the diphthalic acid groups of the ligands, and display high solvent accessible volumes ranging from 62.5% in **UCY-18**(HFPD) to 75.6% in **UCY-18**(ADPA). Gas sorption studies confirmed that **UCY-18**(L) (H_4_L = H_4_HFPD, H_4_BPTC, H_4_ODPA, H_4_ADPA) display microporous structures and exhibit significant internal surface areas of 1523, 2070, 2134, and 1338 m^2^ g^–1^, respectively. Low pressure (up to 1 bar) gas sorption studies involving gases of environmental interest (CO_2_, CH_4_, and H_2_) showed significant CO_2_ sorption capacity for selected analogues, with the higher one appearing for **UCY-18**(ADPA) (5.0 mmol g^–1^ at 273 K, 3.8 mmol g^–1^ at 283 K and 2.3 mmol g^–1^ at 298 K). In addition, vapor sorption studies using aromatic and aliphatic organic molecules demonstrated high sorption capacities for all compounds, even at relatively low relative pressures, with **UCY-18**(HFPD) in particular showing a higher affinity for aromatic molecules. Sensing studies on thin films of **UCY-18**(L) (H_4_L = H_4_HFPD, H_4_BPTC, H_4_ODPA, H_4_ADPA) embedded in PVDF, **UCY-18**(HFPD)@PVDF, **UCY-18**(ODPA)@PVDF, and **UCY-18**(ADPA)@PVDF revealed a variety of different PL responses upon exposure to saturated vapors of various nitroaromatic compounds. These MOF-based films were found to display high selectivity in detecting TNP and TNT vapors in comparison to other nitroaromatic compounds and interferents at the same concentration. Single-crystal-to-single-crystal exchange reactions of the pristine **UCY-18**(HFPD) MOF with selected nitroaromatic molecules as PhNO_2_ and *o*-NO_2_Tol were successfully performed, shedding light on the structural alterations taking place to the MOFs upon exposure to vapors of nitroaromatic compounds. These studies revealed that the nitroaromatic compounds are located close to the framework of the MOF interacting strongly with it through hydrogen bonds and also −C–F···π interactions. These structural interactions may be responsible for the facile insertion of the nitroaromatic compounds into the pores of the MOFs even at low relative pressures, the quenching of their PL signal, and as a result the sensitivity and selectivity of the reported materials. Overall, this work highlights the capability of emissive angular diphthalic ligands to stabilize heterometallic Zn/Ca MOFs with microporous structures displaying various functional groups that enabled the development of selective sensors for nitroaromatic compounds, providing a strategy to achieve superior sensing materials.

## Supplementary Material



## Notes

Crystallographic tables, pXRD patterns, IR spectra, TGA graphs and analysis, VT-pXRD patterns, ^1^H NMR spectra, structural figures, and supporting data for all compounds (PDF)
