# Supplementary material for: Heterometallic 3‑D Zn/Ca Metal–Organic Frameworks Based on V‑Shaped Angular Tetracarboxylic Ligands as Selective Fluorescence Sensors for Nitroaromatic Explosive Vapors
Source: Inorg Chem. 2025 Dec 16;64(51):25115–28. doi: 10.1021/acs.inorgchem.5c03921 (PMC12754755; doi:10.1021/acs.inorgchem.5c03921)
Supplement: Supplementary file 1 [file ic5c03921_si_001.pdf]

Supporting Information (SI)

Heterometallic 3-D Zn/Ca metal-organic frameworks based on V-shaped angular tetracarboxylic ligands as selective fluorescence sensors for nitroaromatic explosive vapors

Rafail P. Machattos[a], Nikos Panagiotou[a], Francisco G. Moscoso[b], Juan Jesús Romero Guerrero[b], Konstantinos G. Froudas[c], Pantelis N. Trikalitis[c], José M. Pedrosa[*b] Anastasios J. Tasiopoulos*[*a]*

[a]*Department of Chemistry, University of Cyprus, 1678 Nicosia, Cyprus.*

[b]*Center for Nanoscience and Sustainable Technologies (CNATS), Departamento de Sistemas Físicos, Químicos y Naturales, Universidad Pablo de Olavide, Ctra. Utrera km. 1, 41013 Seville, Spain.*

[c]*Department of Chemistry, University of Crete, Heraklion 71003, Greece*

Email: atasio@ucy.ac.cy

Email: jmpedpoy@upo.es

Table of Contents

Structural Tables and Schemes/Figures 3

Physical Measurements/Characterization of **UCY-18**(L) (H_4L= H_4HFPD, H_4BPTC, H_4ODPA, H_4ADPA) 12

Gas Sorption Measurements 26

Photoluminescence Studies 40

Thin film characterization and sensing studies 42

SCSC reactions with nitroaromatic compounds 46

References 50

Structural Tables and Schemes/Figures

TABLE S1. Selected Crystal Data for **UCY-18**(L) (H_4L= H_4HFPD, H_4BPTC, H_4ODPA and H_4ADPA).

**Compound**	**UCY-18**(HFPD)	**UCY-18**(BPTC)	**UCY-18**(ODPA)	**UCY-18**(ADPA)
Empirical formula	$C_{19}H_{10}O_{10}F_6CaZn$	$C_{20}H_{15}NO_{11}CaZn$	$C_{19}H_{15}NO_{11}CaZn$	$C_{19}H_{16}N_2O_{10}CaZn$
Formula Weight	617.72	550.78	538.77	537.79
Temperature / K	113(2)	100(2)	100(2)	149.97(17)
Wavelength / Å	0.71073	1.54184	1.54184	1.54184
Crystal system	Tetragonal	Tetragonal	Tetragonal	Tetragonal
Space group	$I\bar{4}2d$	$I\bar{4}2d$	$I\bar{4}2d$	$I\bar{4}2d$
a / Å	22.8231(3)	22.8023(2)	23.0010(3)	23.0765(3)
b / Å	22.8231(3)	22.8023(2)	23.0010(3)	23.0765(3)
c / Å	36.9412(2)	38.9052(3)	38.1886(8)	39.6028(7)
$\alpha = \beta = \gamma$ / °	90	90	90	90
Volume / $Å^3$	19242.4(8)	20228.6(2)	20203.5(7)	21089.5(7)
Z	16	16	16	16
$d_{calc.}$/ g/cm^3	0.853	0.723	0.709	0.678
Absorption Coefficient / mm^{-1}	0.666	1.812	1.808	1.722
F(000)	4928	4480	4384	4384
Reflections collected	66937	70240	41186	38496
Independent reflections	11565	9869	9774	10274
Completeness to θ = 66.999°	99.7% [R_{int} = 0.0610]	99.7% [R_{int} = 0.0297]	99.9% [R_{int} = 0.0465]	99.9% [R_{int} = 0.0620]
Data / Restrains / parameters	11565 / 0 / 338	9869 / 38 / 312	9774 / 7 / 303	10274 / 12 / 312
Goodness-of-fit	1.058	1.034	1.029	0.972
Final R Indices [I> 2σ(I)]	$R_{obs.}$ = 0.0413, $wR_{obs.}$ = 0.1045	$R_{obs.}$ = 0.0320, $wR_{obs.}$ = 0.0902	$R_{obs.}$ = 0.0466, $wR_{obs.}$ = 0.1231	$R_{obs.}$ = 0.0432, $wR_{obs.}$ = 0.1091
R indices [all data]	R_{all} = 0.0568, wR_{all} = 0.1116	R_{all} = 0.0332, wR_{all} = 0.0916	R_{all} = 0.0593, wR_{all} = 0.1330	R_{all} = 0.0532, wR_{all} = 0.1143

[a]$R = \Sigma||F_o|-|F_c|| / \Sigma|F_o|$, $wR = \{\Sigma[w(|F_o|^2 - |F_c|^2)^2] / \Sigma[w(|F_o|^4)]\}^{1/2}$ and

[b]$w = 1/[\sigma^2(F_o^2)+(mP)^2+nP]$ where $P=(F_o^2+2F_c^2)/3$ and m and n are constants

TABLE S2. Selected Crystal Data for **UCY-18**(HFPD)·nPhNO$_2$ and **UCY-18**(HFPD)·n*o*-NO$_2$Tol.

Compound	**UCY-18**(HFPD)·nPhNO$_2$	**UCY-18**(HFPD)·n*o*-NO$_2$Tol
Empirical formula	$C_{25}H_{15}NO_{12}F_6CaZn$	$C_{26}H_{17}NO_{12}F_6CaZn$
Formula Weight	740.83	754.85
Temperature / K	179.99(18)	179.99(18)
Wavelength / Å	1.54184	1.54184
Crystal system	Tetragonal	Tetragonal
Space group	$I\bar{4}2d$	$I\bar{4}2d$
a / Å	22.7885(2)	22.8013(2)
b / Å	22.7885(2)	22.8013(2)
c / Å	36.8103(4)	37.1390(3)
α = β = γ / °	90	90
Volume / Å^3	19116.2(3)	19308.5(2)
Z	16	16
$d_{calc.}$/ g/cm^3	1.030	1.039
Absorption Coefficient / mm^{-1}	2.231	2.216
F(000)	5952	6080
Reflections collected	133367	55534
Independent reflections	10118	8593
Completeness to θ = 66.999°	100% [R_{int} = 0.0597]	100% [R_{int} = 0.0532]
Data / Restrains / parameters	10118 / 63 / 407	8593 / 109 / 416
Goodness-of-fit	1.067	1.062
Final R Indices [I> 2σ(I)]	$R_{obs.}$ = 0.0511, $wR_{obs.}$ = 0.1491	$R_{obs.}$ = 0.0435, $wR_{obs.}$ = 0.1256
R indices [all data]	R_{all} = 0.0546, wR_{all} = 0.1530	R_{all} = 0.0473, wR_{all} = 0.1284

$^aR = \Sigma||F_o|-|F_c|| / \Sigma|F_o|$, $wR = \{\Sigma[w(|F_o|^2 - |F_c|^2)^2] / \Sigma[w(|F_o|^4)]\}^{1/2}$ and

$^bw = 1/[\sigma^2(F_o^2)+(mP)^2+nP]$ where $P=(F_o^2+2F_c^2)/3$ and m and n are constants

Table S3. Selected bond lengths of compound **UCY-18**(HFPD)

Atoms 1	Atom 2	Distance (Å)
Zn1	O2	1.918(3)
Zn1	O2[f]	1.918(3)
Zn1	O5[d]	1.929(3)
Zn1	O5[e]	1.929(3)
Zn2	O3[b]	2.001(3)
Zn2	O3[c]	2.001(3)
Zn2	O7	1.964(3)
Zn2	O7[a]	1.964(3)
Ca1	O1[f]	2.300(3)
Ca1	O4	2.221(4)
Ca1	O6[e]	2.329(3)
Ca1	O8[g]	2.317(3)
Ca1	O9	2.458(5)
Ca1	O10	2.442(6)

[a]1-X,1-Y,+Z; [b]+Y,1/2+X,1/4+Z; [c]1-Y,1/2-X,1/4+Z; [d]1-Y,+X,1-Z; [e]-1/2+Y,+X,-1/4+Z; [f]1/2-X,+Y,3/4-Z; [g]1/2-Y,1-X,-1/4+Z

Table S4. Selected bond lengths of compound **UCY-18**(BPTC)

Atoms 1	Atom 2	Distance (Å)
Zn1	O4[d]	1.943(2)
Zn1	O4	1.944(2)
Zn1	O9[e]	1.971(2)
Zn1	O9[f]	1.971(2)
Zn2	O2[a]	1.951(2)
Zn2	O2[b]	1.951(2)
Zn2	O7[c]	1.947(2)
Zn2	O7	1.947(2)
Ca1	O3[d]	2.296(2)
Ca1	O5	2.283(2)
Ca1	O6[a]	2.288(2)
Ca1	O8[e]	2.281(2)
Ca1	O10	2.335(4)
Ca1	O11	2.321(3)

[a]+Y,1-X,1-Z; [b]3/2-Y,1-X,-1/4+Z; [c]3/2-X,+Y,3/4-Z; [d]1-X,1-Y,+Z; [e]+Y,-1/2+X,1/4+Z; [f]1-Y ,3/2-X,1/4+Z

Table S5. Selected bond lengths of compound **UCY-18**(ODPA)

Atoms 1	Atom 2	Distance (Å)
Zn1	O6[a]	1.941(3)
Zn1	O6	1.941(3)
Zn1	O3[b]	1.959(4)
Zn1	O3[c]	1.959(4)
Zn2	O8	1.945(4)
Zn2	O8[d]	1.945(4)
Zn2	O5[b]	1.973(3)
Zn2	O5[e]	1.973(3)
Ca1	O2[f]	2.310(3)
Ca1	O7[g]	2.301(4)
Ca1	O9[h]	2.282(3)
Ca1	O4	2.279(4)
Ca1	O10	2.361(5)
Ca1	O11	2.337(5)

[a]1/2-X,+Y,3/4-Z; [b]-1/2+Y,+X,-1/4+Z; [c]1-Y,+X,-1+Z; [d]1-X,1-Y,+Z; [e]3/2-Y,1-X,-1/4+Z; [f]+X,3/2-Y,5/4-Z; [g]+Y,1/2+X,1/4+Z; [h]1-Y,3/2-X,1/4+Z

Table S6. Selected bond lengths of compound **UCY-18**(ADPA)

Atoms 1	Atom 2	Distance (Å)
Zn1	O3	1.954(3)
Zn1	O3[f]	1.954(3)
Zn1	O8[d]	1.983(3)
Zn1	O8[e]	1.983(3)
Zn2	O1[a]	1.976(3)
Zn2	O1[b]	1.976(3)
Zn2	O6	1.956(3)
Zn2	O6[c]	1.956(3)
Ca1	O5[a]	2.305(3)
Ca1	O2[f]	2.294(3)
Ca1	O4	2.260(3)
Ca1	O7[e]	2.253(3)
Ca1	O10	2.350(4)
Ca1	O9B	2.260(2)
Ca1	O9A	2.455(6)

[a]1-Y,+X,1-Z; [b]1-Y,1/2-X,1/4+Z; [c]+X,1/2-Y,5/4-Z; [d]1/2-Y,1-X,-1/4+Z; [e]1/2+Y,+X,-1/4 +Z; [f]1-X,1-Y,+Z

Table S7. Structural comparison of the reported **UCY-18**(L) (H_4L= H_4HFPD, H_4BPTC, H_4ODPA, H_4ADPA) MOFs with known heterometallic Zn/Ca MOFs

CCDC entry	SBU	Ligand(s)	Ligand coordination mode	Dimens-ionality	BET ($m^2 g^{-1}$)	REF.
**UCY-18**(HFPD)	$[ZnCa(COO^-)_4]_n$ Linear 1-D helical chain	$HFPD^{4-}$ [a] (angular tetracarboxylic diphthalic)	$\eta^1:\eta^1:\eta^1:\eta^1:\eta^1:\eta^1:\eta^1:\eta^1:\mu_8$	3-D	1534	This work
**UCY-18**(BPTC)	$[ZnCa(COO^-)_4]_n$ Linear 1-D helical chain	$BPTC^{4-}$ [b] (angular tetracarboxylic diphthalic)	$\eta^1:\eta^1:\eta^1:\eta^1:\eta^1:\eta^1:\eta^1:\eta^1:\mu_8$	3-D	2070	This work
**UCY-18**(ODPA)	$[ZnCa(COO^-)_4]_n$ Linear 1-D helical chain	$ODPA^{4-}$ [c] (angular tetracarboxylic diphthalic)	$\eta^1:\eta^1:\eta^1:\eta^1:\eta^1:\eta^1:\eta^1:\eta^1:\mu_8$	3-D	2134	This work
**UCY-18**(ADPA)	$[ZnCa(COO^-)_4]_n$ Linear 1-D helical chain	$ADPA^{4-}$ [d] (angular tetracarboxylic diphthalic)	$\eta^1:\eta^1:\eta^1:\eta^1:\eta^1:\eta^1:\eta^1:\eta^1:\mu_8$	3-D	1338	This work
BUVFAC	$[Zn_2Ca(COO^-)_8]_n$ Linear trinuclear trigonal prism	L^{4-} [e] (angular tetracarboxylic diisophthalic)	$\eta^1:\eta^1:\eta^1:\eta^1:\eta^0:\eta^1:\eta^1:\eta^0:\mu_5$	3-D	-	1
FUHMOM	$[Zn_2Ca_2(\mu_2\text{-}COO^-)_8]_n$ Tetranuclear	$DCPPA^{4-}$ [f] (angular tetracarboxylic isophtalic/phthalic)	$\eta^1:\eta^1:\eta^1:\eta^1:\eta^1:\eta^1:\eta^2:\eta^1:\mu_8$	3-D	-	2
FUHMUS	$[Zn_2Ca_2(\mu_3\text{-}COO^-)_2(\mu_3\text{-}COO^-)_4(COO^-)_2]_n$ Tetranuclear	$DCPPA^{4-}$ [f] (angular tetracarboxylic isophtalic/phthalic)	$\eta^1:\eta^1:\eta^1:\eta^1:\eta^1:\eta^1:\eta^2:\eta^0:\mu_8$	3-D	-	2
EJUMAA	$[ZnCa(CO_2)_6(H_2O)]_n$ Paddle-wheel binuclear	TDP^{6-} [g] (planar hexacarboxylic triisophthalic)	$\eta^1:\eta^1:\eta^0:\eta^1:\eta^1:\eta^0:\eta^1:\eta^1:\eta^1:\eta^1:\eta^0:\eta^1:\mu_8$	3-D	1611	3
FUJNAB	$[Zn_4(\mu_3\text{-}OH)_2]_n$ Tetranuclear (Ca-O-Ca-O-Zn-O-)$_2$ 12-membered ring	$IMDC^{2-}$ [h] (imidazole phthalic dicarboxylic)	$\eta^1:\eta^2:\eta^2:\eta^2:\eta^2:\eta^1:\mu_5$	3-D	-	4
FUJNEF	$[Zn_2(CO_2)]_n$ 1-D zigzag chain (Ca-COO$^-$-Zn-N-C-N-Zn-O-) rectangle-like 20-membered ring	$IMDC^{2-}$ [h] (imidazole phthalic dicarboxylic)	$\eta^1:\eta^1:\eta^1:\eta^2:\eta^2:\eta^1:\mu_4$ $\eta^1:\eta^1:\eta^1:\eta^1:\eta^1:\eta^1:\mu_3$	3-D	-	4
FUJNIJ	(Ca-OCO$^-$-Zn-OCO$^-$-Ca-OCO$^-$-Zn-OCO$^-$-) 16-membered ring	$IMDC^{2-}$ [h] (imidazole phthalic dicarboxylic)	$\eta^1:\eta^1:\eta^1:\eta^1:\eta^1:\eta^0:\mu_3$ $\eta^1:\eta^1:\eta^1:\eta^2:\eta^2:\eta^0:\mu_4$	2-D	-	4
GAXVUY	$[ZnCa(\mu_2\text{-}COO^-)_3]_n$ Dinuclear	$PBDC^{2-}$ [i] (linear dicarboxylic)	$\eta^1:\eta^1:\eta^1:\eta^1:\mu_4$	3-D	-	5
KILHEV	$[ZnCa(\mu_2\text{-}COO^-)_2]_n$ Dinuclear	1,4-NDC^{2-} [j] (linear dicarboxylic)	$\eta^1:\eta^1:\eta^1:\eta^1:\mu_4$	3-D	-	6
NOHSOU	$[Zn_2Ca(COO^-)_6)]_n$ Linear trinuclear	BTB^{3-} [k] (planar tricarboxylic)	$\eta^1:\eta^1:\eta^1:\eta^1:\eta^1:\eta^1:\mu_6$	2-D	1560	7
OSOYUR	$[Zn_2Ca(CO_2)_6(H_2O)_2]_n$ Linear trinuclear	BTC^{3-} [l] (planar tricarboxylic)	$\eta^1:\eta^1:\eta^1:\eta^1:\eta^1:\eta^1:\mu_6$	3-D	730	8
PADCII	$[Zn_2Ca(HCOO)_2(CO_2)_4]_n$ Linear trinuclear	L^{2-} [m] (dicarboxylic isophthalic) BPE [n] (Linear dipyridine)	$\eta^1:\eta^1:\eta^1:\eta^1:\mu_4$ $\eta^1:\eta^1:\mu_2$ $\eta^1:\eta^1:\mu_2$	3-D	-	9

QISFEF	$[Zn_2Ca(CO_2)_6]_n$ Linear trinuclear	MIP^{2-} [o] (dicarboxylic isophthalic) BPE [n] (Linear dipyridine)	$\eta^1:\eta^1:\eta^1:\eta^1:\mu_4$ $\eta^1:\eta^1:\mu_2$ $\eta^1:\eta^1:\mu_2$	3-D	-	10
REFVAB	$[Zn_2Ca(COO^-)_6)]_n$ Linear trinuclear	$TBIP^{2-}$ [p] (dicarboxylic isophthalic)	$\eta^1:\eta^1:\eta^1:\eta^1:\mu_4$	3-D	-	11
REFTUT	$[Zn_2Ca(COO^-)_6)]_n$ Linear trinuclear	MIP^{2-} [o] (dicarboxylic isophthalic)	$\eta^1:\eta^1:\eta^1:\eta^1:\mu_4$	3-D	-	11
XABRAW	$[ZnCa(COO^-)_4)]_n$ Dinuclear	$BTEC^{4-}$ [q] (tetracarboxylic diphthalic)	$\eta^1:\eta^1:\eta^1:\eta^1:\eta^1:\eta^1:$ $\eta^1:\eta^1:\mu_8$	3-D	-	12

[a]**H_4**HFPD = 4,4′-hexafluoroisopropilidene diphthalic acid; [b]**H_4**BPTC = 3,3′,4,4′-benzophenone tetracarboxylic acid; [c]**H_4**ODPA = 4,4′-oxydiphthalic acid; [d]**H_4**ADPA = 4,4′-azanediyl diphthalic acid; [e]**H_4**L = 5,5′-(propane-1,3-diyl)-bis (oxy)diisophthalic acid; [f]**H_4**DCPPA = 3-(3′,5′-dicarboxyphenoxy)phthalic acid; [g]**H_6**TDP = 2,4,6-tri(2,4-dicarboxy phenyl)pyridine; [h]**H_2**IMDC = 4,5-imidazoledicarboxylic acid; [i]**H_2**PBDC = terephthalic acid; [j]1,4-**H_2**NDC = 1,4-naphthalene dicarboxylic acid; [k]**H_3**BTB = benzene-1,3,5-tribenzoate; [l]**H_3**BTC = benzene-1,3,5-tricarboxylic acid; [m]**H_2**L = 5-methoxyisophthalic acid; [n]BPE = 1,2-bi(4-pyridyl)ethene; [o]**H_2**MIP = 5-methyl isophthalic acid; [p]**H_2**TBIP = 5-tertbutyl isophthalic acid; [q]**H_4**BTEC = 1,2,4,5-benzenetetracarboxylic acid.

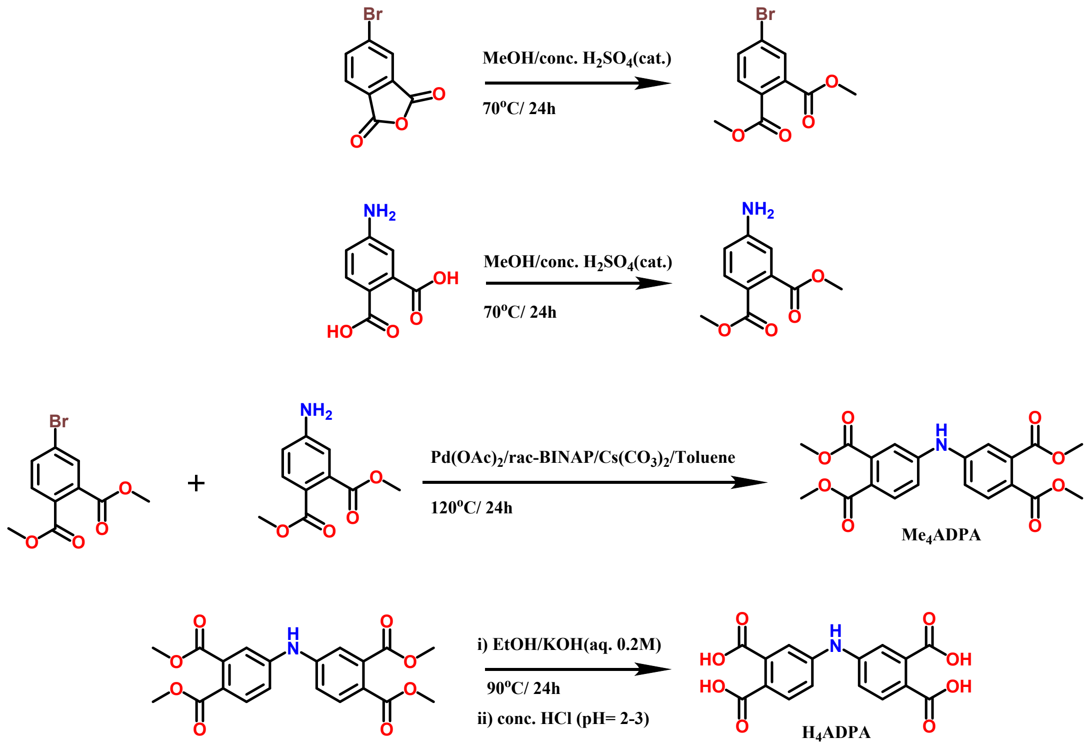

Scheme S1. Schematic representation of the synthetic route that led to the formation of H_4ADPA ligand. The ligand was synthesized by following a similar procedure to the one employed for the isolation of 4,4′-dicarboxyphenylamine[13],with differences in the starting materials.

a)

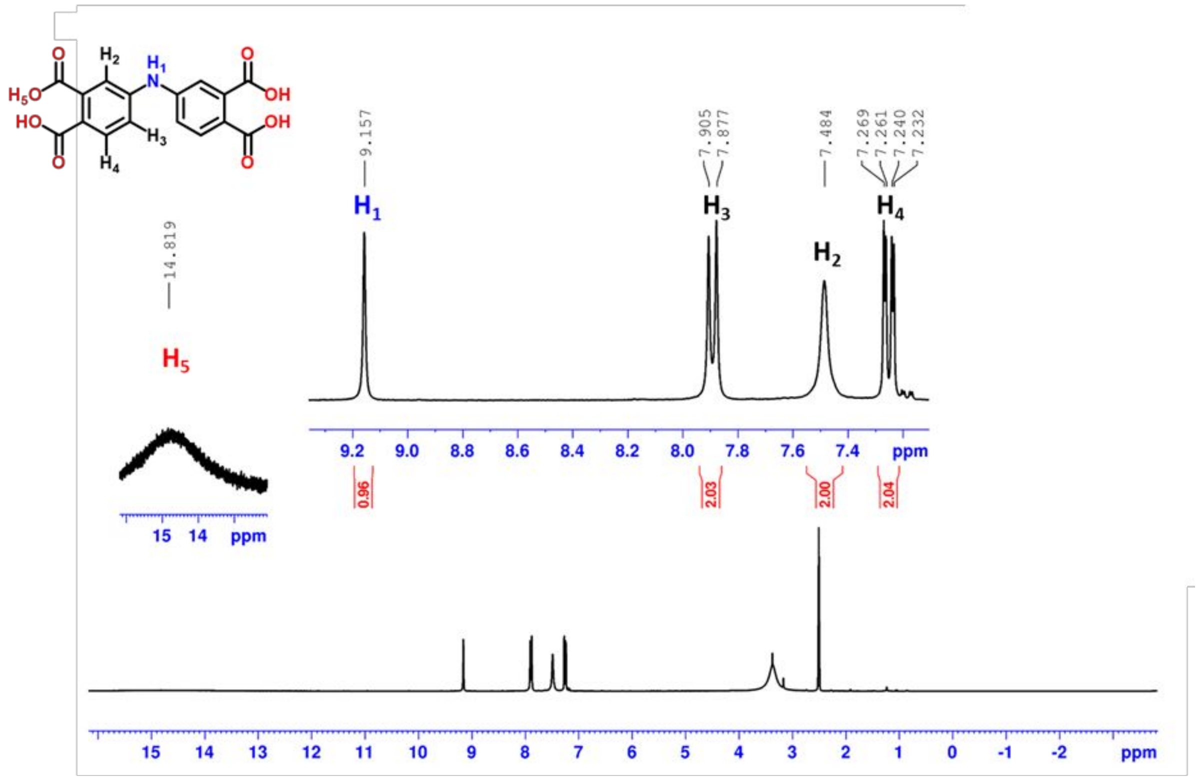

b)

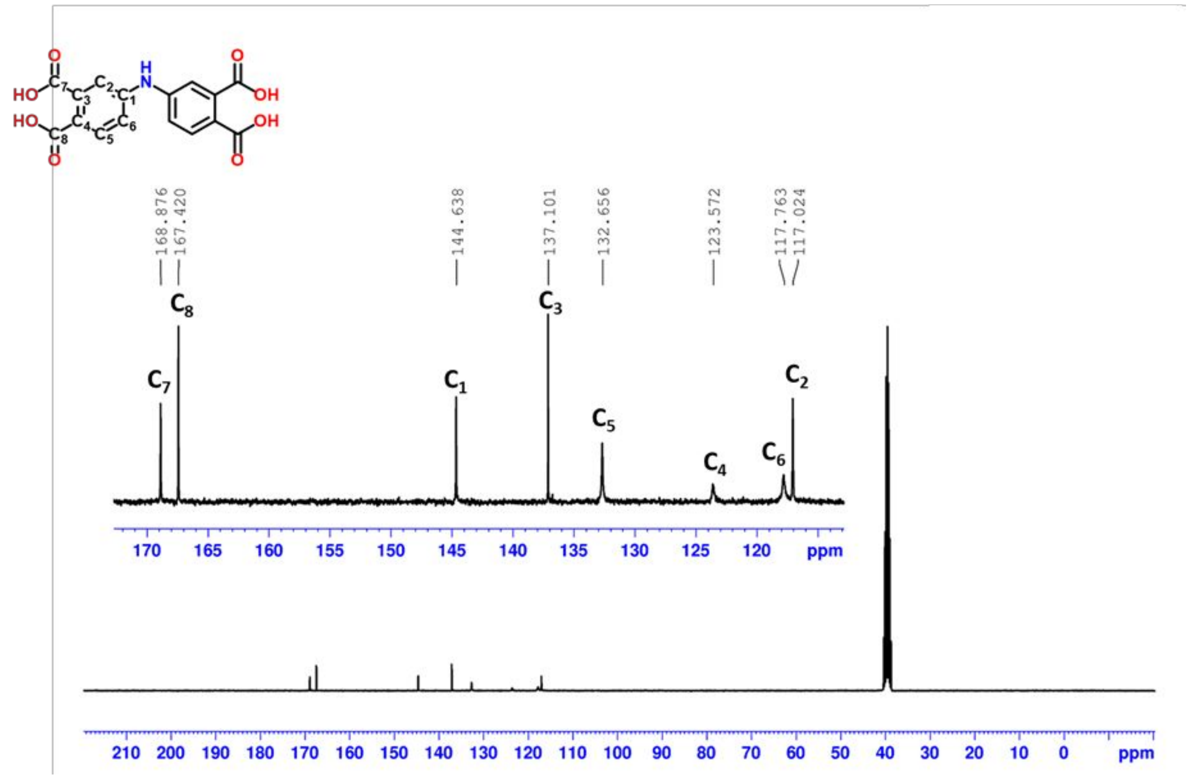

Scheme S2. a) ^{1}H-NMR and b) ^{13}C-NMR spectra of H_4ADPA ligand.

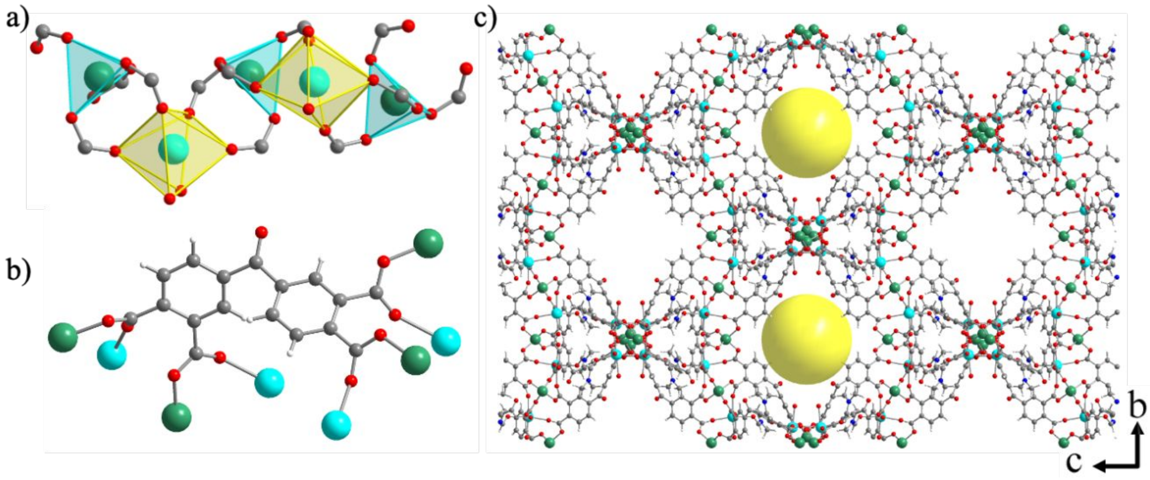

Figure S1. Representations of the: a) 1-D helical chain $[ZnCa(COO)_4]$ SBU, b) the $\eta^1:\eta^1:\eta^1:\eta^1:\eta^1:\eta^1:\eta^1:\eta^1:\mu_8$ coordination mode of the crystallographically independent $BPTC^{4-}$ ligand and c) packing of the 3D structure along the a-axis emphasizing on the intralayer tetragonal channels of compound **UCY-18**(BPTC). The yellow spheres (shown in c) denote the pores formed along a-axis. Colour code: Zn, dark green; Ca, turquoise; O, red; N, blue; C, grey; H, white.

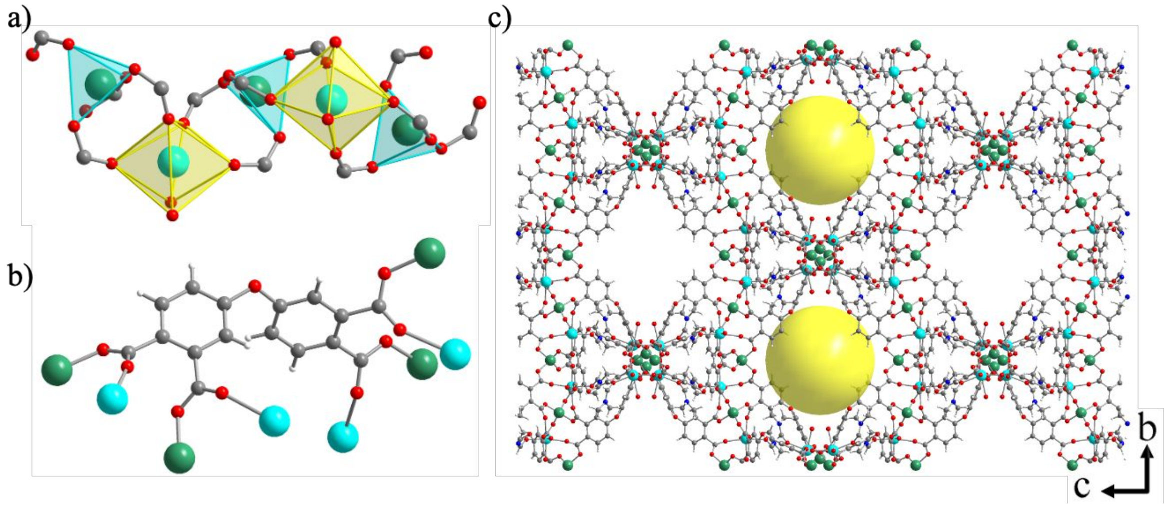

Figure S2. Representations of the: a) 1-D helical chain $[ZnCa(COO)_4]$ SBU, b) the $\eta^1:\eta^1:\eta^1:\eta^1:\eta^1:\eta^1:\eta^1:\eta^1:\mu_8$ coordination mode of the crystallographically independent $ODPA^{4-}$ ligand and c) packing of the 3D structure along the a-axis emphasizing on the intralayer tetragonal channels of compound **UCY-18**(ODPA). The yellow spheres (shown in c) denote the pores formed along a-axis. Colour code: Zn, dark green; Ca, turquoise; O, red; N, blue; C, grey; H, white.

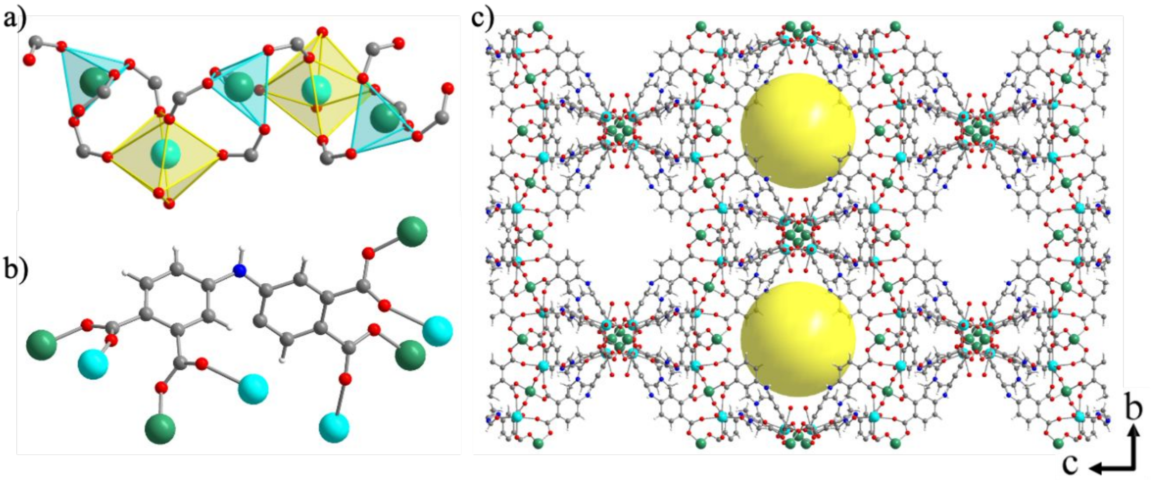

Figure S3. Representations of the: a) 1-D helical chain $[ZnCa(COO)_4]$ SBU, b) the $\eta^1:\eta^1:\eta^1:\eta^1:\eta^1:\eta^1:\eta^1:\eta^1:\mu_8$ coordination mode of the crystallographically independent $ADPA^{4-}$ ligand and c) packing of the 3D structure along the a-axis emphasizing on the intralayer tetragonal channels of compound **UCY-18**(ADPA). The yellow spheres (shown in c) denote the pores formed along a-axis. Colour code: Zn, dark—[green; Ca, turquoise; O, red; N, blue; C, grey; H, white.

Physical Measurements/Characterization of UCY-18(L) (H_4L= H_4HFPD, H_4BPTC, H_4ODPA, H_4ADPA)

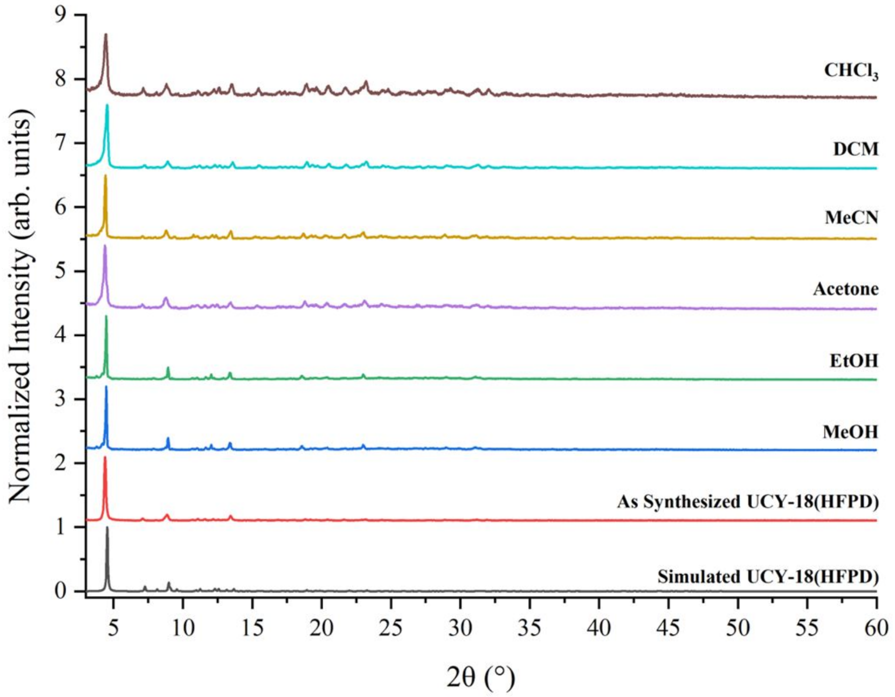

Figure S4. Powder X-ray diffraction patterns of **UCY-18**(HFPD) MOF treated in the indicated organic solvents.

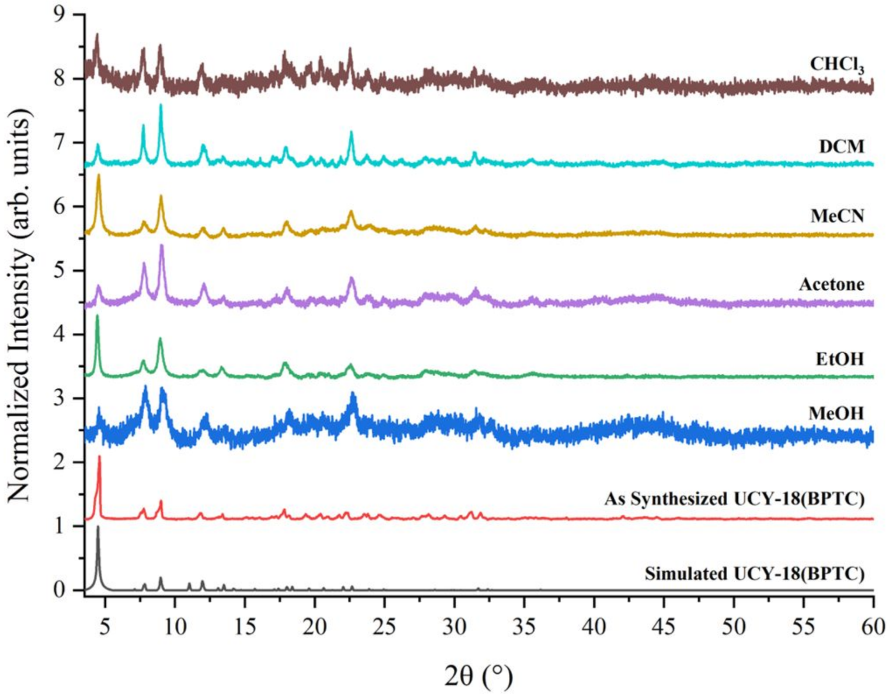

Figure S5. Powder X-ray diffraction patterns of **UCY-18**(BPTC) MOF treated in the indicated organic solvents.

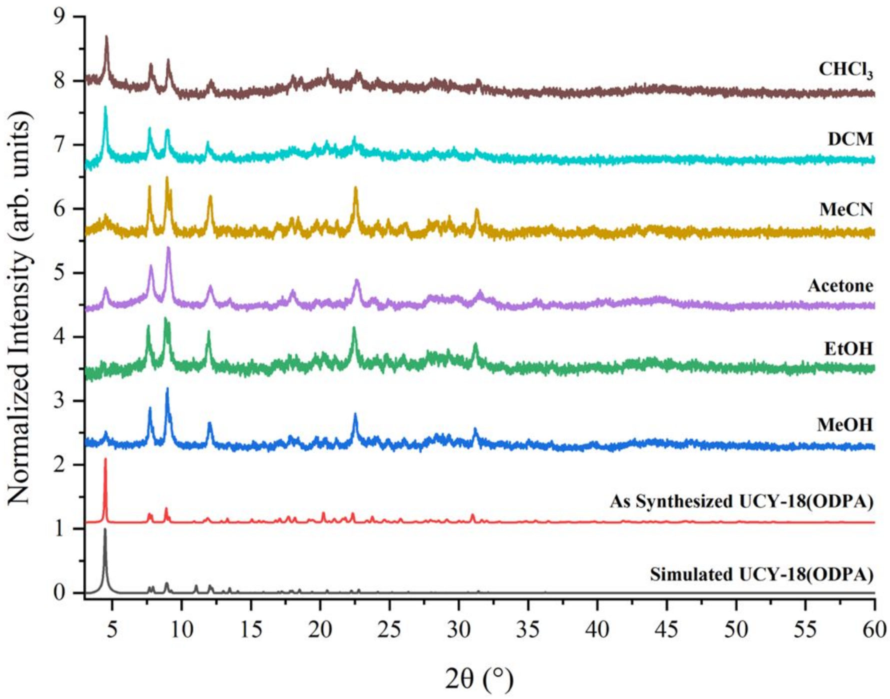

Figure S6. Powder X-ray diffraction patterns of **UCY-18**(ODPA) MOF treated in the indicated organic solvents.

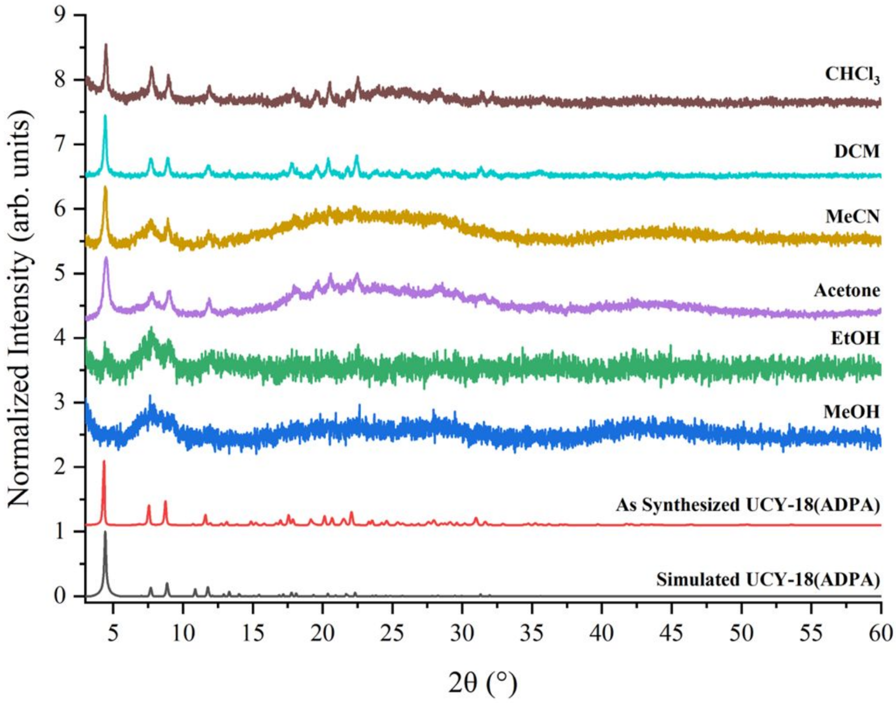

Figure S7. Powder X-ray diffraction patterns of **UCY-18**(ADPA) MOF treated in the indicated organic solvents.

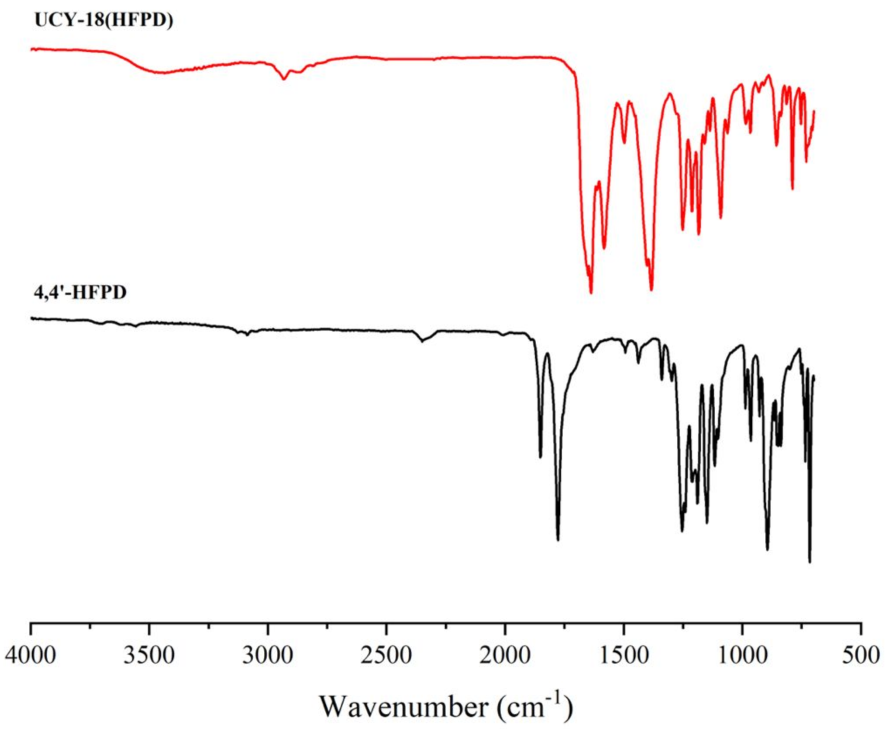

Figure S8. IR spectra of 4,4′-HFPD ligand and the as synthesized **UCY-18**(HFPD) MOF.

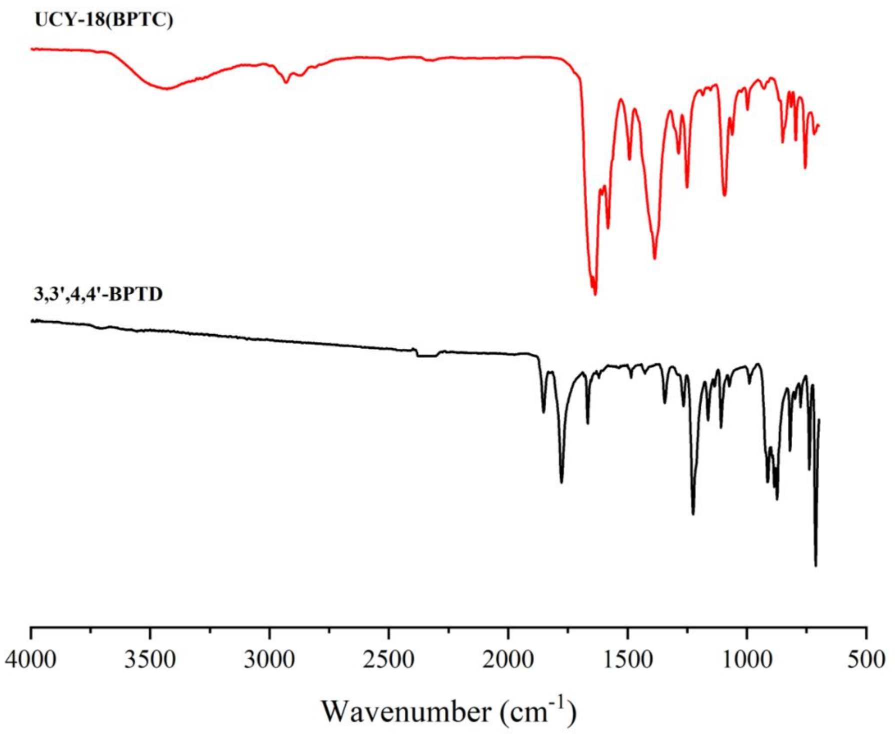

Figure S9. IR spectra of 3,3′,4,4′-BPTD ligand and the as synthesized **UCY-18**(BPTC) MOF.

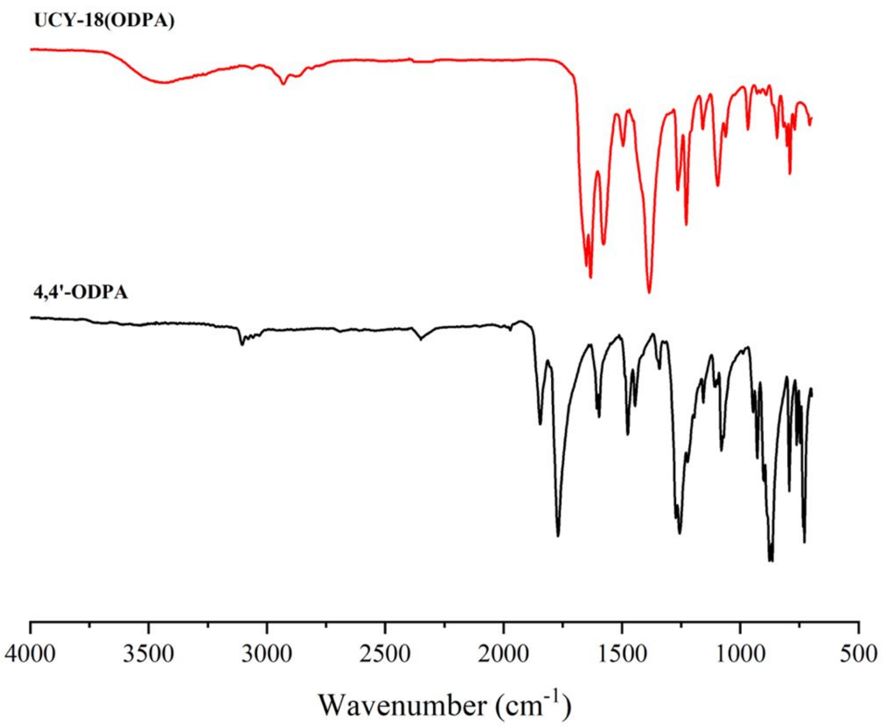

Figure S10. IR spectra of 4,4′-ODPA ligand and the as synthesized **UCY-18**(ODPA) MOF.

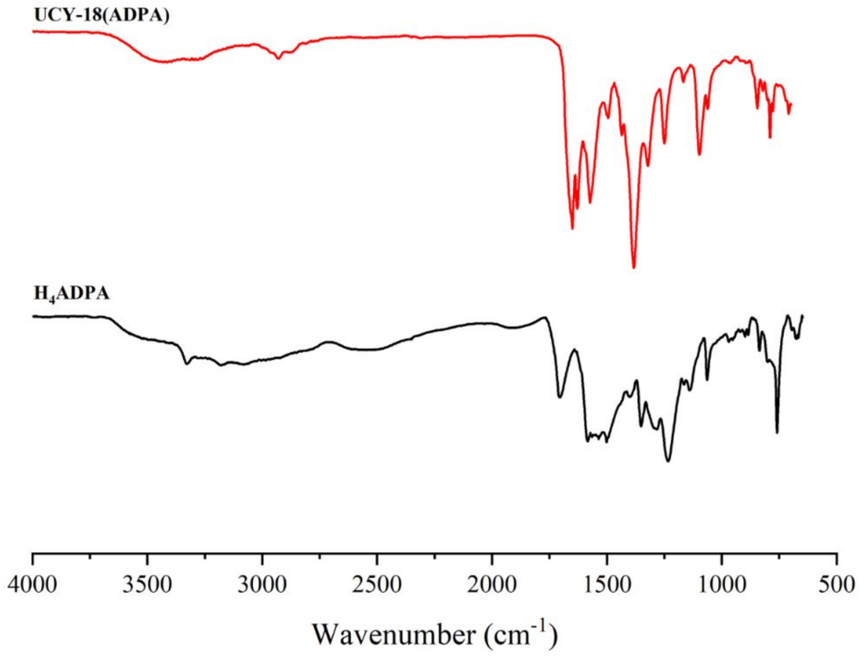

Figure S11. IR spectra of H_4ADPA ligand and the as synthesized **UCY-18**(ADPA) MOF.

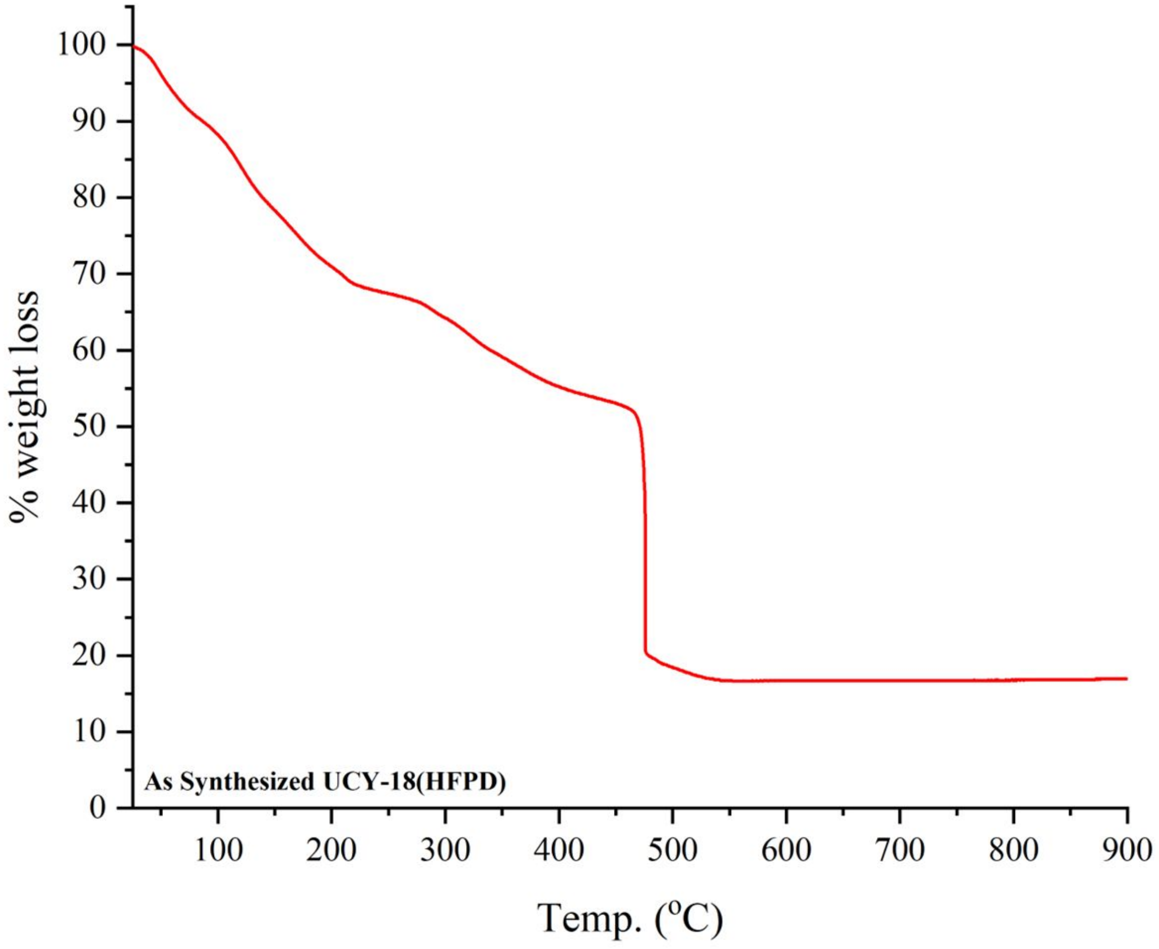

Figure S12. TGA graph of the as synthesized compound **UCY-18**(HFPD).

The TGA curve of **UCY-18**(HFPD) revealed that the thermal decomposition of this compound proceeds via a multi-step process. A rough analysis of the TGA plot shall be provided below however, there is uncertainty in this because the curve is quite complicated containing several different mass losses (guest DMF and terminal H_2O molecules and organic ligand) some of which happen in the same temperature range as evidenced from the lack of plateaus in the curve. The release of guest and coordinated solvent molecules (DMF + H_2O) involves continuous mass losses up to 350 °C whereas the last mass loss at higher temperatures is due to the decomposition of the organic ligand $HFPD^{4-}$. In particular, the mass losses in the temperature range 25 °C to 350 °C correspond to ~40.9 % of the material's total mass and are attributed to the removal of two terminally ligated H_2O and 5 guest DMF molecules (calc. on the basis of the formula $[ZnCa(HFPD)(H_2O)_2]_n$·5nDMF (**UCY-18**(HFPD)·5nDMF 40.8%). The second mass loss, assigned to the decomposition of the organic ligand $HFPD^{4-}$, occurring in the ~ 350 – 550 °C region corresponds to 45.3% (calc. for **UCY-18**(HFPD)·5nDMF 45.2%) of the material's total mass. Lastly, the residual mass (13.8%) at 900 °C corresponds to ZnO + CaO (calc. for **UCY-18**(HFPD)·5nDMF 14.0%).

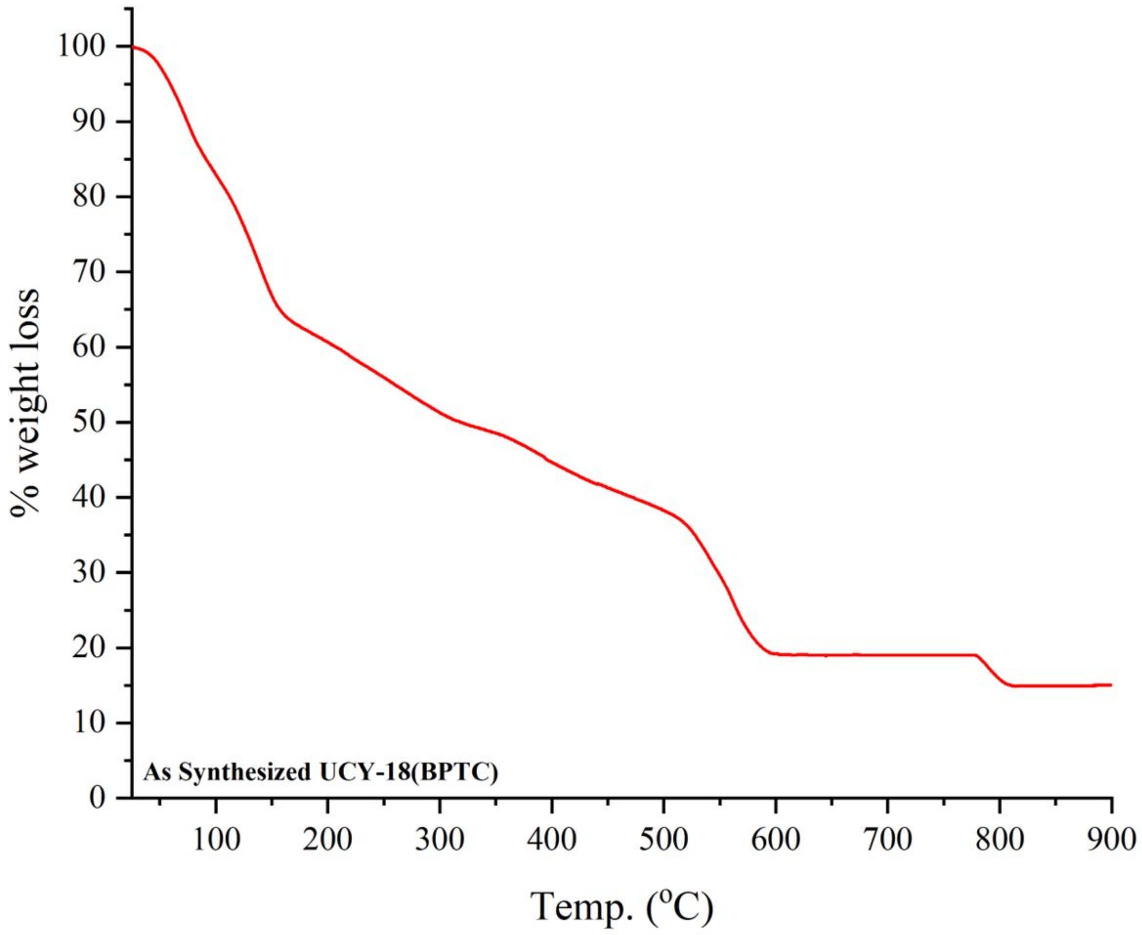

Figure S13. TGA graph of the as synthesized compound **UCY-18**(BPTC).

The TGA curve of **UCY-18**(BPTC) revealed that the thermal decomposition of this compound proceeds via a multi-step process. A rough analysis of the TGA plot shall be provided below however, there is uncertainty in this because the curve is quite complicated containing several different mass losses (guest / terminal DMF and terminal H_2O molecules and organic ligand) some of which happen in the same temperature range as evidenced from the lack of plateaus in the curve. The release of guest and coordinated solvent molecules (DMF + H_2O) involves continuous mass losses up to 350 °C whereas the last mass loss at higher temperatures is due to the decomposition of the organic ligand $BPTC^{4-}$. In particular, the mass losses in the temperature range 25 °C to 350 °C correspond to ~49.6 % of the material's total mass and are attributed the removal of terminally ligated (one DMF and one H_2O) and guest (5 DMF) solvent molecules (calc. on the basis of the formula $[ZnCa(BPTC)(H_2O)(DMF)]_n{\cdot}5nDMF$ (**UCY-18**(BPTC)·5nDMF 49.8%). The next mass loss in the ~ 350 – 750 °C temperature range is assigned to the decomposition of the organic ligand $BPTC^{4-}$ and corresponds to 30.3% (calc. for **UCY-18**(BPTC)·5nDMF 30.4%) of the material's total mass. The last mass loss in the temperature range 750-850 °C is attributed to the thermal decomposition of one carbonate ligand and corresponds to 4.9% (calc. for **UCY-18**(BPTC)·5nDMF 4.8%) of the material's total mass[14,15]. Lastly, the residual mass (15.2%) at 900 °C corresponds to ZnO + CaO (calc. for **UCY-18**(BPTC)·5nDMF 15.0%).

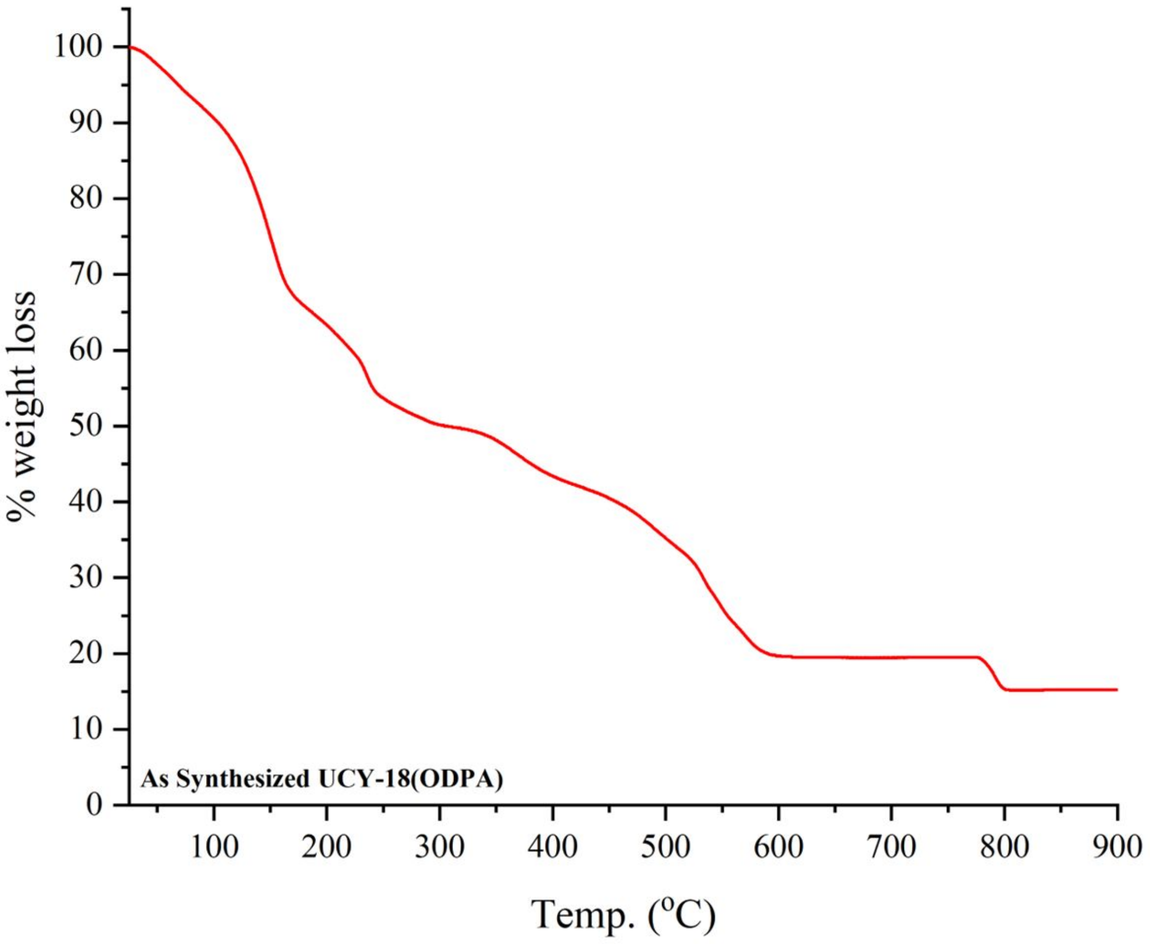

Figure S14. TGA graph of the as synthesized compound **UCY-18**(ODPA).

The TGA curve of **UCY-18**(ODPA) revealed that the thermal decomposition of this compound proceeds via a multi-step process. A rough analysis of the TGA plot shall be provided below however, there is uncertainty in this because the curve is quite complicated containing several different mass losses (guest / terminal DMF and terminal H_2O molecules and organic ligand) some of which happen in the same temperature range as evidenced from the lack of plateaus in the curve. The release of guest and coordinated solvent molecules (DMF + H_2O) involves continuous mass losses up to 380 °C whereas the last mass loss at higher temperatures is due to the decomposition of the organic ligand $ODPA^{4-}$. In particular, the mass losses in the temperature range 25 °C to 380 °C correspond to ~54.9 % of the material's total mass and are attributed the removal of terminally ligated (one DMF and one H_2O) and guest (6 DMF) solvent molecules (calc. on the basis of the formula $[ZnCa(ODPA)(H_2O)(DMF)]_n \cdot 6nDMF$ (**UCY-18**(ODPA)·6nDMF 54.2%). The next mass loss in the ~ 380 – 760 °C temperature range is assigned to the decomposition of the organic ligand $ODPA^{4-}$ and corresponds to 26.7% (calc. for **UCY-18**(ODPA)·6nDMF 27.2%) of the material's total mass. The last mass loss in the temperature range 760-850 °C is attributed to the thermal decomposition of one carbonate ligand and corresponds to 4.5% (calc. for **UCY-18**(ODPA)·6nDMF 4.5%) of the material's total mass[14,15]. Lastly, the residual mass (13.9%) at 900 °C corresponds to ZnO + CaO (calc. for **UCY-18**(ODPA)·6nDMF 14.1%).

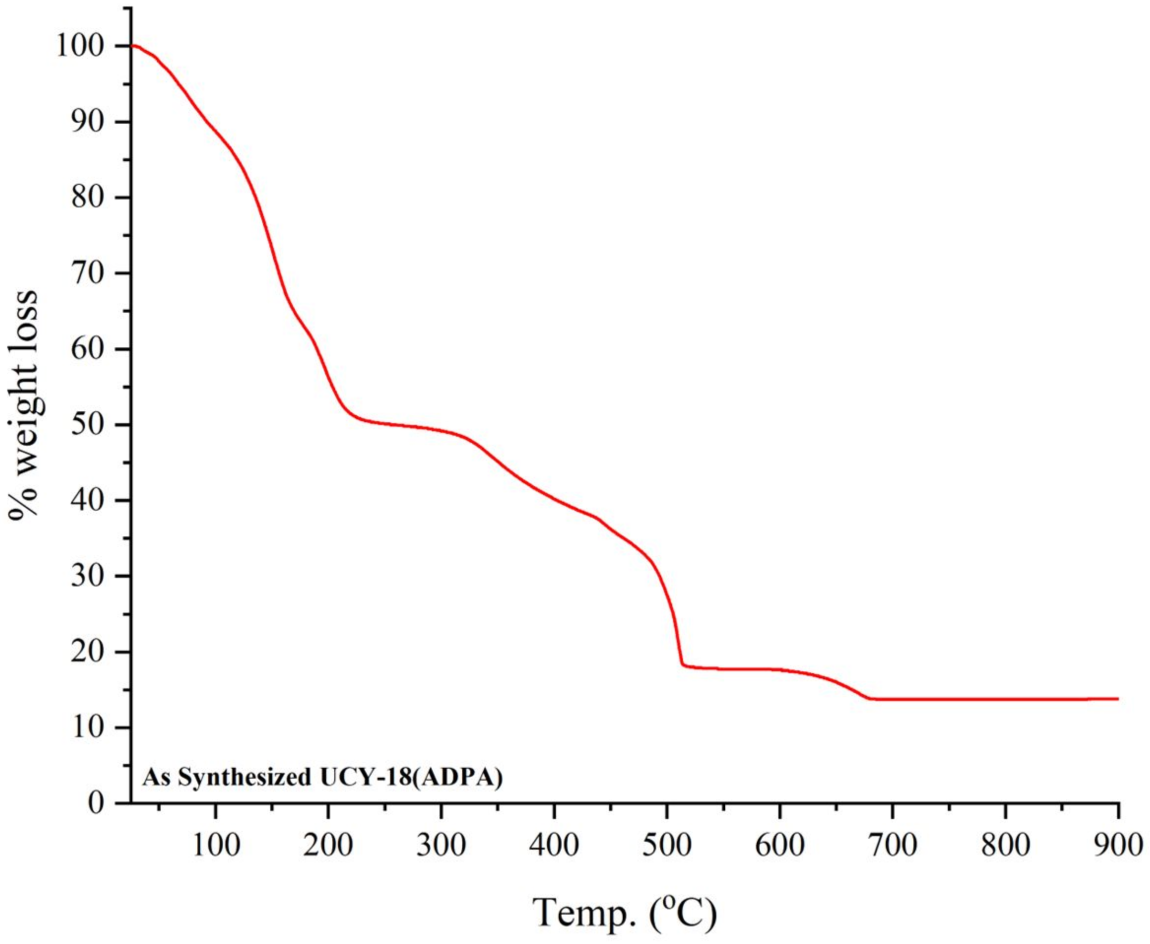

Figure S15. TGA graph of the as synthesized compound **UCY-18**(ADPA).

The TGA curve of **UCY-18**(ADPA) revealed that the thermal decomposition of this compound proceeds via a multi-step process. A rough analysis of the TGA plot shall be provided below however, there is uncertainty in this because the curve is quite complicated containing several different mass losses (guest / terminal DMF and terminal H_2O molecules and organic ligand) some of which happen in the same temperature range as evidenced from the lack of plateaus in the curve. The release of guest and coordinated solvent molecules (DMF + H_2O) involves continuous mass losses up to 370 °C whereas the last mass loss at higher temperatures is due to the decomposition of the organic ligand $ADPA^{4-}$. In particular, the mass losses in the temperature range 25 °C to 370 °C correspond to ~57.6 % of the material's total mass and are attributed the removal of terminally ligated (one DMF and one H_2O) and guest (7 DMF) solvent molecules (calc. on the basis of the formula $[ZnCa(ADPA)(H_2O)(DMF)]_n·7nDMF$ (**UCY-18**(ADPA)·7nDMF 57.4%). The next mass loss in the ~ 370 – 600 °C temperature range is assigned to the decomposition of the organic ligand $ADPA^{4-}$ and corresponds to 25.0% (calc. for **UCY-18**(ODPA)·7nDMF 25.3%) of the material's total mass. The last mass loss in the temperature range 600-720 °C is attributed to the thermal decomposition of one carbonate ligand and corresponds to 4.5% (calc. for **UCY-18**(ODPA)·7nDMF 4.2%) of the material's total mass[14,15]. Lastly, the residual mass (12.9%) at 900 °C corresponds to ZnO + CaO (calc. for **UCY-18**(ODPA)·7nDMF 13.1%).

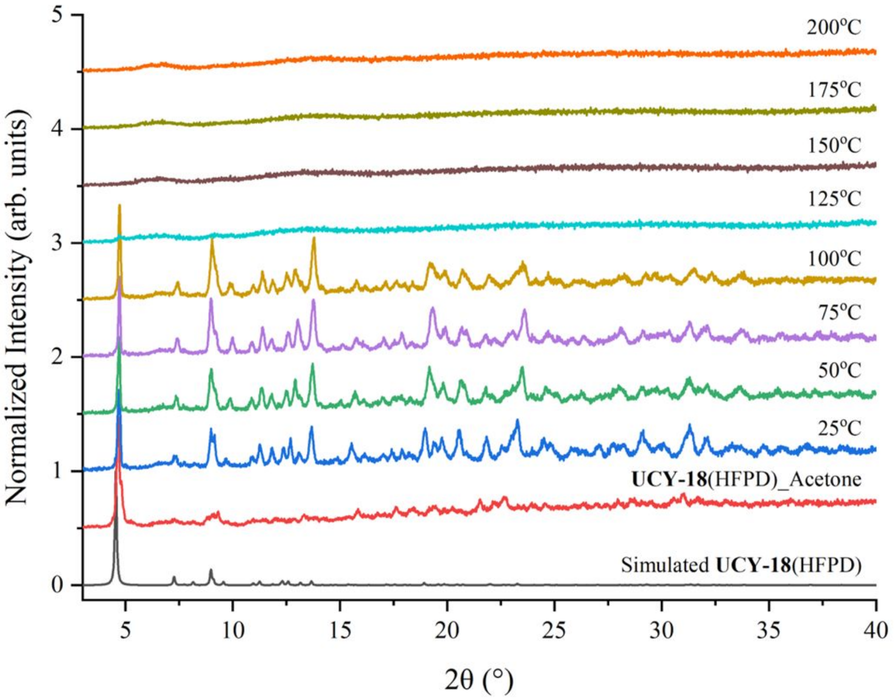

Figure S16. Variable temperature powder X-ray diffraction patterns recorded under Ar flow of the compound **UCY-18**(HFPD) treated with acetone.

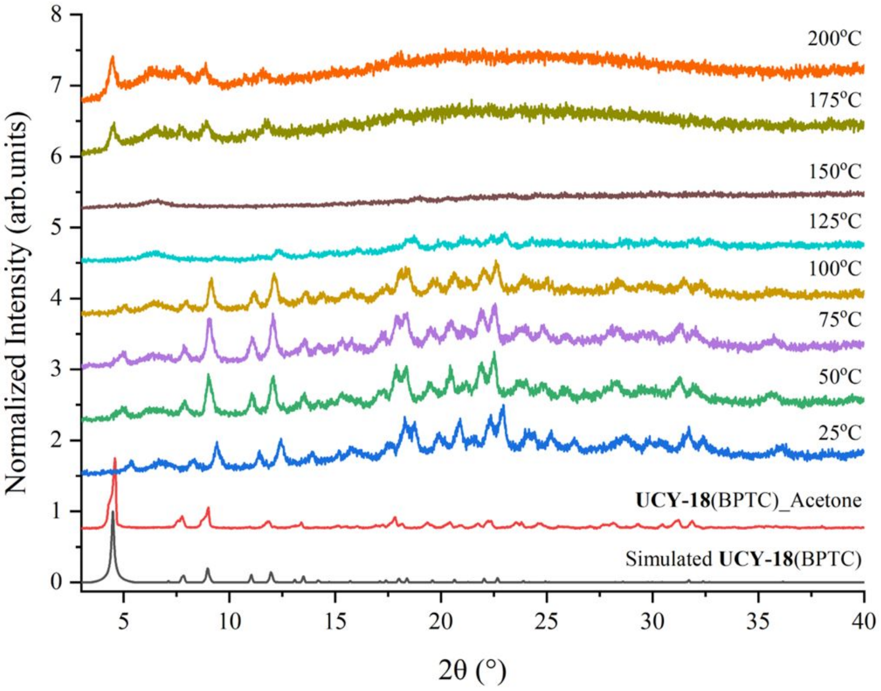

Figure S17. Variable temperature powder X-ray diffraction patterns recorded under Ar flow of the compound **UCY-18**(BPTC) treated with acetone.

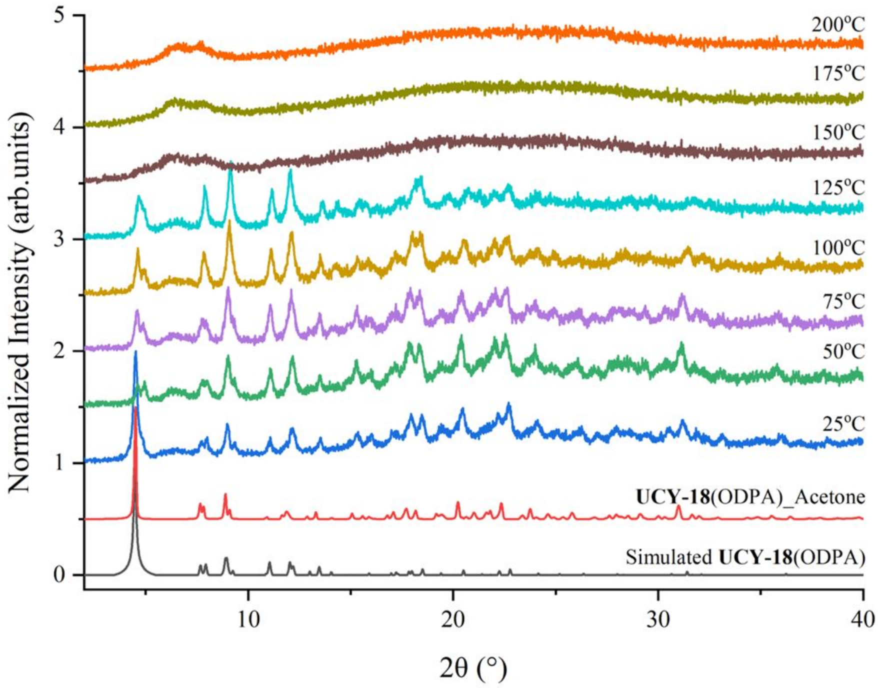

Figure S18. Variable temperature powder X-ray diffraction patterns recorded under Ar flow of the compound **UCY-18**(ODPA) treated with acetone.

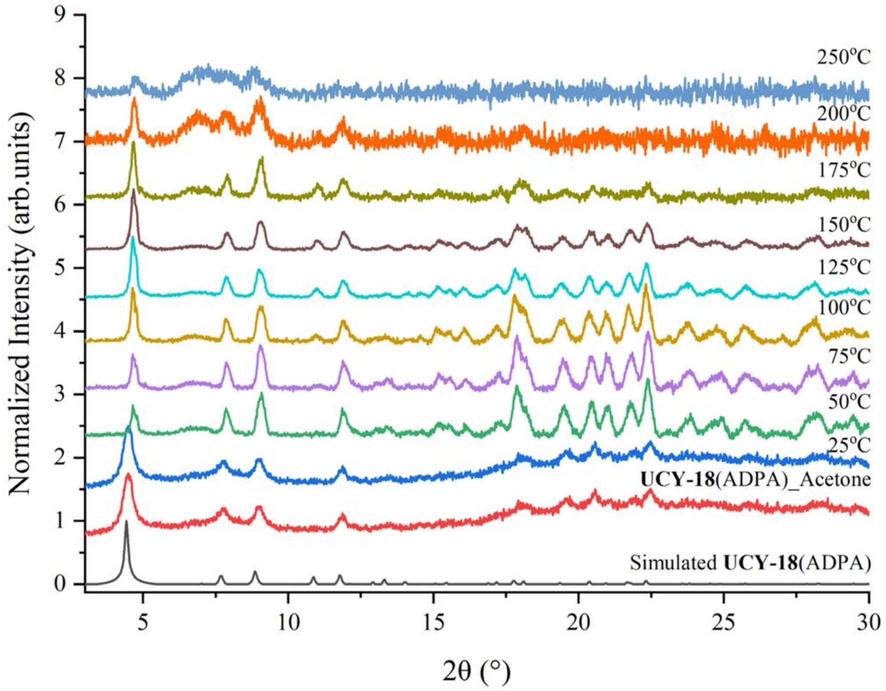

Figure S19. Variable temperature powder X-ray diffraction patterns recorded under Ar flow of the compound **UCY-18**(ADPA) treated with acetone.

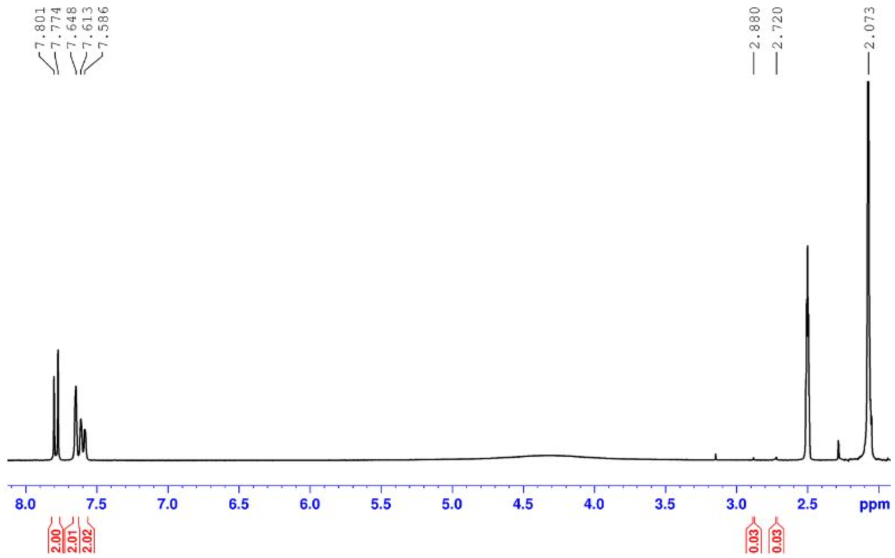

Figure S20. ^{1}H-NMR spectrum of compound **UCY-18**(HFPD) treated with acetone, digested in 10 μL DCl (35% wt in D_2O) in d_6-DMSO. These data indicate the removal of DMF molecules from compound **UCY-18**(HFPD). ^{1}H-NMR (d_6-DMSO): δ 2.72 (s, 3H, C**H**$_3$, DMF), δ 2.88 (s, 3H, C**H**$_3$, DMF), δ 7.58-7.61 (d, 2H, Ar-**H**, HFPD^{4-}), δ 7.65 (s, 2H, Ar-**H**, HFPD^{4-}), δ 7.80-7.77 (d, 2H, Ar-**H**, HFPD^{4-}).

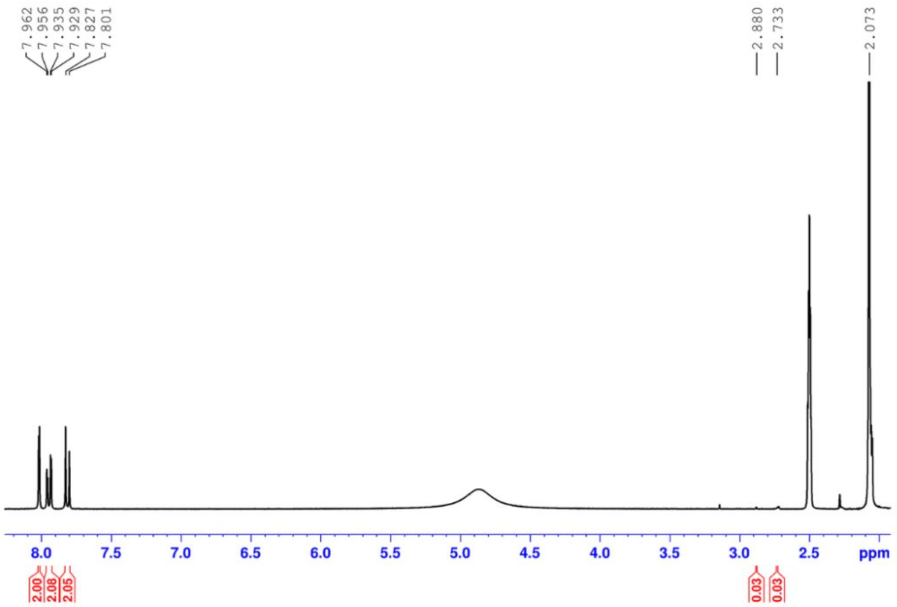

Figure S21. ^{1}H-NMR spectrum of compound **UCY-18**(BPTC) treated with acetone, digested in 10 μL DCl (35% wt in D_2O) in d_6-DMSO. These data indicate the removal of DMF molecules from compound **UCY-18**(BPTC). ^{1}H-NMR (d_6-DMSO): δ 2.73 (s, 3H, C**H**$_3$, DMF), δ 2.88 (s, 3H, C**H**$_3$, DMF), δ 7.82-7.80 (d, 2H, Ar-**H**, BPTC^{4-}), δ 7.96-7.93 (dd, 2H, Ar-**H**, BPTC^{4-}), δ 8.01 (d, 2H, Ar-**H**, BPTC^{4-}).

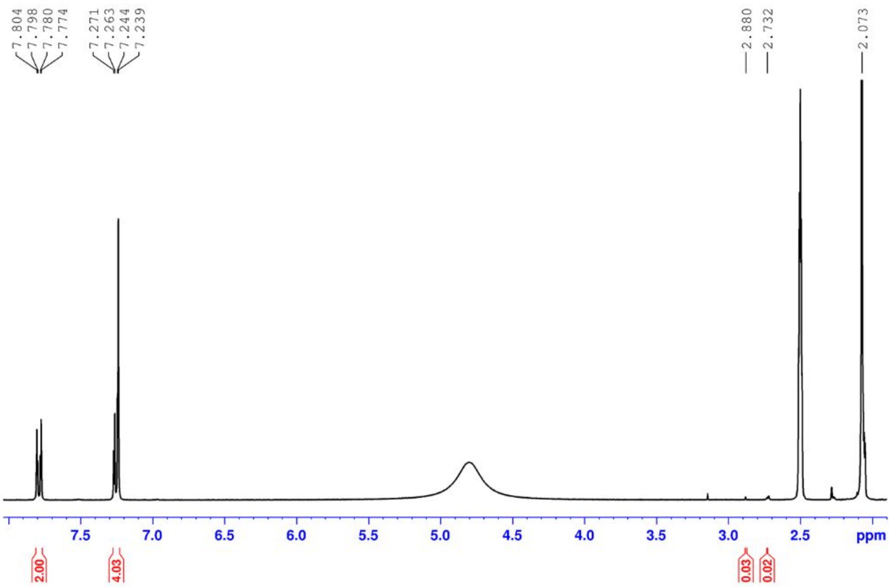

Figure S22. ^{1}H-NMR spectrum of compound **UCY-18**(ODPA) treated with acetone, digested in 10 μL DCl (35% wt in D_2O) in d_6-DMSO. These data indicate the removal of DMF molecules from compound **UCY-18**(ODPA). ^{1}H-NMR (d_6-DMSO): δ 2.73 (s, 3H, C**H**$_3$, DMF), δ 2.88 (s, 3H, C**H**$_3$, DMF), δ 7.24 (d, 2H, Ar-**H**, ODPA^{4-}), δ 7.27-7.26 (d, 2H, Ar-**H**, ODPA^{4-}), δ 7.80-7.77 (dd, 2H, Ar-**H**, ODPA^{4-}).

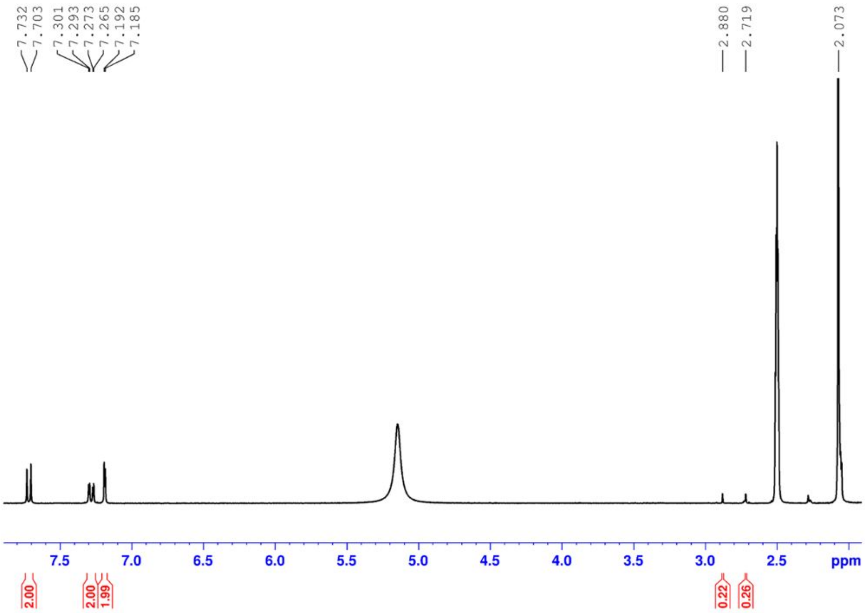

Figure S23. ^{1}H-NMR spectrum of compound **UCY-18**(ADPA) treated with acetone, digested in 10 μL DCl (35% wt in D_2O) in d_6-DMSO. These data indicate the removal of DMF molecules from compound **UCY-18**(ADPA). ^{1}H-NMR (d_6-DMSO): δ 2.72 (s, 3H, C**H**$_3$, DMF), δ 2.88 (s, 3H, C**H**$_3$, DMF), δ 7.19-7.18 (d, 2H, Ar-**H**, ADPA^{4-}), δ 7.30-7.26 (dd, 2H, Ar-**H**, ADPA^{4-}), δ 7.73-7.70 (d, 2H, Ar-**H**, ADPA^{4-}).

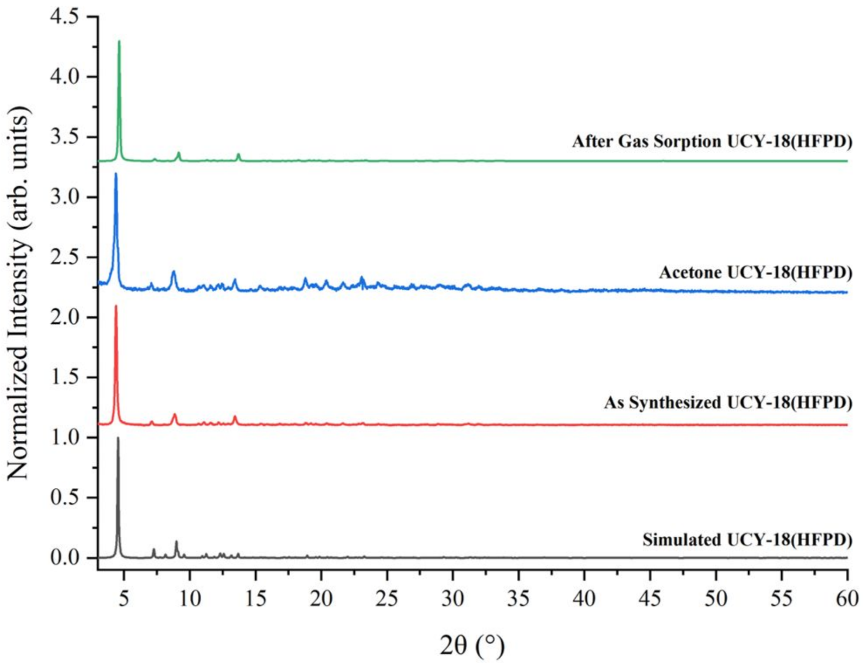

Figure S24. Powder X-ray diffraction patterns of the as synthesized, treated with acetone and activated (collected after the completion of gas sorption studies) compound **UCY-18**(HFPD).

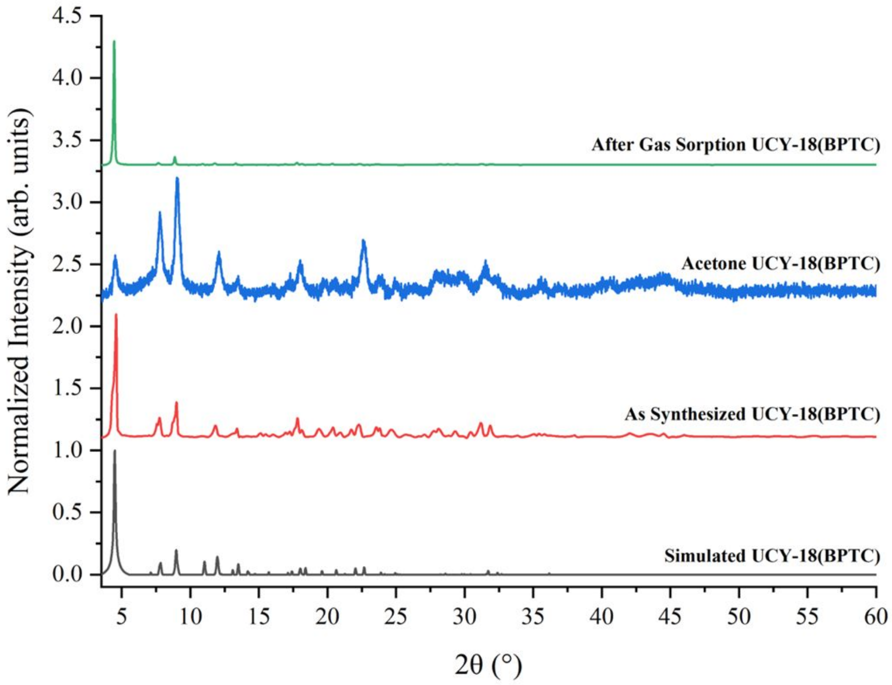

Figure S25. Powder X-ray diffraction patterns of the as synthesized, treated with acetone and activated (collected after the completion of gas sorption studies) compound **UCY-18**(BPTC).

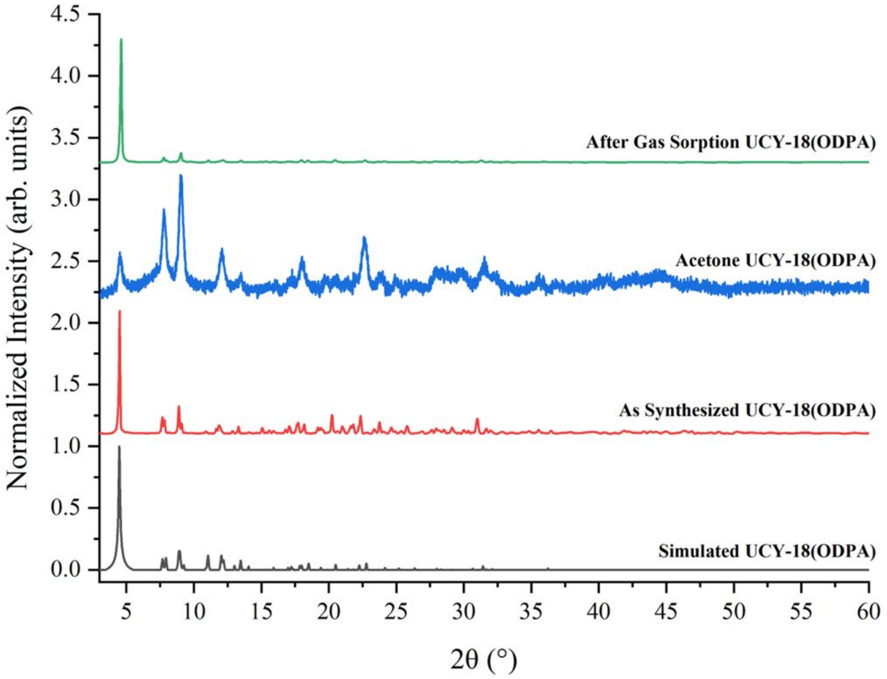

Figure S26. Powder X-ray diffraction patterns of the as synthesized, treated with acetone and activated (collected after the completion of gas sorption studies) compound **UCY-18**(ODPA).

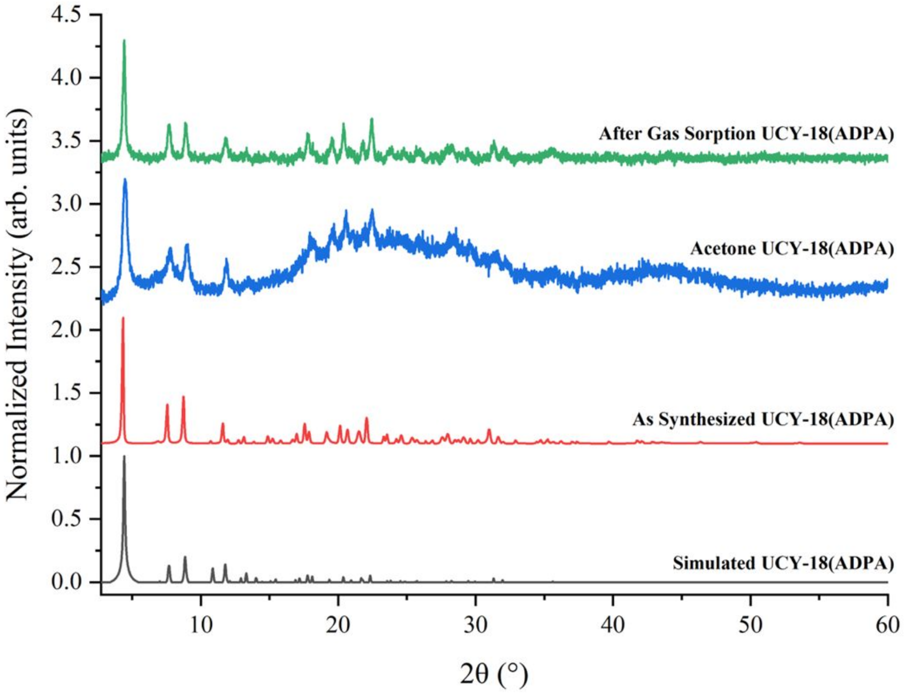

Figure S27. Powder X-ray diffraction patterns of the as synthesized, treated with acetone and activated (collected after the completion of gas sorption studies) compound **UCY-18**(ADPA).

Gas Sorption Measurements

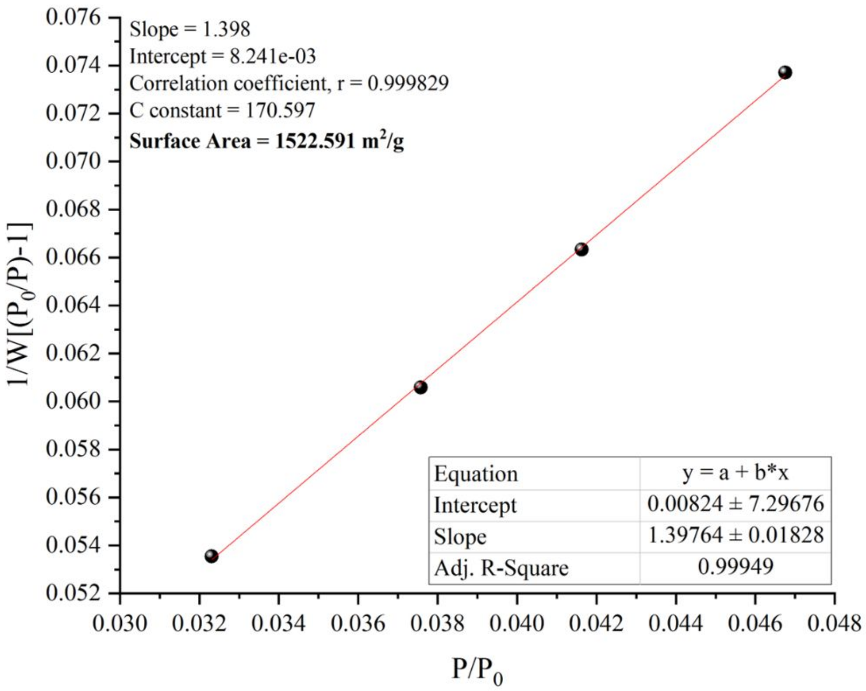

Figure S28. BET plot from Ar adsorption isotherm at 87K for **UCY-18**(HFPD).

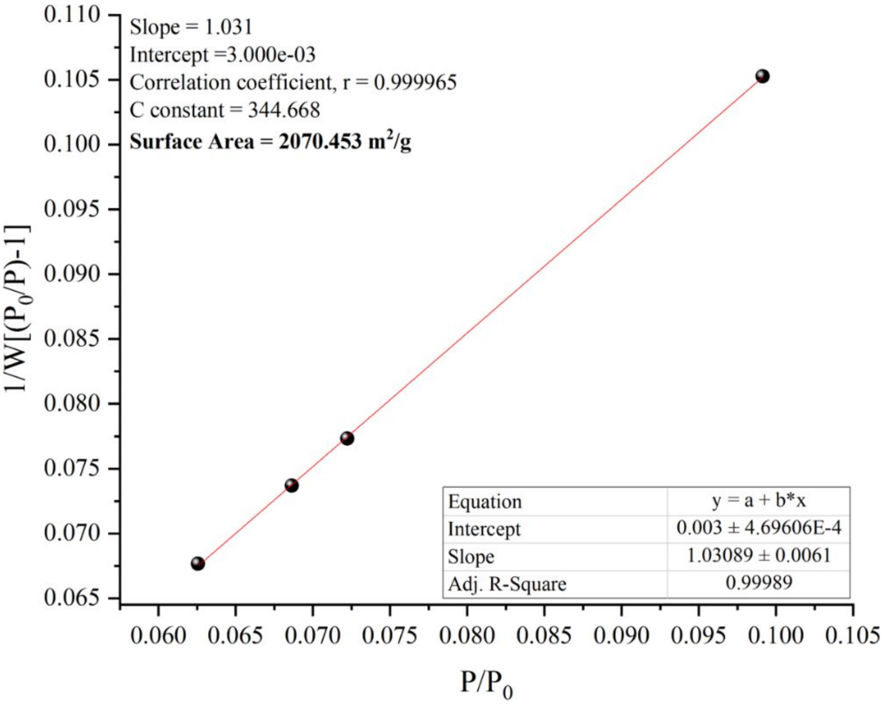

Figure S29. BET plot from Ar adsorption isotherm at 87K for **UCY-18**(BPTC).

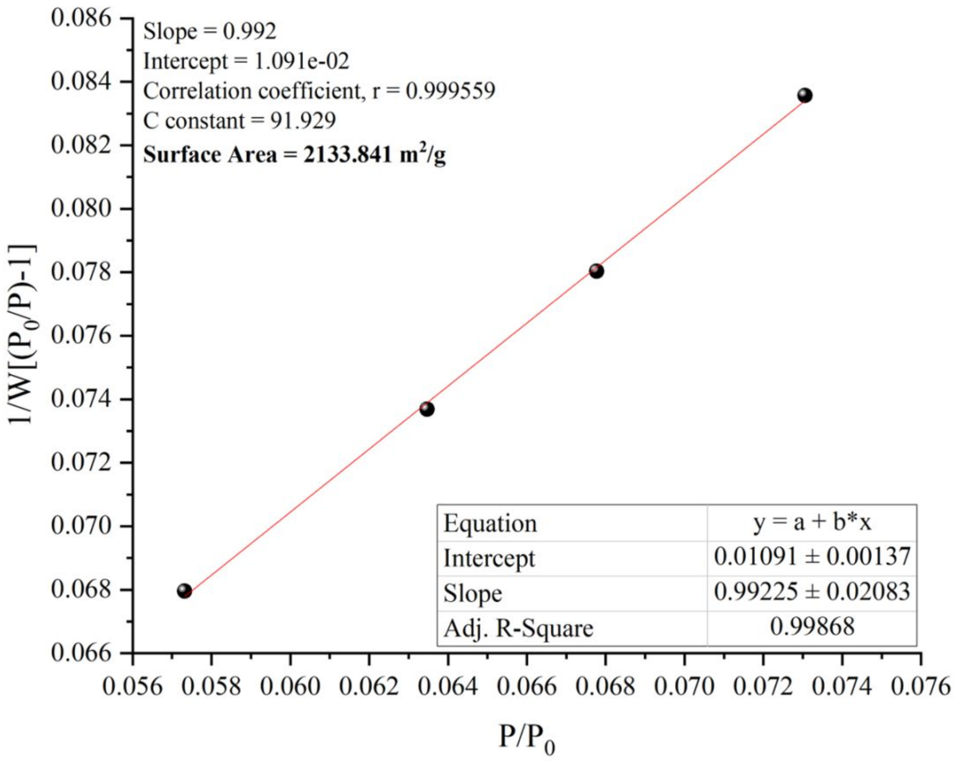

Figure S30. BET plot from Ar adsorption isotherm at 87K for **UCY-18**(ODPA).

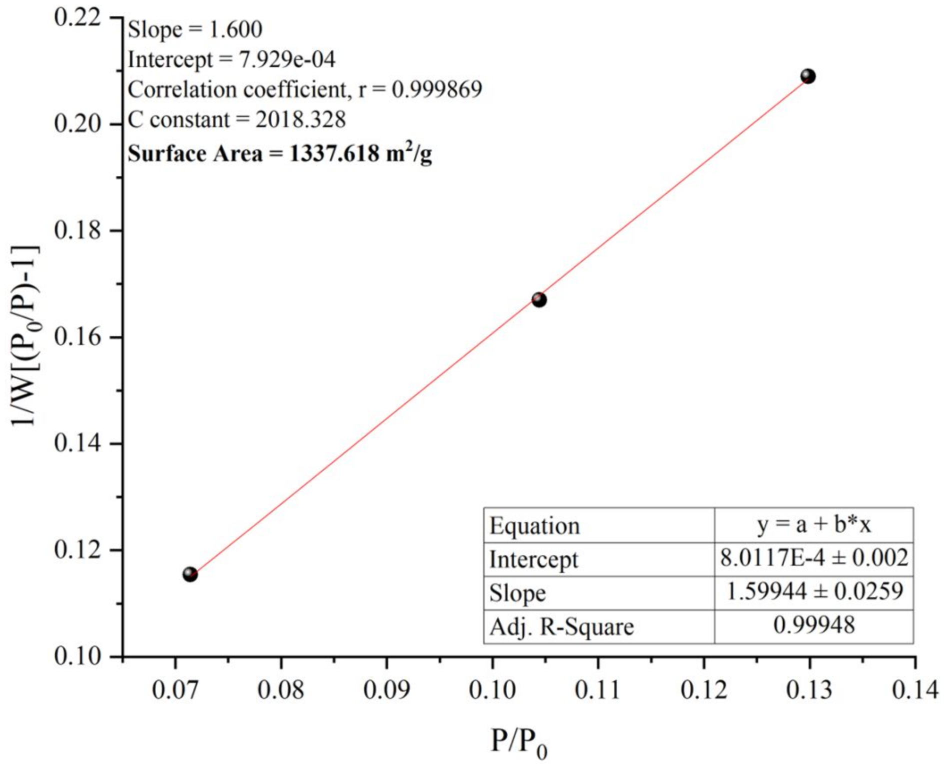

Figure S31. BET plot from Ar adsorption isotherm at 87K for **UCY-18**(ADPA).

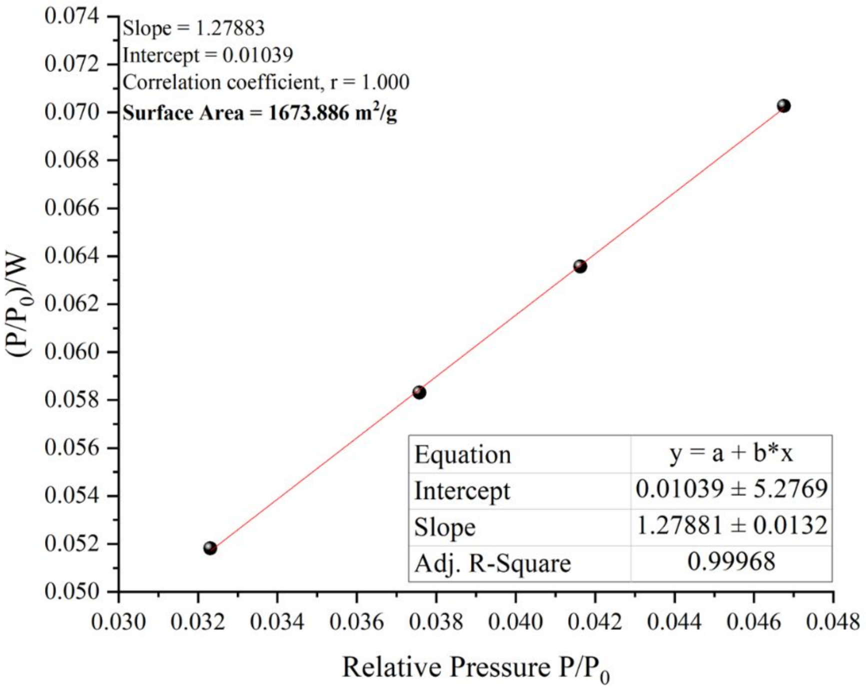

Figure S32. Langmuir plot from Ar adsorption isotherm at 87K for **UCY-18**(HFPD).

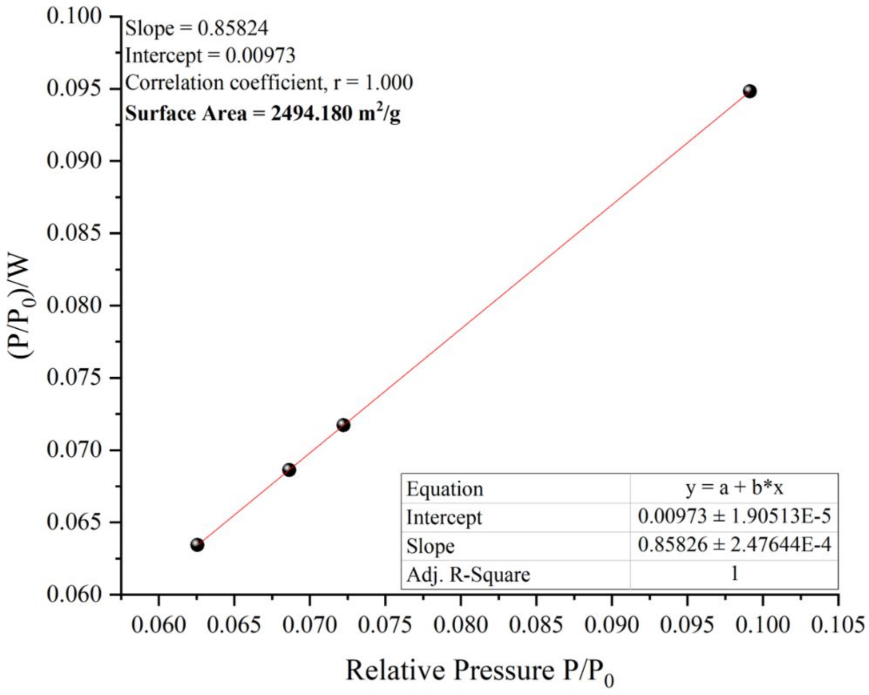

Figure S33. Langmuir plot from Ar adsorption isotherm at 87K for **UCY-18**(BPTC).

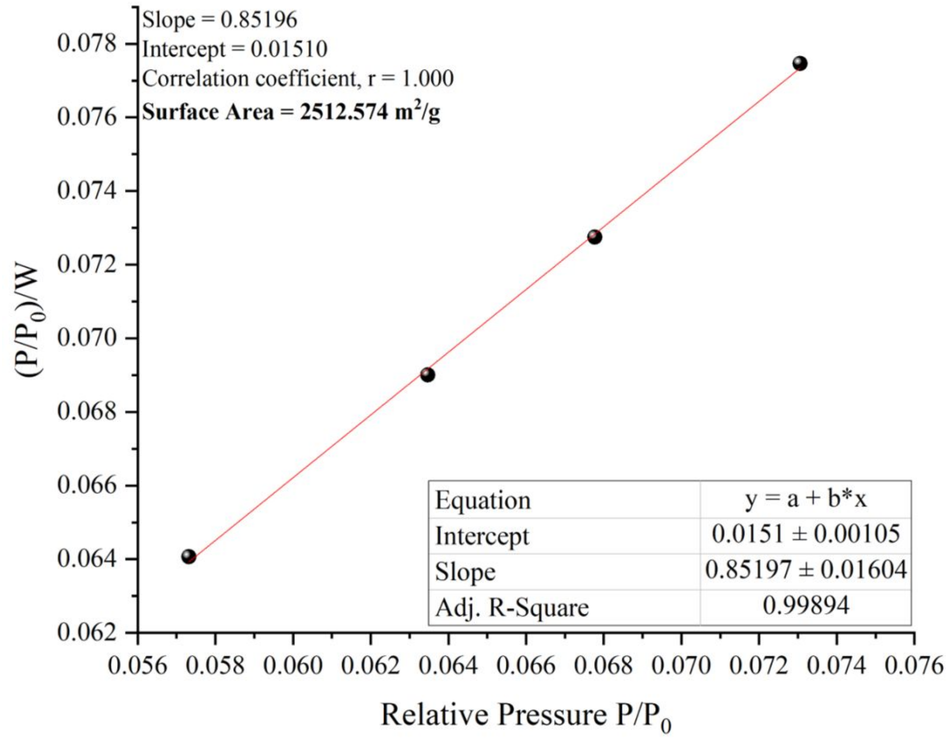

Figure S34. Langmuir plot from Ar adsorption isotherm at 87K for **UCY-18**(ODPA).

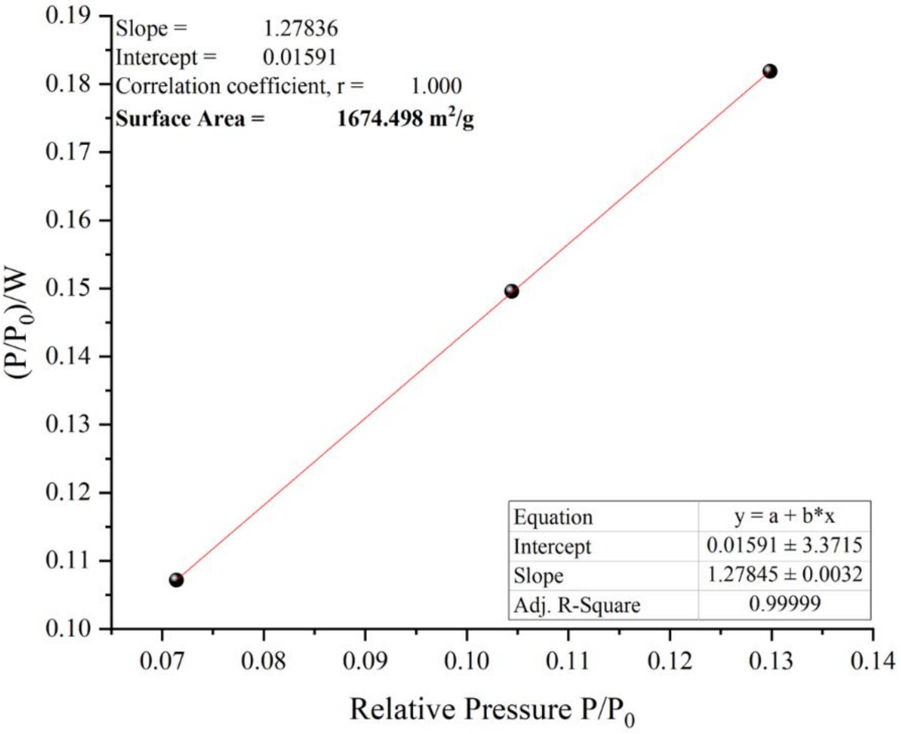

Figure S35. Langmuir plot from Ar adsorption isotherm at 87K for **UCY-18**(ADPA).

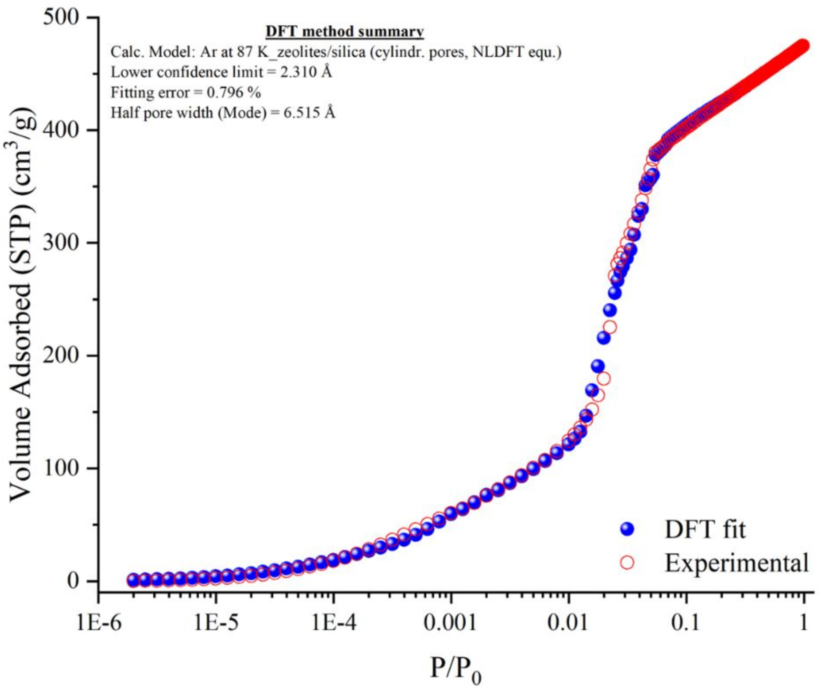

Figure S36. Ar adsorption isotherm recorded at 87K and the corresponding NLDFT fitting for **UCY-18**(HFPD).

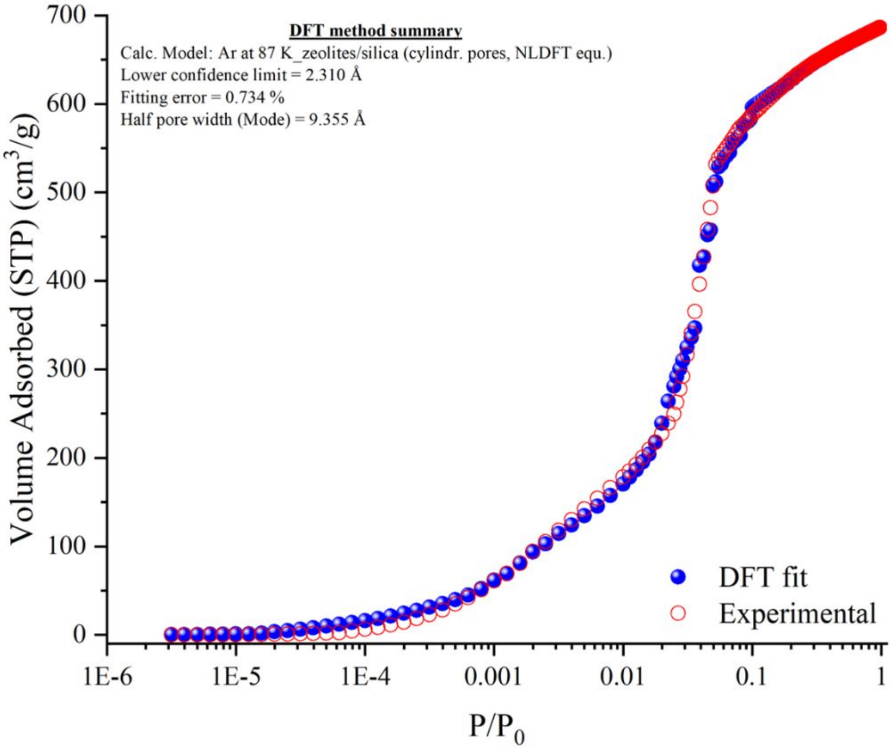

Figure S37. Ar adsorption isotherm recorded at 87K and the corresponding NLDFT fitting for **UCY-18**(BPTC).

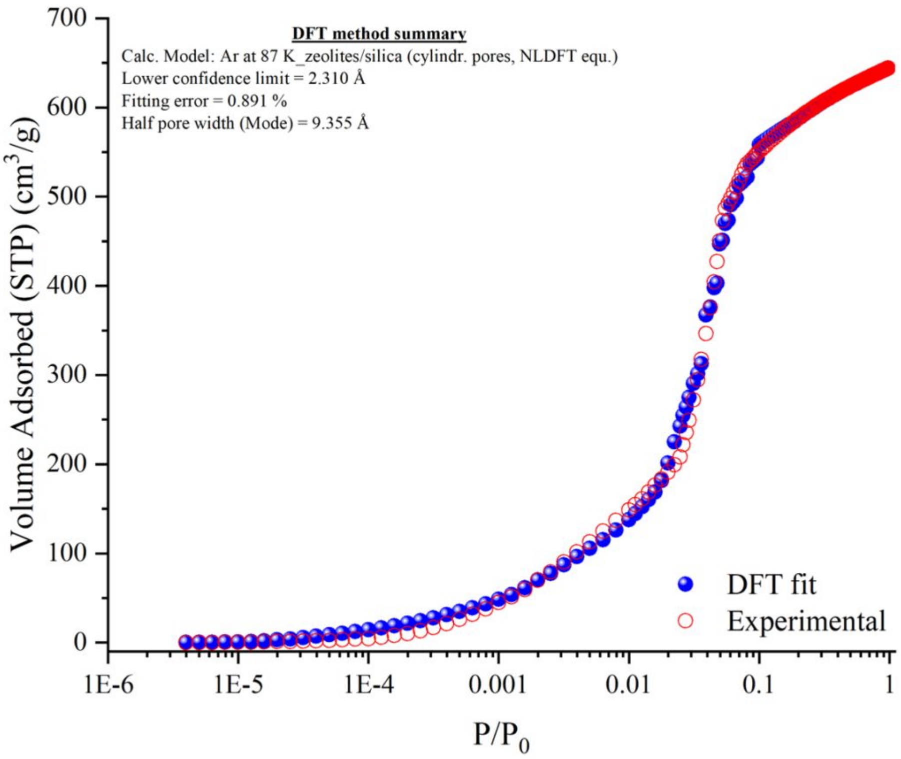

Figure S38. Ar adsorption isotherm recorded at 87K and the corresponding NLDFT fitting for **UCY-18**(ODPA).

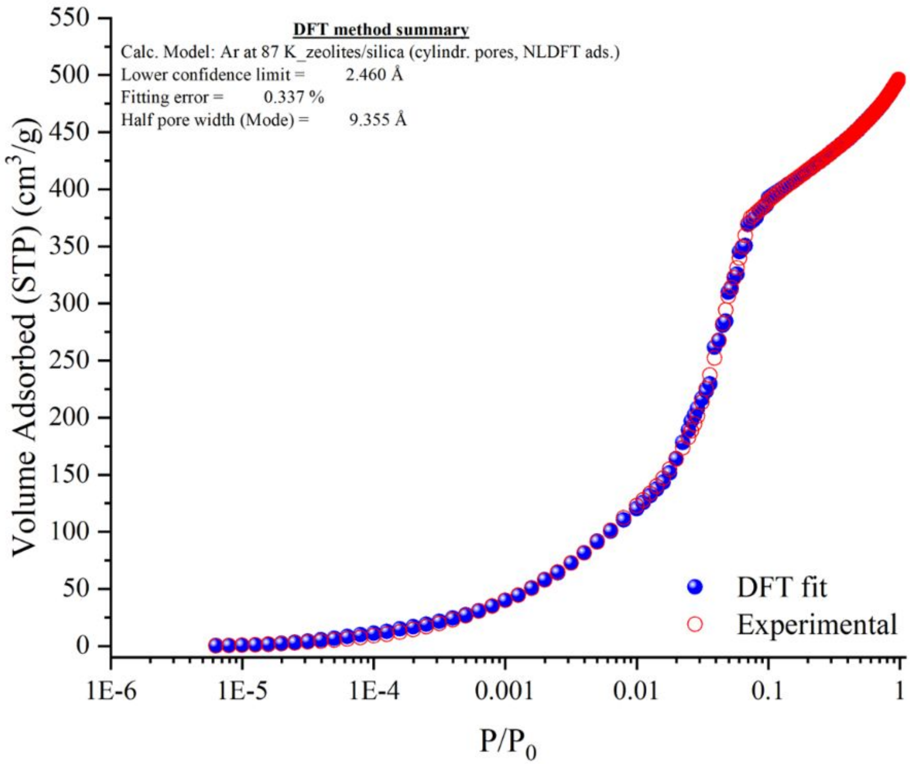

Figure S39. Ar adsorption isotherm recorded at 87K and the corresponding NLDFT fitting for **UCY-18**(ADPA).

Low pressure CO_2, CH_4 and H_2, sorption isotherms, determination of heat adsorption.

Heat of adsorption: To calculate heats of adsorptions, the corresponding adsorption isotherms at three different temperatures (273 K, 283 K and 298K for CO_2 and CH_4) and at two different temperatures (77K and 87 K for H_2) were simultaneously fitted using the virial type[16,17] Equation 1:

$$\ln P = \ln N + \frac{1}{T}\sum_{i=0}^{m} a_i N^i + \sum_{i=0}^{n} b_i N^i \quad (1)$$

The heat of adsorption at zero coverage was calculated from Equation 2, where as a function of surface coverage, from Equation 3:

$$Q_{st} = -Ra_o \quad (2)$$

$$Q_{st}(N) = -R\sum_{i=0}^{m} a_i N^i \quad (3)$$

For the determination of the isosteric heat of adsorption using Clausious – Clapeyron equation a commercially available software, ASiQwin (version 3.01) purchased from Quantachrome, was used.

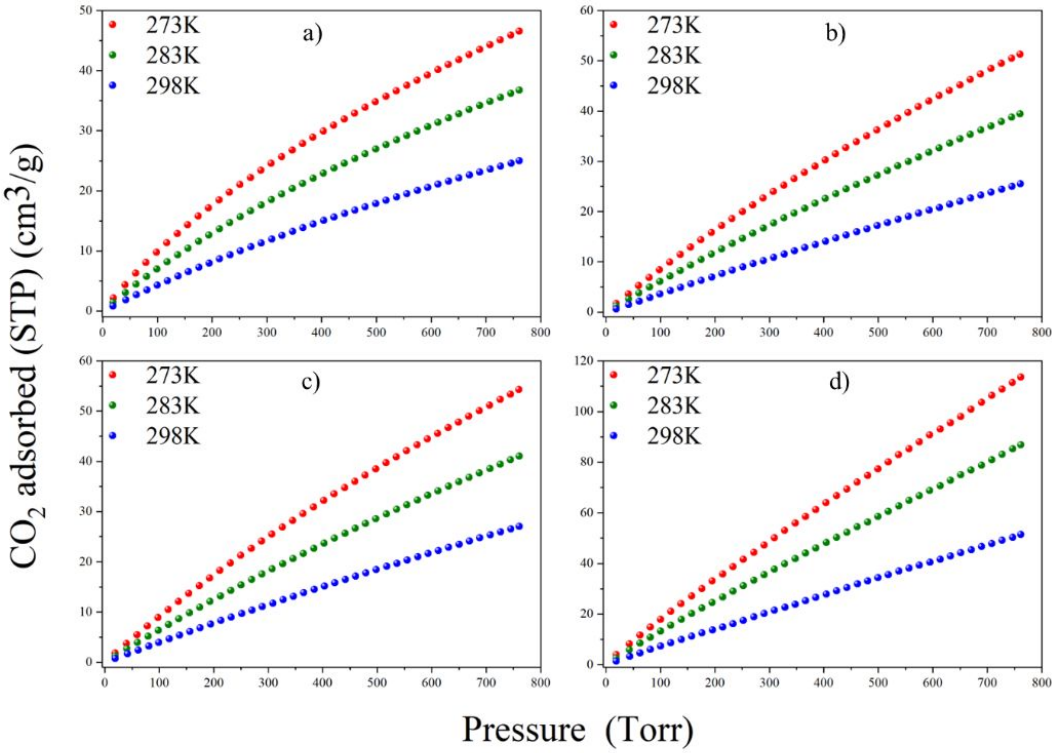

Figure S40. CO_2 adsorption isotherms of a) **UCY-18**)(HFPD), b) **UCY-18**)(BPTC), c) **UCY-18**)(ODPA) and d) **UCY-18**)(ADPA) at 273 K, 283 K and 298 K.

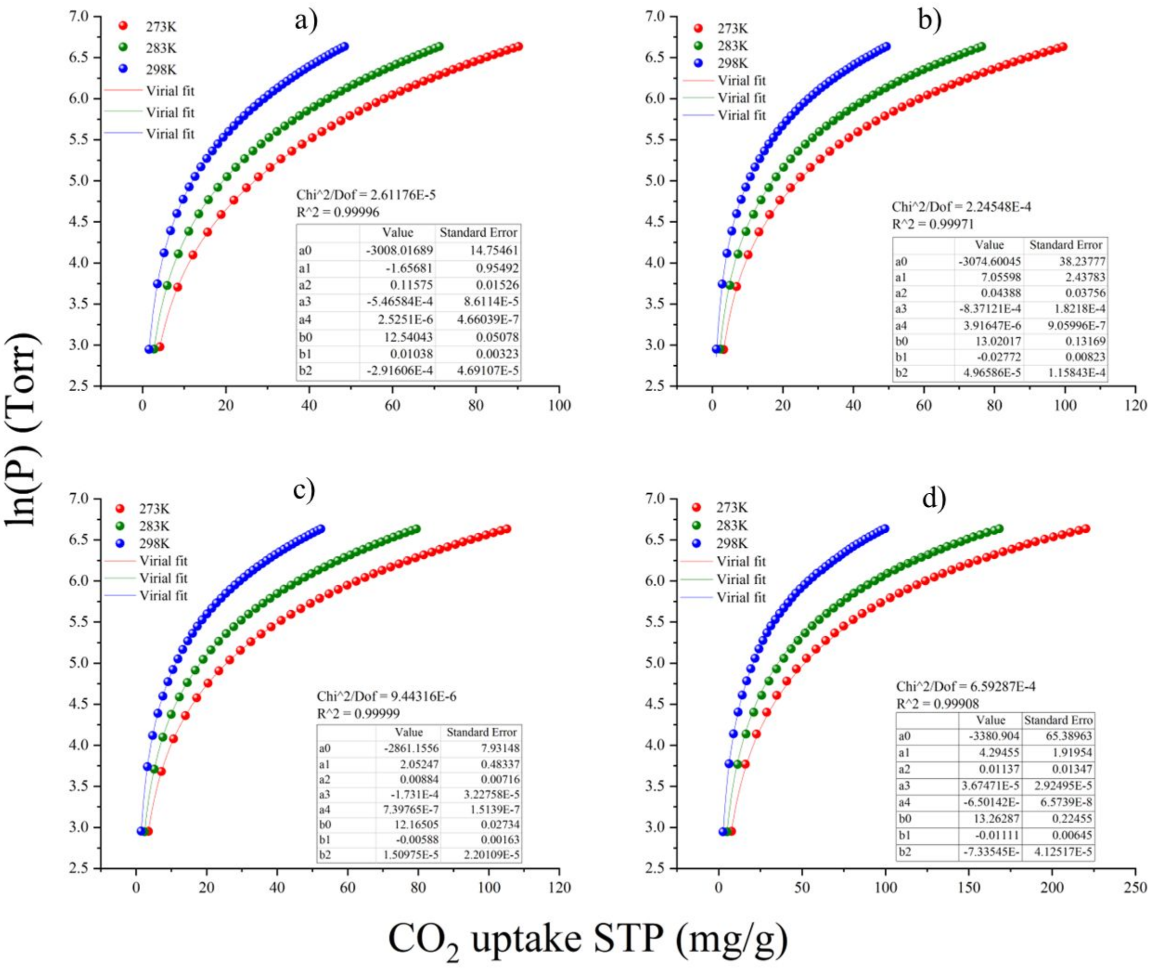

Figure S41. Virial type fitting of CO_2 adsorption isotherms of a) **UCY-18**)(HFPD), b) **UCY-18**)(BPTC), c) **UCY-18**)(ODPA) and d) **UCY-18**)(ADPA) at 273 K, 283 K and 298 K according to equation 1.

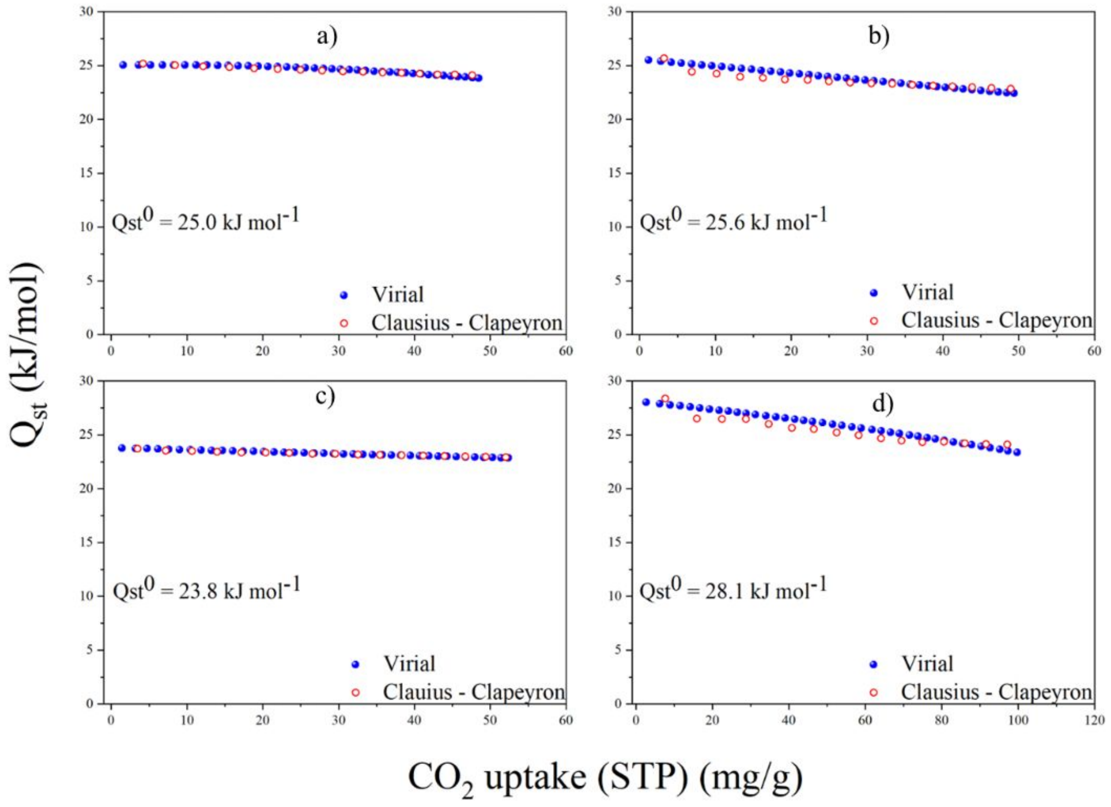

Figure S42. CO_2 isosteric heat of adsorption in a) **UCY-18**)(HFPD), b) **UCY-18**)(BPTC), c) **UCY-18**)(ODPA) and d) **UCY-18**)(ADPA) as a function of surface coverage.

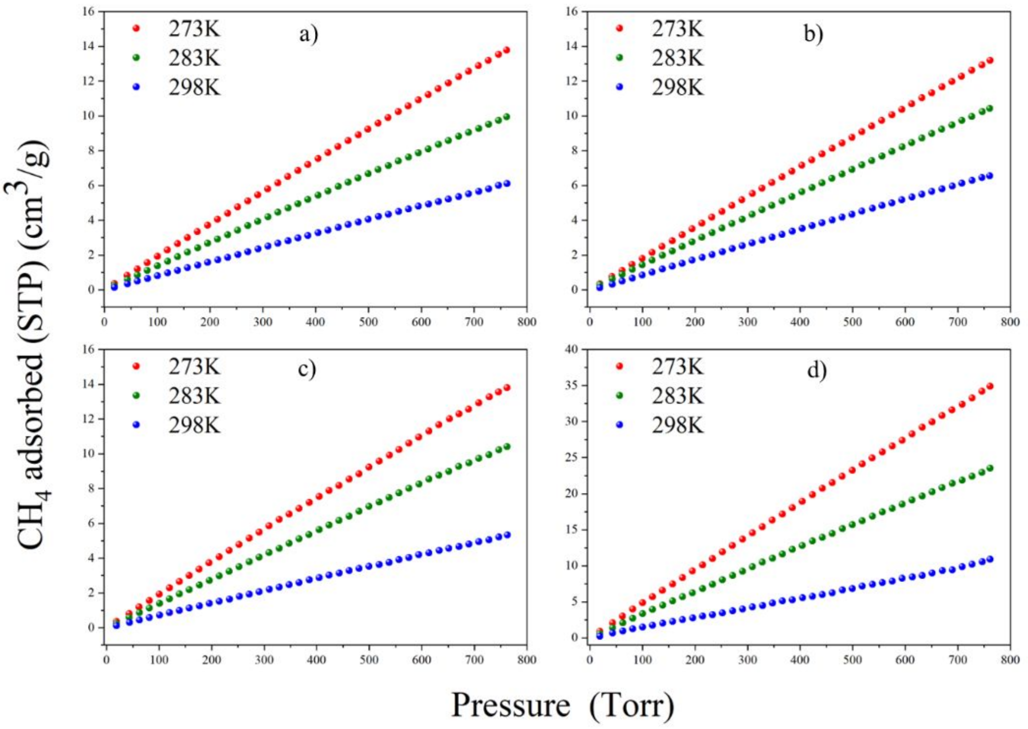

Figure S43. CH_4 adsorption isotherms of a) **UCY-18**)(HFPD), b) **UCY-18**)(BPTC), c) **UCY-18**)(ODPA) and d) **UCY-18**)(ADPA) at 273 K, 283 K and 298 K.

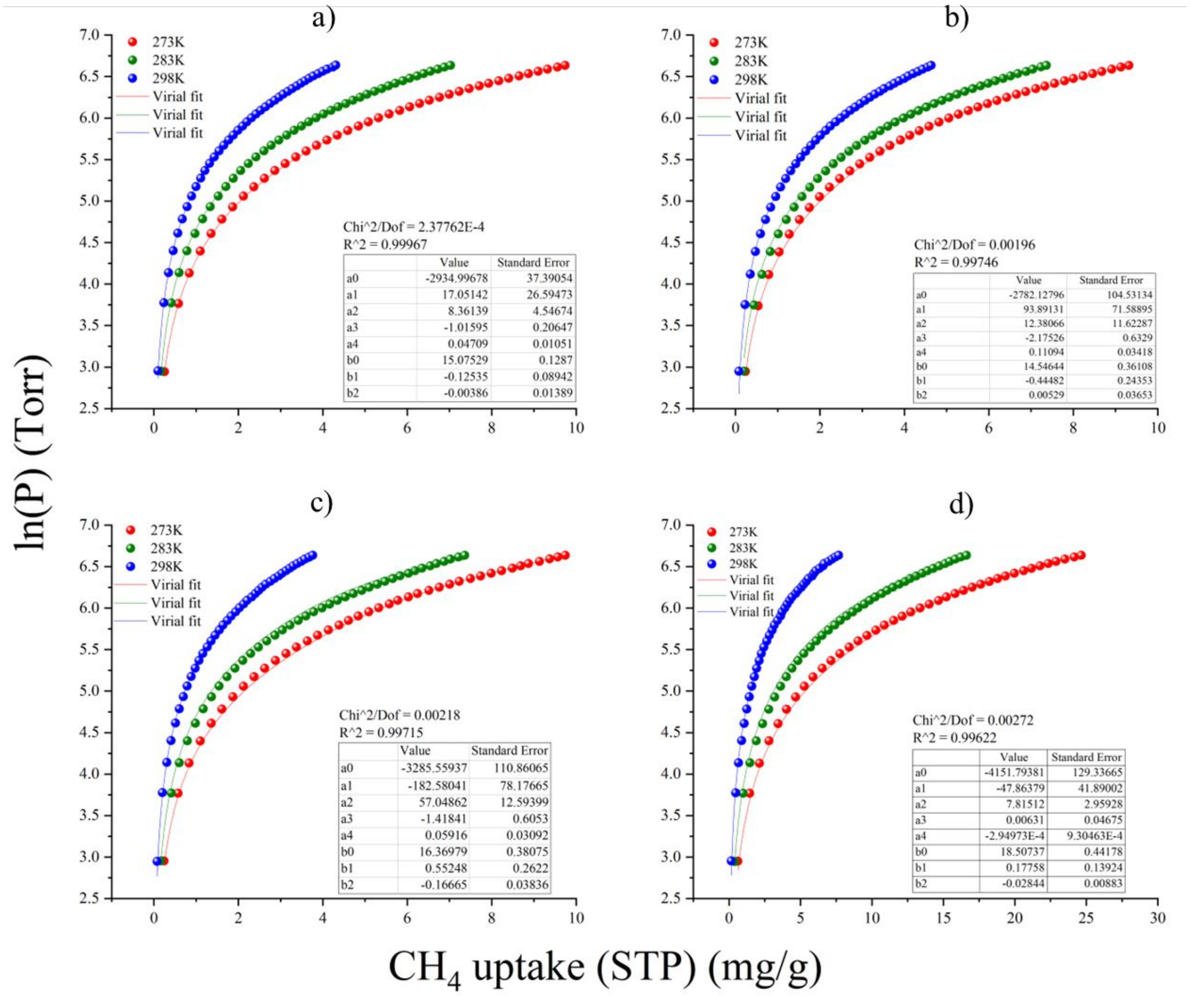

Figure S44. Virial type fitting of CH_4 adsorption isotherms of a) **UCY-18**)(HFPD), b) **UCY-18**)(BPTC), c) **UCY-18**)(ODPA) and d) **UCY-18**)(ADPA) at 273 K, 283 K and 298 K according to equation 1.

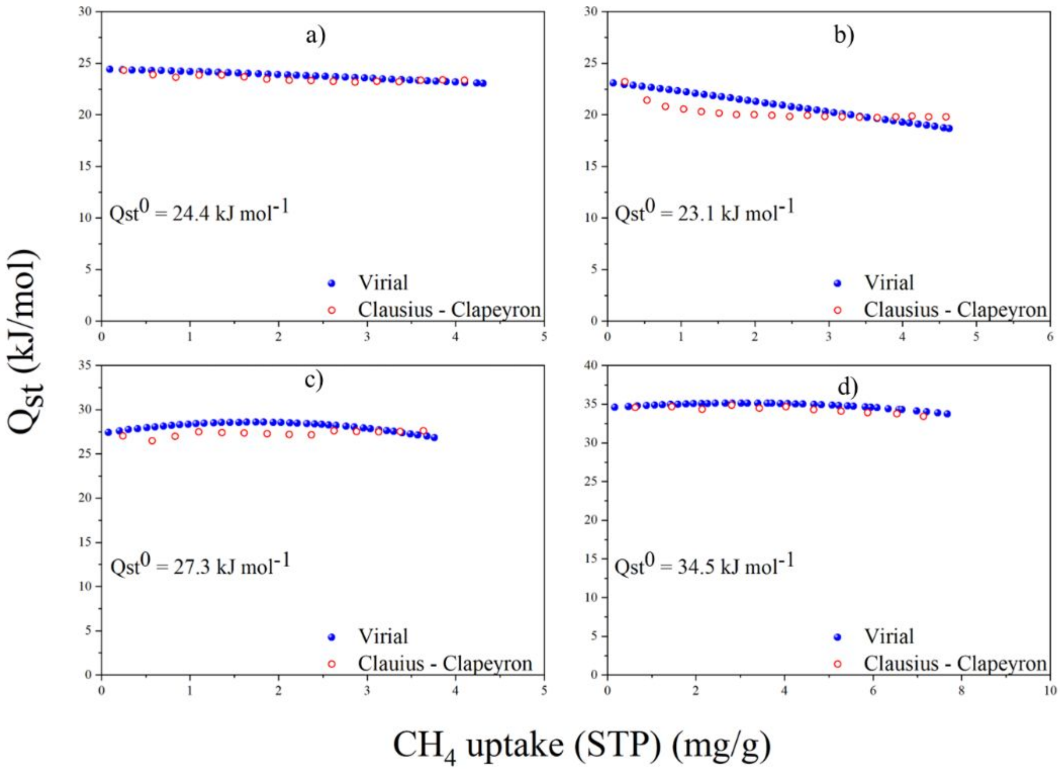

Figure S45. CH_4 isosteric heat of adsorption in a) **UCY-18**)(HFPD), b) **UCY-18**)(BPTC), c) **UCY-18**)(ODPA) and d) **UCY-18**)(ADPA) as a function of surface coverage.

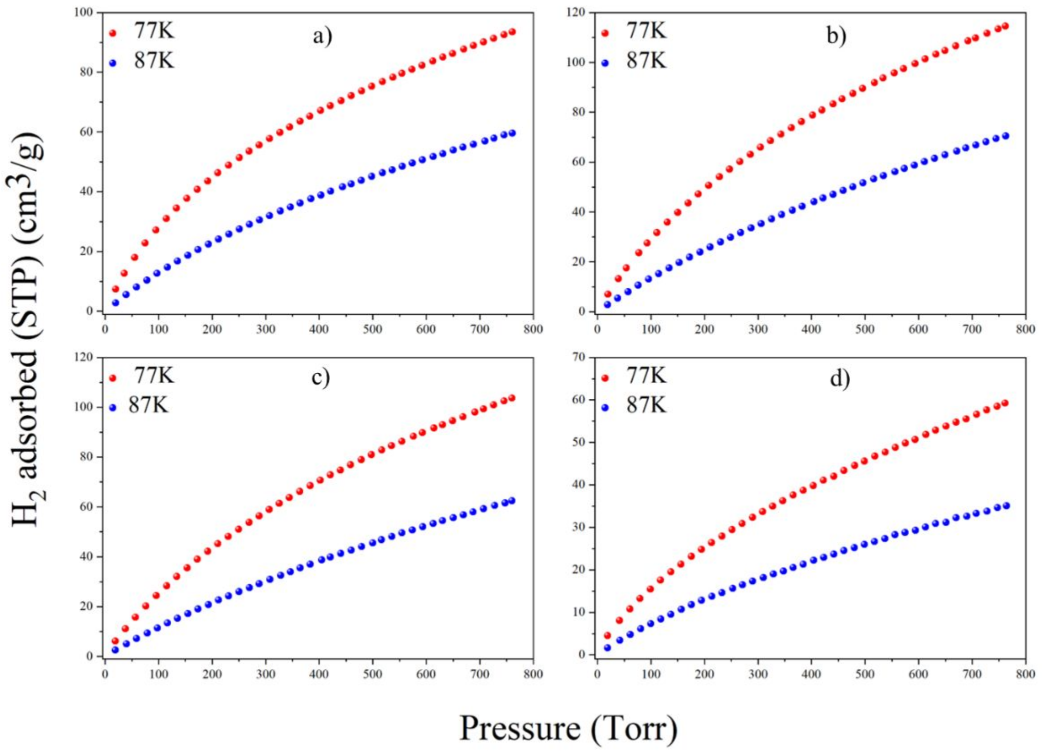

Figure S46. H_2 adsorption isotherms of a) **UCY-18**)(HFPD), b) **UCY-18**)(BPTC), c) **UCY-18**)(ODPA) and d) **UCY-18**)(ADPA) at 77 K and 87 K.

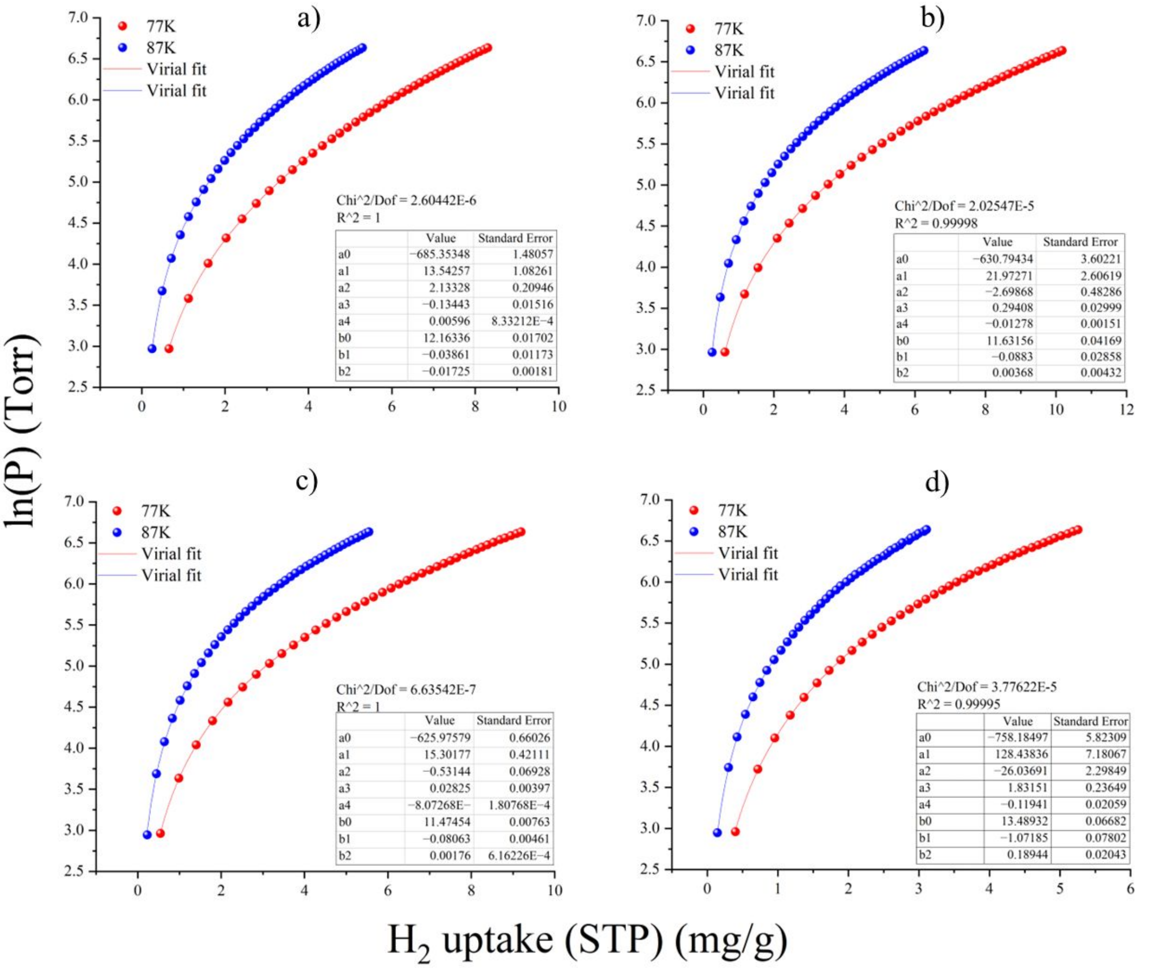

a)

	Value	Standard Error
a0	−685.35348	1.48057
a1	13.54257	1.08261
a2	2.13328	0.20946
a3	−0.13443	0.01516
a4	0.00596	8.33212E−4
b0	12.16336	0.01702
b1	−0.03861	0.01173
b2	−0.01725	0.00181

b)

	Value	Standard Error
a0	−630.79434	3.60221
a1	21.97271	2.60619
a2	−2.69868	0.48286
a3	0.29408	0.02999
a4	−0.01278	0.00151
b0	11.63156	0.04169
b1	−0.0883	0.02858
b2	0.00368	0.00432

c)

	Value	Standard Error
a0	−625.97579	0.66026
a1	15.30177	0.42111
a2	−0.53144	0.06928
a3	0.02825	0.00397
a4	−8.07268E−	1.80768E−4
b0	11.47454	0.00763
b1	−0.08063	0.00461
b2	0.00176	6.16226E−4

d)

	Value	Standard Error
a0	−758.18497	5.82309
a1	128.43836	7.18067
a2	−26.03691	2.29849
a3	1.83151	0.23649
a4	−0.11941	0.02059
b0	13.48932	0.06682
b1	−1.07185	0.07802
b2	0.18944	0.02043

Figure S47. Virial type fitting of H_2 adsorption isotherms of a) **UCY-18**)(HFPD), b) **UCY-18**)(BPTC), c) **UCY-18**)(ODPA) and d) **UCY-18**)(ADPA) at 77 K and 87 K according to equation 1.

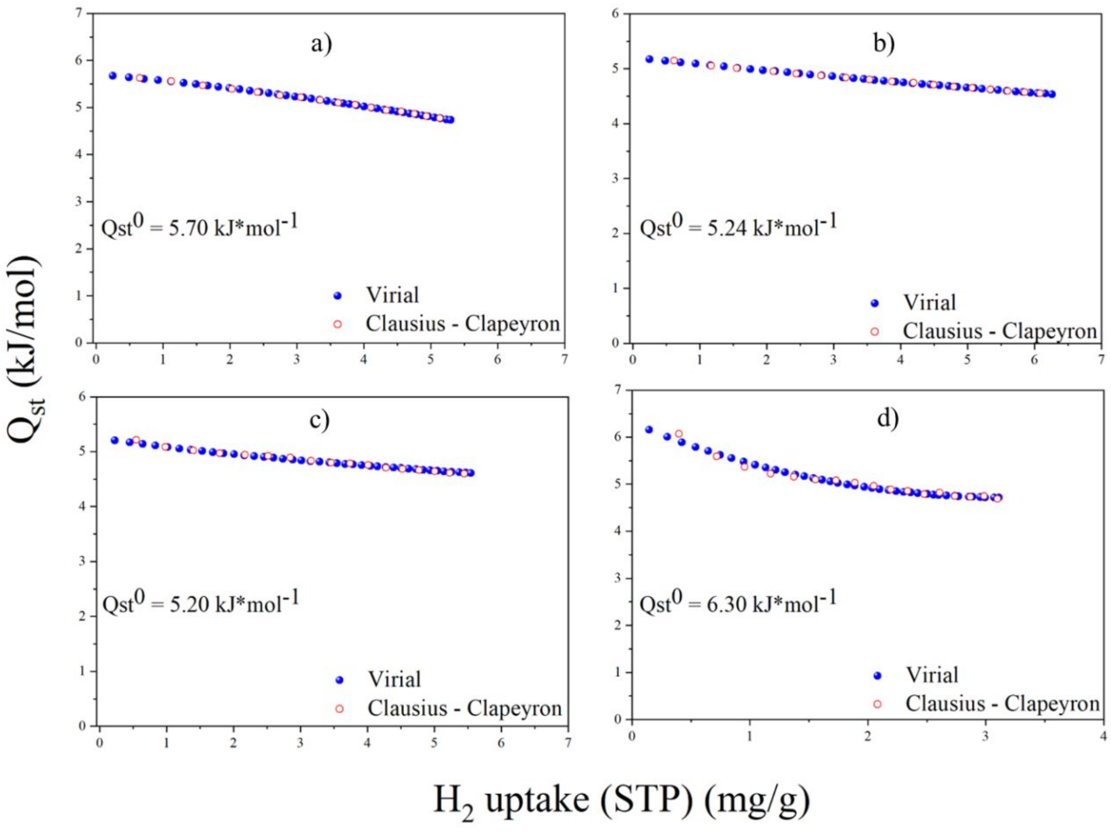

Figure S48. H_2 isosteric heat of adsorption in a) **UCY-18**)(HFPD), b) **UCY-18**)(BPTC), c) **UCY-18**)(ODPA) and d) **UCY-18**)(ADPA) as a function of surface coverage.

TABLE S8. CO_2, CH_4 and H_2 adsorption capabilities at various temperature and up to 1 bar

Compound	Temperature (K)	Uptake (cm^3 g^{-1})	Uptake (mmol g^{-1})	Uptake (wt %)	Qst^0 (kJ mol^{-1})
**UCY18**(HFPD)/CO_2	273	46.5	2.1	9.0	25.0
**UCY18**(HFPD)/CO_2	283	36.8	1.6	7.1	
**UCY18**(HFPD)/CO_2	298	25.0	1.1	4.8	
**UCY18**(HFPD)/CH_4	273	13.8	0.6	1.0	24.4
**UCY18**(HFPD)/CH_4	283	9.9	0.4	0.7	
**UCY18**(HFPD)/CH_4	298	6.1	0.3	0.4	
**UCY18**(HFPD)/H_2	77	93.5	4.1	0.8	5.70
**UCY18**(HFPD)/H_2	87	59.6	2.6	0.5	
**UCY18**(BPTC)/CO_2	273	51.3	2.3	9.9	25.6
**UCY18**(BPTC)/CO_2	283	39.4	1.7	7.6	
**UCY18**(BPTC)/CO_2	298	25.5	1.1	4.9	
**UCY18**(BPTC)/CH_4	273	13.2	0.6	0.9	23.1
**UCY18**(BPTC)/CH_4	283	10.4	0.5	0.7	
**UCY18**(BPTC)/CH_4	298	6.5	0.3	0.5	
**UCY18**(BPTC)/H_2	77	114.6	5.1	1.0	5.24
**UCY18**(BPTC)/H_2	87	70.5	3.1	0.6	
**UCY18**(ODPA)/CO_2	273	54.2	2.4	10.5	23.8
**UCY18**(ODPA)/CO_2	283	41.0	1.8	7.9	
**UCY18**(ODPA)/CO_2	298	27.0	1.2	5.2	
**UCY18**(ODPA)/CH_4	273	13.8	0.6	1.0	27.3
**UCY18**(ODPA)/CH_4	283	10.4	0.5	0.7	
**UCY18**(ODPA)/CH_4	298	5.3	0.2	0.4	
**UCY18**(ODPA)/H_2	77	103.6	4.6	0.9	5.20
**UCY18**(ODPA)/H_2	87	62.5	2.8	0.6	
**UCY18**(ADPA)/CO_2	273	113.6	5.0	22.0	28.1
**UCY18**(ADPA)/CO_2	283	86.8	3.8	16.8	
**UCY18**(ADPA)/CO_2	298	51.5	2.3	10.0	
**UCY18**(ADPA)/CH_4	273	34.9	1.5	2.5	34.5
**UCY18**(ADPA)/CH_4	283	23.5	1.1	1.7	
**UCY18**(ADPA)/CH_4	298	10.9	0.5	0.8	
**UCY18**(ADPA)/H_2	77	59.2	2.6	0.5	6.30
**UCY18**(ADPA)/H_2	87	35.0	1.5	0.3	

Photoluminescence Studies

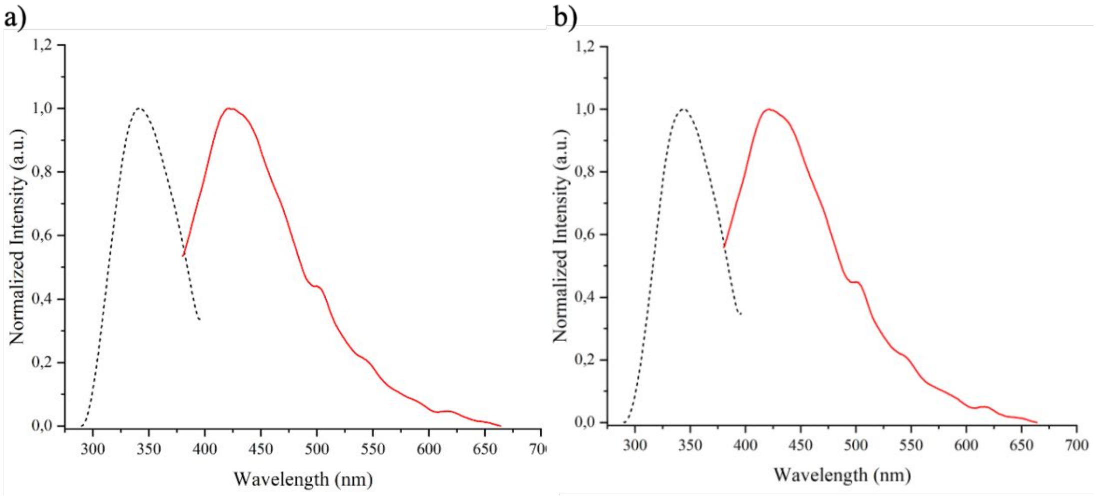

Figure S49. Normalized solid state a) excitation (λ_{em}= 425 nm) and emission spectra (λ_{exc}= 345 nm) of 4,4′-HFPD and b) excitation (λ_{em} = 425 nm) and emission spectra (λ_{exc} = 345 nm) of **UCY-18**(HFPD).

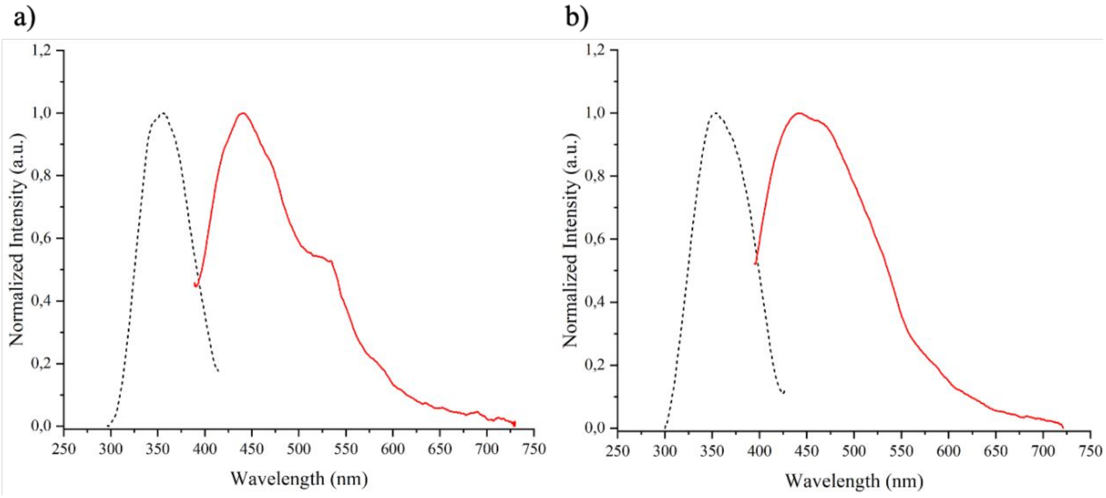

Figure S50. Normalized solid state a) excitation (λ_{em}= 438 nm) and emission spectra (λ_{exc}= 370 nm) of 3,3′,4,4′-BPTD and b) excitation (λ_{em} = 450 nm) and emission spectra (λ_{exc} = 370 nm) of **UCY-18**(BPTC).

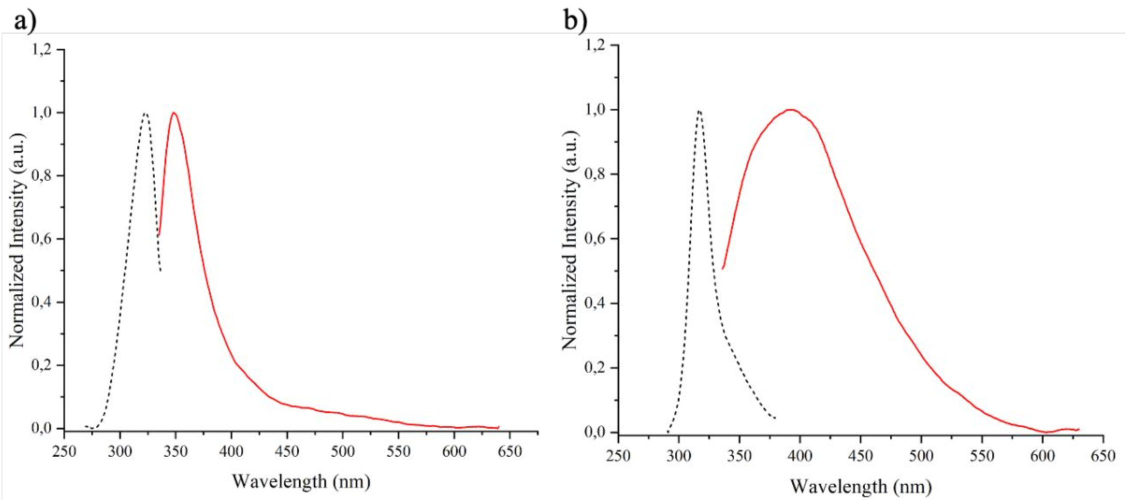

Figure S51. Normalized solid state a) excitation (λ_{em}= 350 nm) and emission spectra (λ_{exc}= 324 nm) of 4,4′-ODPA and b) excitation (λ_{em} = 400 nm) and emission spectra (λ_{exc} = 320 nm) of **UCY-18**(ODPA).

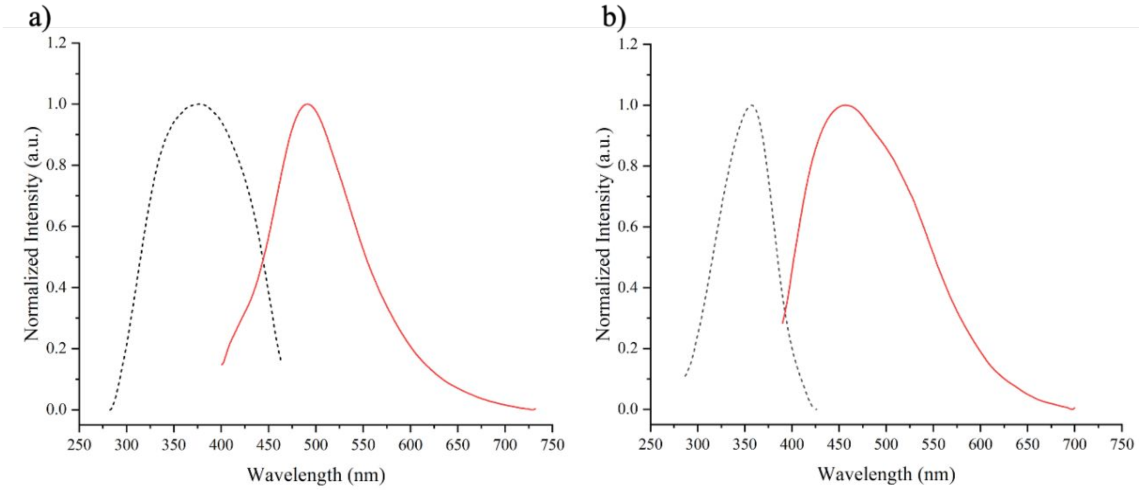

Figure S52. Normalized solid state a) excitation (λ_{em}= 490.5 nm) and emission spectra (λ_{exc}= 376 nm) of H_4ADPA and b) excitation (λ_{em} = 456.5 nm) and emission spectra (λ_{exc} = 361 nm) of **UCY-18**(ADPA).

Thin film characterization and sensing studies

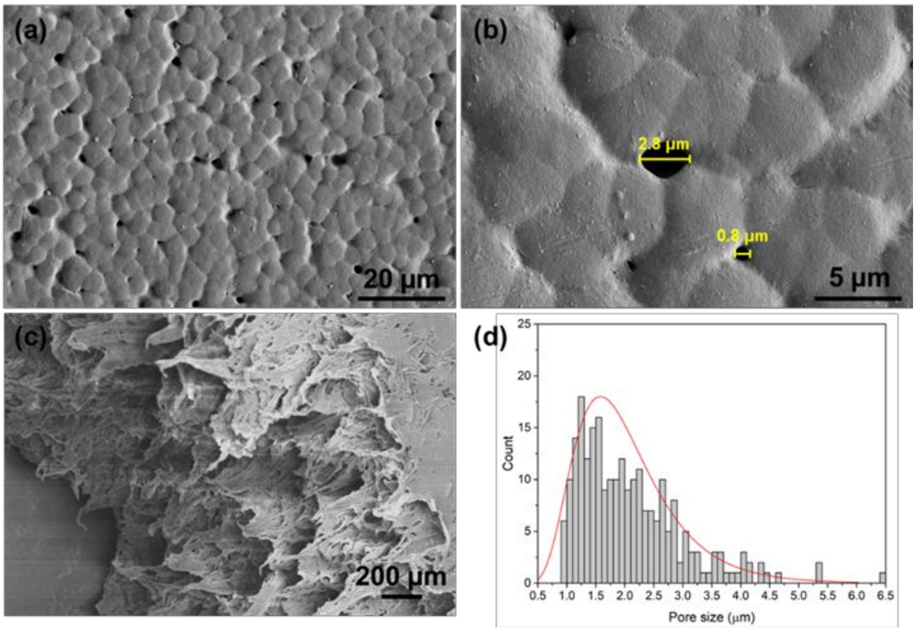

Figure S53. SEM images of top-view at (a) low magnification, (b) high magnification, and (c) cross-section of an empty PVDF membrane. (d) Pore size distribution of the PVDF membrane.

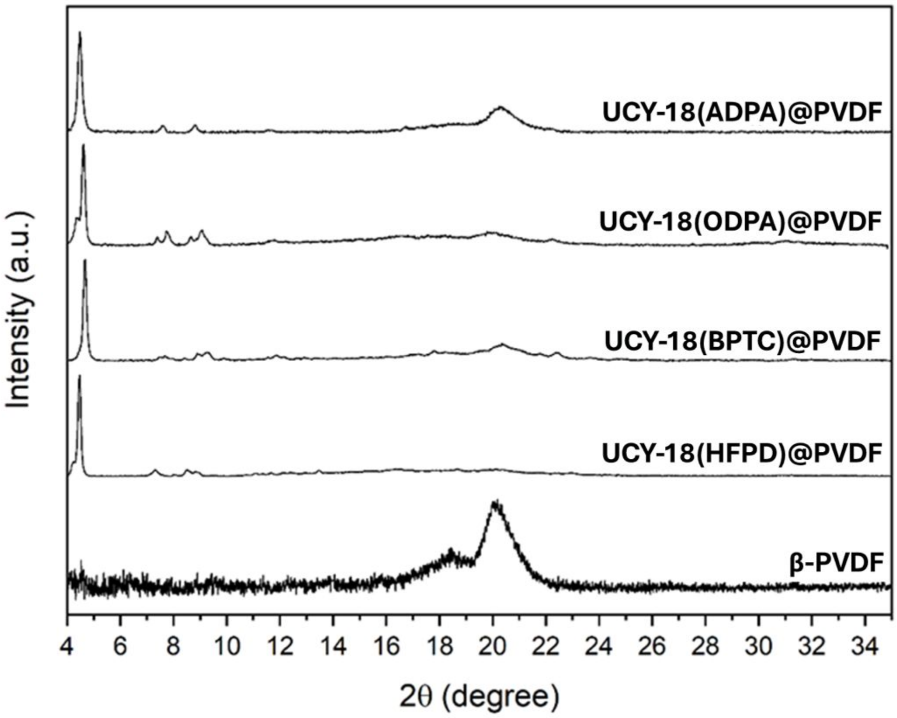

Figure S54. µ-XRD diffractograms of **UCY-18**(L)@PVDF (H_4L= HFPD, BPTC, ODPA, ADPA). The empty PVDF diffractogram is also shown for comparison purposes.

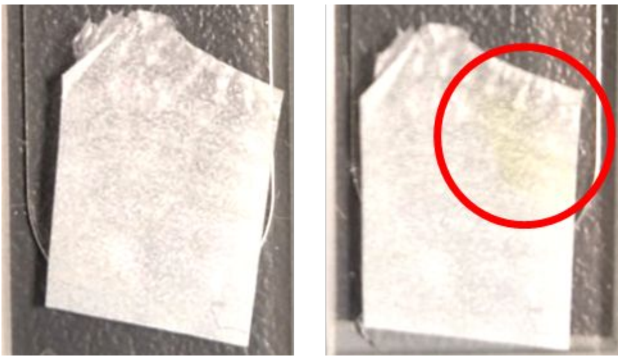

Figure S55. Photograph of a piece of **UCY-18**(BPTC)@PVDF thin film before (left) and after (right) the photoluminescence measurement. The red circle indicates the area of the thin film that was exposed to the incident light beam (upon excitation).

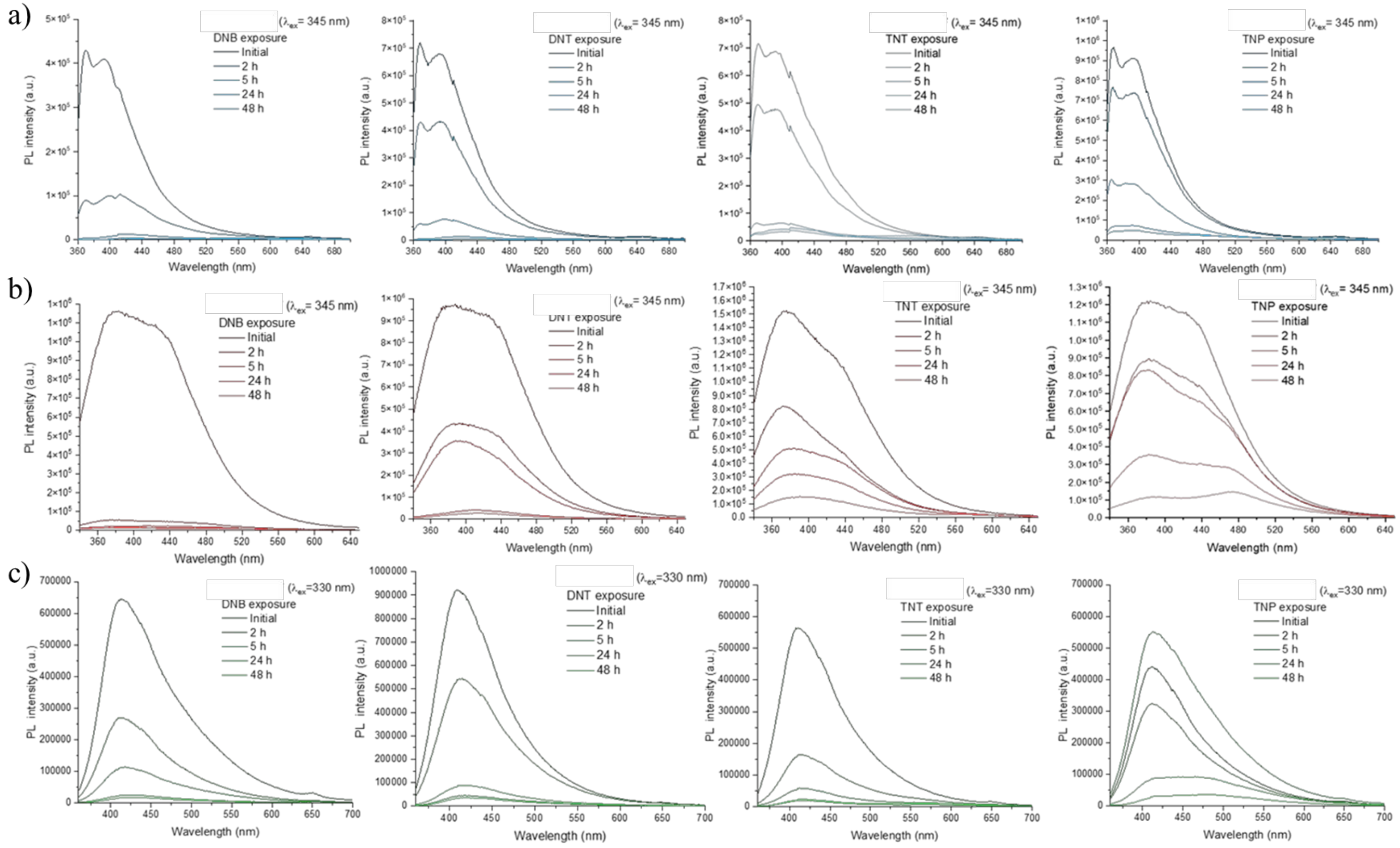

Figure S56. PL emission of a) **UCY-18**(HFPD)@PVDF, b) **UCY-18**(ODPA)@PVDF, and c) **UCY-18**(ADPA)@PVDF after exposure to nitroaromatic vapors (DNB, DNT, TNT and TNT) at different exposure time (0, 2, 5, 24 and 48 hours).

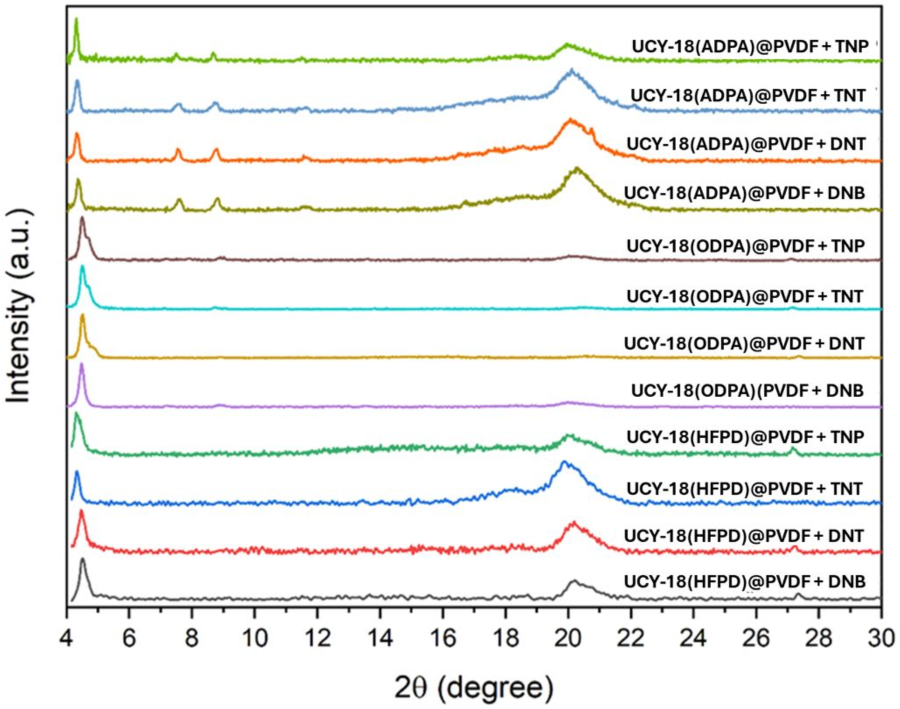

Figure S57. μ-XRD diffractograms of **UCY-18**(HFPD)@PVDF, **UCY-18**(ODPA)@PVDF and **UCY-18**(ADPA)@PVDF after exposure to DNB, DNT, TNT and TNP.

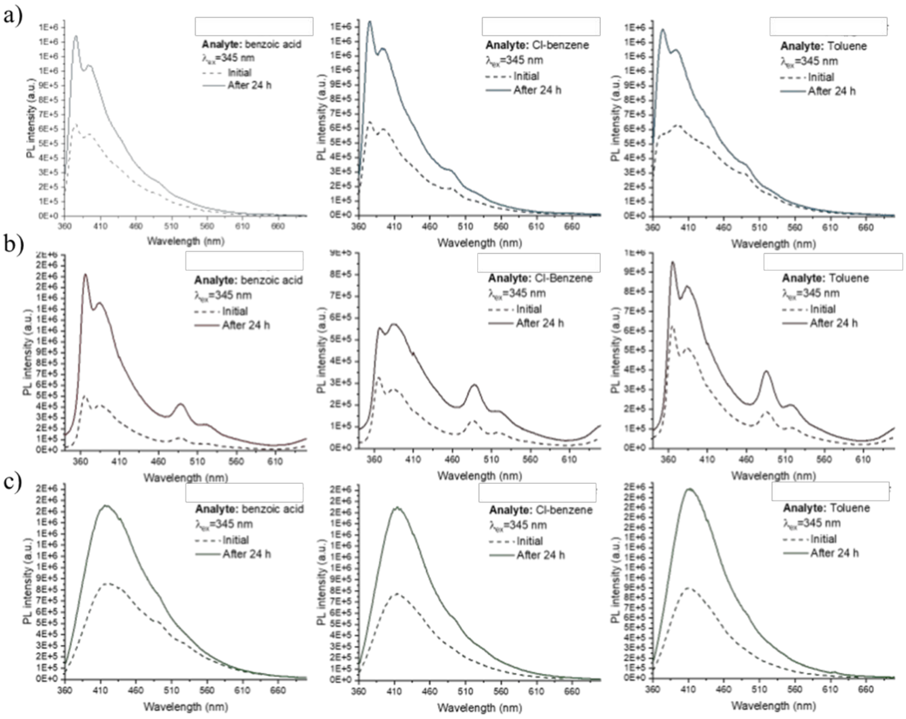

Figure S58. PL emission spectra of a) **UCY-18**(HFPD)@PVDF, b) **UCY-18**(ODPA)@PVDF, and c) **UCY-18**(ADPA)@PVDF after exposure to benzoic acid, Cl-benzene and toluene for 24 h.

SCSC reactions with nitroaromatic compounds

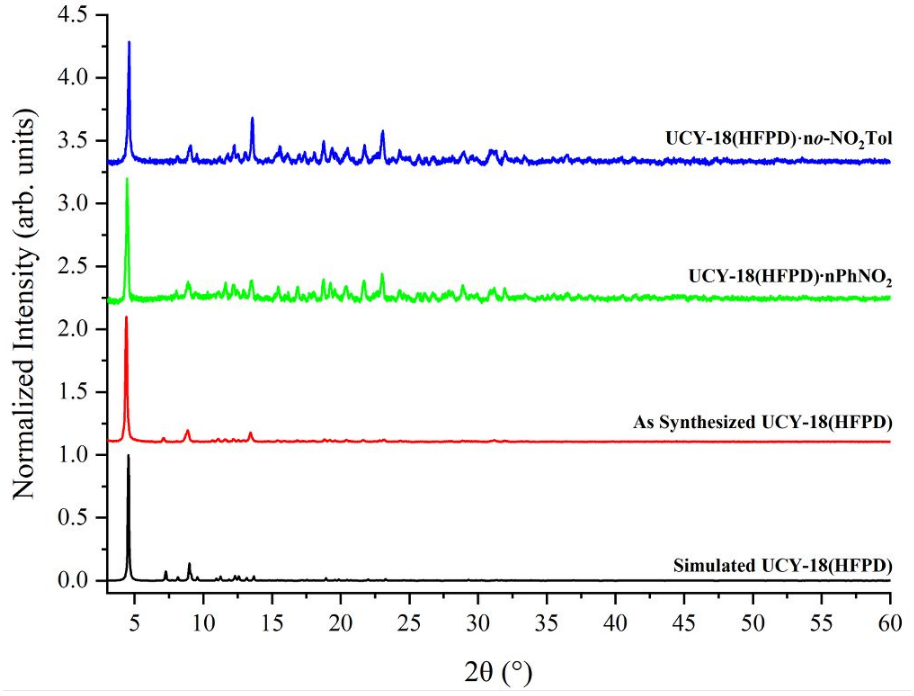

Figure S59. Powder X-ray diffraction patterns of the as synthesized **UCY-18**(HFPD) compound and the exchanged analogues **UCY-18**(HFPD)·nPhNO$_2$ and **UCY-18**(HFPD)·n*o*-NO$_2$Tol.

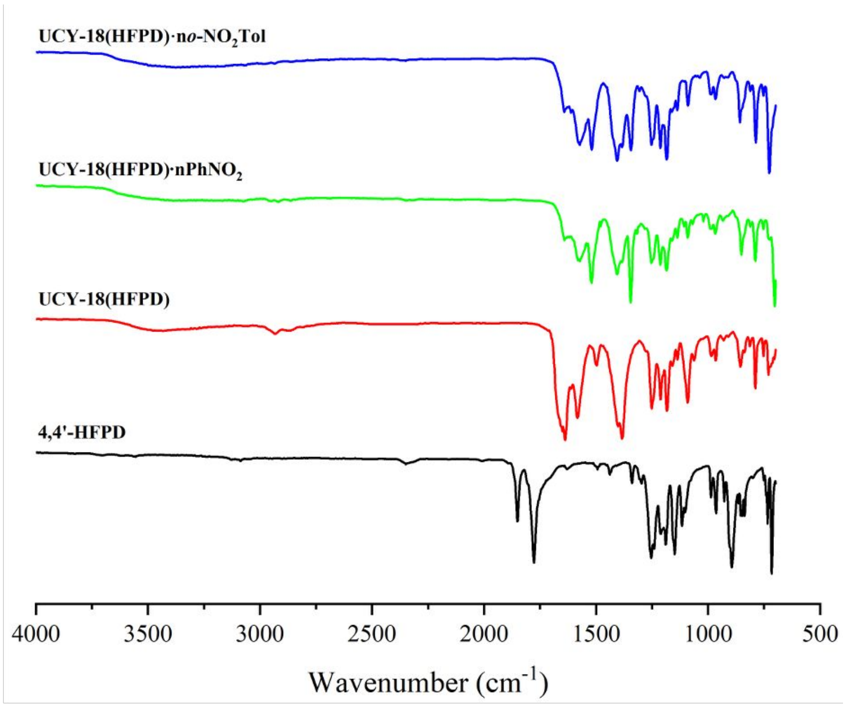

Figure S60. IR spectra of 4,4′-HFPD ligand, the as synthesized **UCY-18**(HFPD) and the exchanged analogues **UCY-18**(HFPD)·nitroaromatic.

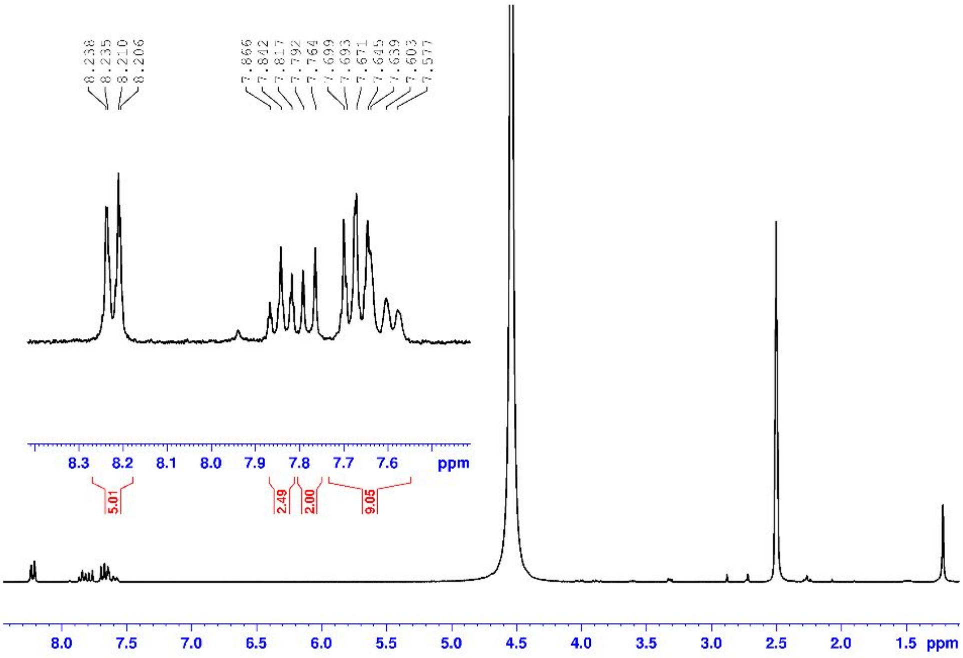

Figure S61. ^{1}H-NMR spectrum of the exchanged analogue **UCY-18**(HFPD)·2.5nPhNO$_2$ digested in 10 μL DCl (35% wt in D$_2$O) in d_6-DMSO. ^{1}H-NMR (d_6-DMSO): δ 7.58-7.70 (m, 4H, Ar-**H**, HFPD^{4-} and 5H, m-Ar-**H**, PhNO$_2$), δ 7.76-7.79 (d, 2H, Ar-**H**, HFPD^{4-}), δ 7.82-7.87 (t, 2.5H, p-Ar-**H**, PhNO$_2$) and δ 8.21-8.24 (d, 5H, o-Ar-**H**, PhNO$_2$).

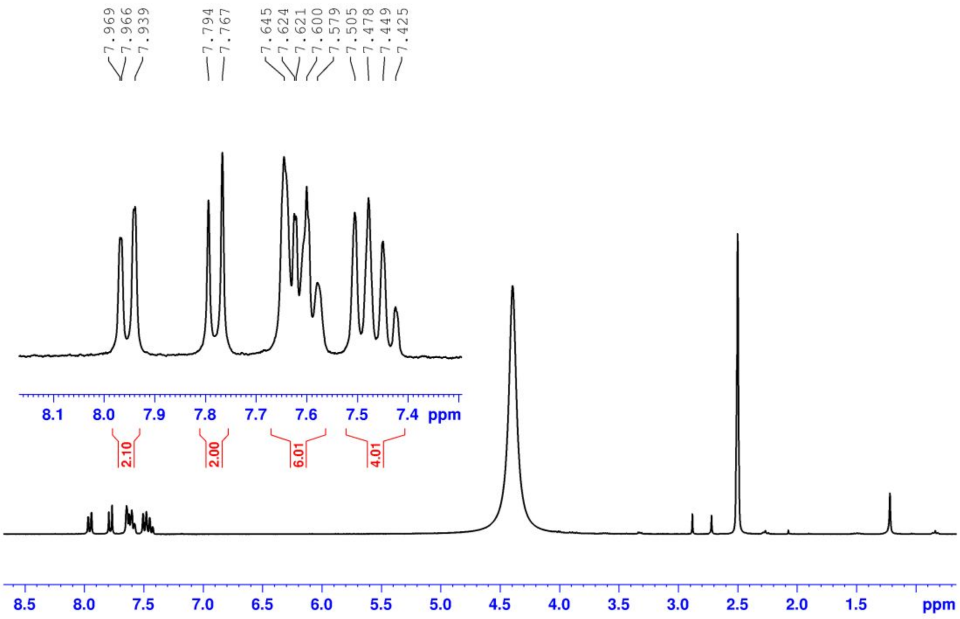

Figure S62. ^{1}H-NMR spectrum of the compound **UCY-18**(HFPD)·2n*o*-NO$_2$Tol digested in 10 μL DCl (35% wt in D$_2$O) in d_6-DMSO. ^{1}H-NMR (d_6-DMSO): δ 7.42-7.51 (m, 4H, Ar-**H**, 2-NO$_2$Tol), δ 7.58-7.64 (m, 4H, Ar-**H**, HFPD^{4-} and 2H, Ar-**H**, 2-NO$_2$Tol), δ 7.78-7.79 (d, 2H, Ar-**H**, HFPD^{4-}) and δ 7.94-7.97 (d, 2H, Ar-**H**, 2-NO$_2$Tol).

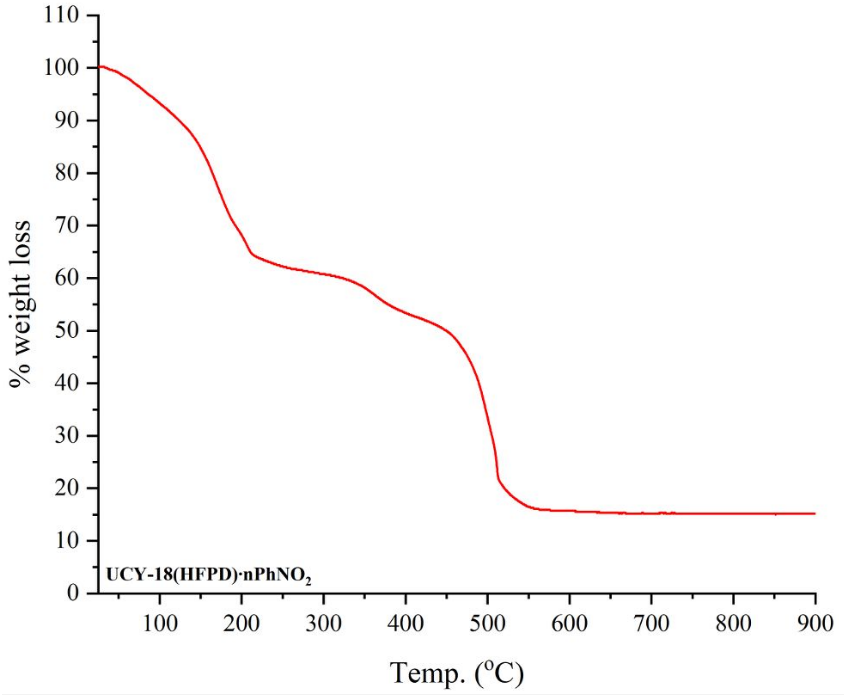

Figure S63. TGA graph of the as synthesized compound **UCY-18**(HFPD)·nPhNO$_2$.

The TGA curve of **UCY-18**(HFPD)·nPhNO$_2$ revealed that the thermal decomposition of this compound proceeds via a multi-step process. The release of guest and coordinated solvent molecules (PhNO$_2$ + H$_2$O) involves continuous mass losses up to 350 °C whereas the last mass loss at higher temperatures is due to the decomposition of the organic ligand HFPD^{4-}. In particular, the mass losses in the temperature range 25 °C to 350 °C correspond to ~37.4 % of the material's total mass and are attributed to the removal of two terminally ligated H$_2$O and 2.5 guest PhNO$_2$ molecules (calc. on the basis of the formula [ZnCa(HFPD)(H$_2$O)$_2$]$_n$·2.5nPhNO$_2$ (**UCY-18**(HFPD)·2.5nPhNO$_2$) 37.1%). The second mass loss, assigned to the decomposition of the organic ligand HFPD^{4-}, occurring in the ~ 350 – 560 °C region corresponds to 47.8% (calc. for **UCY-18**(HFPD)·2.5nPhNO$_2$ 48.0%) of the material's total mass. Lastly, the residual mass (14.8%) at 900 °C corresponds to ZnO + CaO (calc. for **UCY-18**(HFPD)·2.5nPhNO$_2$ 14.9%).

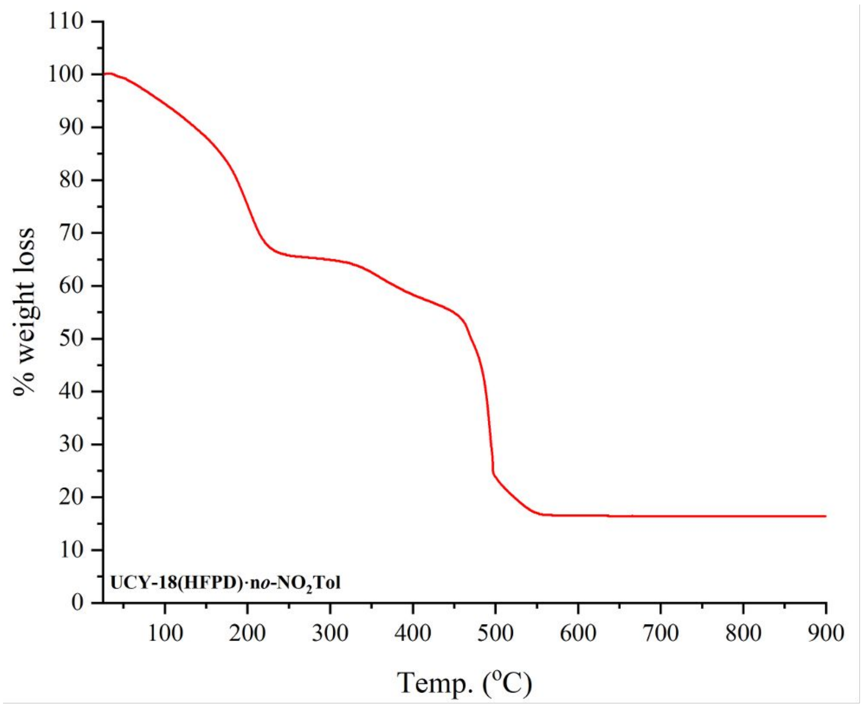

Figure S64. TGA graph of the as synthesized compound **UCY-18**(HFPD)·n*o*-NO_2Tol.

The TGA curve of **UCY-18**(HFPD)·n*o*-NO_2Tol revealed that the thermal decomposition of this compound proceeds via a multi-step process. The release of guest and coordinated solvent molecules (*o*-NO_2Tol + H_2O) involves continuous mass losses up to 340 °C whereas the last mass loss at higher temperatures is due to the decomposition of the organic ligand $HFPD^{4-}$. In particular, the mass losses in the temperature range 25 °C to 340 °C correspond to ~35.0 % of the material's total mass and are attributed to the removal of two terminally ligated H_2O and 2 guest *o*-NO_2Tol molecules (calc. on the basis of the formula $[ZnCa(HFPD)(H_2O)_2]_n$·2n*o*-NO_2Tol (**UCY-18**(HFPD)·2n*o*-NO_2Tol) 34.8%). The second mass loss, assigned to the decomposition of the organic ligand $HFPD^{4-}$, occurring in the ~ 340 – 570 °C region corresponds to 49.9% (calc. for **UCY-18**(HFPD)·2n*o*-NO_2Tol 49.8%) of the material's total mass. Lastly, the residual mass (15.1%) at 900 °C corresponds to ZnO + CaO (calc. for **UCY-18**(HFPD)·2n*o*-NO_2Tol 15.4%).

References

(1) Ji, W. J.; Liu, G. F.; Wang, B. Q.; Lu, W. B.; Zhai, Q. G. Design of a Heterometallic Zn/Ca-MOF Decorated with Alkoxy Groups on the Pore Surface Exhibiting High Fluorescence Sensing Performance for Fe3+and Cr2O72-. *CrystEngComm* **2020**, *22* (28), 4710–4715. https://doi.org/10.1039/d0ce00457j.

(2) Yang, D. L.; Zhang, X.; Yang, J. X.; Yao, Y. G.; Zhang, J. Alkali/Alkaline Earth Metal and Solvents-Regulated Construction of Novel Heterometallic Coordination Polymers Based on a Semirigid Ligand and Tetranuclear Metal Clusters. *Inorganica Chim. Acta* **2014**, *423* (PART A), 62–71. https://doi.org/10.1016/j.ica.2014.07.054.

(3) Chen, H.; Fan, L.; Zhang, X. Highly Robust 3s-3d {CaZn}-Organic Framework for Excellent Catalytic Performance on Chemical Fixation of CO2and Knoevenagel Condensation Reaction. *ACS Appl. Mater. Interfaces* **2020**, *12* (49), 54884–54892. https://doi.org/10.1021/acsami.0c18267.

(4) Ablet, A.; Li, S. M.; Cao, W.; Zheng, X. J.; Jin, L. P. Self-Assembly and Characterization of Ca-Zn Heterometallic MOFs with 4,5-Imidazoledicarboxylate. *Polyhedron* **2014**, *83*, 122–129. https://doi.org/10.1016/j.poly.2014.05.033.

(5) Zhang, X.; Huang, Y. Y.; Cheng, J. K.; Yao, Y. G.; Zhang, J.; Wang, F. Alkaline Earth Metal Ion Doped Zn(Ii)-Terephthalates. *CrystEngComm* **2012**, *14* (14), 4843–4849. https://doi.org/10.1039/c2ce25440a.

(6) Zhang, J. C.; Wang, J. J.; Zeng, S. L.; Wang, Z. M.; Liu, Y.; Zhang, D. J.; Zhang, R. C.; Fan, Y. Syntheses, Characterization, and Luminescent Properties of Ca-Based Metal–Organic Frameworks Based on 1, 4-naphthalene Dicarboxylate. *Inorg. Chem. Commun.* **2018**, *97* (June), 69–73. https://doi.org/10.1016/j.inoche.2018.09.021.

(7) Noh, K.; Ko, N.; Park, H. J.; Park, S.; Kim, J. Two Porous Metal-Organic Frameworks Containing Zinc-Calcium Clusters and Calcium Cluster Chains. *CrystEngComm* **2014**, *16* (37), 8664–8668. https://doi.org/10.1039/c4ce01237b.

(8) Zou, R.; Zhong, R.; Han, S.; Xu, H.; Burrell, A. K.; Henson, N.; Cape, J. L.; Hickmott, D. D.; Timofeeva, T. V.; Larson, T. E.; Zhao, Y. A Porous Metal-Organic Replica of α-PbO2 for Capture of Nerve Agent Surrogate. *J. Am. Chem. Soc.* **2010**, *132* (51), 17996–17999. https://doi.org/10.1021/ja101440z.

(9) Ma, L. F.; Li, B.; Sun, X. Y.; Wang, L. Y.; Fan, Y. T. Hydrothermal Syntheses and Characterizations of Three Zn IIcoordination Polymers Tuned by PH Value and Base. *Zeitschrift fur Anorg. und Allg. Chemie* **2010**, *636* (8), 1606–1611. https://doi.org/10.1002/zaac.200900516.

(10) Wen, G. L.; Han, M. Le; Wang, F. W.; Zhao, X.; Yin, C. Y. A Distorted Pcu Topological Heterometallic Metal-Organic Framework with Right- and Left Chiral Covalent Layers. *Zeitschrift fur Anorg. und Allg. Chemie* **2013**, *639* (12–13), 2307–2311. https://doi.org/10.1002/zaac.201300230.

(11) Bo, Q. B.; Wang, H. Y.; Miao, J. L.; Wang, D. Q. Fluorescent Zn-Based

Hetero-MOFs Design via Single Metal Site Substitution. *RSC Adv.* **2012**, *2* (31), 11650–11652. https://doi.org/10.1039/c2ra21863a.

(12) Dong, Y.; Li, X.; Liu, H. A New (4,8)-Connected Scu-Type Heterometallic-Organic Framework: Synthesis, Structure and Luminescent Property. *J. Inorg. Organomet. Polym. Mater.* **2015**, *25* (4), 645–649. https://doi.org/10.1007/s10904-014-0130-0.

(13) Adeniji, A. O.; Twenter, B. M.; Byrns, M. C.; Jin, Y.; Chen, M.; Winkler, J. D.; Penning, T. M. Development of Potent and Selective Inhibitors of Aldo-Keto Reductase 1C3 (Type 5 17β-Hydroxysteroid Dehydrogenase) Based on N -Phenyl-Aminobenzoates and Their Structure-Activity Relationships. *J. Med. Chem.* **2012**, *55* (5), 2311–2323. https://doi.org/10.1021/jm201547v.

(14) Kanari, N.; Mishra, D.; Gaballah, I.; Dupré, B.; Kanari, N.; Mishra, D.; Gaballah, I.; Dupré, B. Thermal Decomposition of Zinc Carbonate Hydroxide To Cite This Version : HAL Id : Hal-01507382 Thermal Decomposition of Zinc Carbonate Hydroxide. **2017**, *6031* (03), 93–100.

(15) Karunadasa, K. S. P.; Manoratne, C. H.; Pitawala, H. M. T. G. A.; Rajapakse, R. M. G. Thermal Decomposition of Calcium Carbonate (Calcite Polymorph) as Examined by in-Situ High-Temperature X-Ray Powder Diffraction. *J. Phys. Chem. Solids* **2019**, *134* (January), 21–28. https://doi.org/10.1016/j.jpcs.2019.05.023.

(16) Czepirski, L.; JagieŁŁo, J. Virial-Type Thermal Equation of Gas-Solid Adsorption. *Chem. Eng. Sci.* **1989**, *44* (4), 797–801. https://doi.org/10.1016/0009-2509(89)85253-4.

(17) Nuhnen, A.; Janiak, C. A Practical Guide to Calculate the Isosteric Heat/Enthalpy of Adsorption: Via Adsorption Isotherms in Metal-Organic Frameworks, MOFs. *Dalt. Trans.* **2020**, *49* (30), 10295–10307. https://doi.org/10.1039/d0dt01784a.
